# A New Oxygen Containing Pyclen-Type Ligand as a Manganese(II) Binder for MRI and ^52^Mn PET Applications: Equilibrium, Kinetic, Relaxometric, Structural and Radiochemical Studies

**DOI:** 10.3390/molecules27020371

**Published:** 2022-01-07

**Authors:** Tibor Csupász, Dániel Szücs, Ferenc Krisztián Kálmán, Oldamur Hollóczki, Anikó Fekete, Dezső Szikra, Éva Tóth, Imre Tóth, Gyula Tircsó

**Affiliations:** 1 Department of Physical Chemistry, Faculty of Science and Technology, University of Debrecen, Egyetem tér 1, H-4032 Debrecen, Hungary; csupasz.tibor@science.unideb.hu (T.C.); szucs.daniel@science.unideb.hu (D.S.); kalman.ferenc@science.unideb.hu (F.K.K.); holloczki@gmail.com (O.H.); imre.toth@science.unideb.hu (I.T.); 2 Doctoral School of Chemistry, University of Debrecen, Egyetem tér 1, H-4032 Debrecen, Hungary; 3 Department of Medical Imaging, Division of Nuclear Medicine, Faculty of Medicine, University of Debrecen, Egyetem tér 1, H-4032 Debrecen, Hungary; fekete.aniko@science.unideb.hu (A.F.); szikra.dezso@med.unideb.hu (D.S.); 4 Mulliken Center for Theoretical Chemistry, University of Bonn, Beringstr. 4+6, D-53115 Bonn, Germany; 5 Centre de Biophysique Moléculaire, CNRS, Rue Charles Sadron, CEDEX 2, 45071 Orléans, France; eva.jakabtoth@cnrs.fr

**Keywords:** manganese, magnetic resonance imaging, contrast agents, macrocycles, stability, dissociation kinetics, water exchange, radiochemistry

## Abstract

A new pyclen-3,9-diacetate derivative ligand (H_2_**3,9-OPC2A**) was synthesized possessing an etheric O-atom opposite to the pyridine ring, to improve the dissociation kinetics of its Mn(II) complex (pyclen = 3,6,9,15-tetraazabicyclo(9.3.1)pentadeca-1(15),11,13-triene). The new ligand is less basic than the N-containing analogue (H_2_**3,9-PC2A**) due to the non-protonable O-atom. In spite of its lower basicity, the conditional stability of the [Mn(**3,9-OPC2A**)] (pMn = −log(Mn(II)), c_L_ = c_Mn(II)_ = 0.01 mM. pH = 7.4) remains unaffected (pMn = 8.69), compared to the [Mn(**3,9-PC2A**)] (pMn = 8.64). The [Mn(**3,9-OPC2A**)] possesses one water molecule, having a lower exchange rate with bulk solvents (*k*_ex_^298^ = 5.3 ± 0.4 × 10^7^ s^−1^) than [Mn(**3,9-PC2A**)] (*k*_ex_^298^ = 1.26 × 10^8^ s^−1^). These mild differences are rationalized by density-functional theory (DFT) calculations. The acid assisted dissociation of [Mn(**3,9-OPC2A**)] is considerably slower (*k*_1_ = 2.81 ± 0.07 M^−1^ s^−1^) than that of the complexes of diacetates or bisamides of various 12-membered macrocycles and the parent H_2_**3,9-PC2A**. The [Mn(**3,9-OPC2A**)] is inert in rat/human serum as confirmed by ^52^Mn labeling (nM range), as well as by relaxometry (mM range). However, a 600-fold excess of EDTA (pH = 7.4) or a mixture of essential metal ions, propagated some transchelation/transmetalation in 7 days. The H_2_**3,9-OPC2A** is labeled efficiently with ^52^Mn at elevated temperatures, yet at 37 °C the parent H_2_**3,9-PC2A** performs slightly better. Ultimately, the H_2_**3,9-OPC2A** shows advantageous features for further ligand designs for bifunctional chelators.

## 1. Introduction

Magnetic resonance imaging (MRI) is an important tool in modern diagnostic medicine. During MRI investigations, paramagnetic complexes, the so-called contrast agents (CAs), are applied in order to generate increased intensity differences. The delineation of different soft-tissues and abnormalities within a certain tissue type, relies on the differences in terms of the longitudinal (*T*_1_) and/or transverse (*T*_2_) relaxation times of the protons present in tissues (a substantial part of it belongs to the solvent, i.e., water). The majority of commercial MRI CAs are Gd(III) complexes (GBCAs), which operate by mainly shortening the longitudinal (*T*_1_) relaxation time.

**Figure 1:**
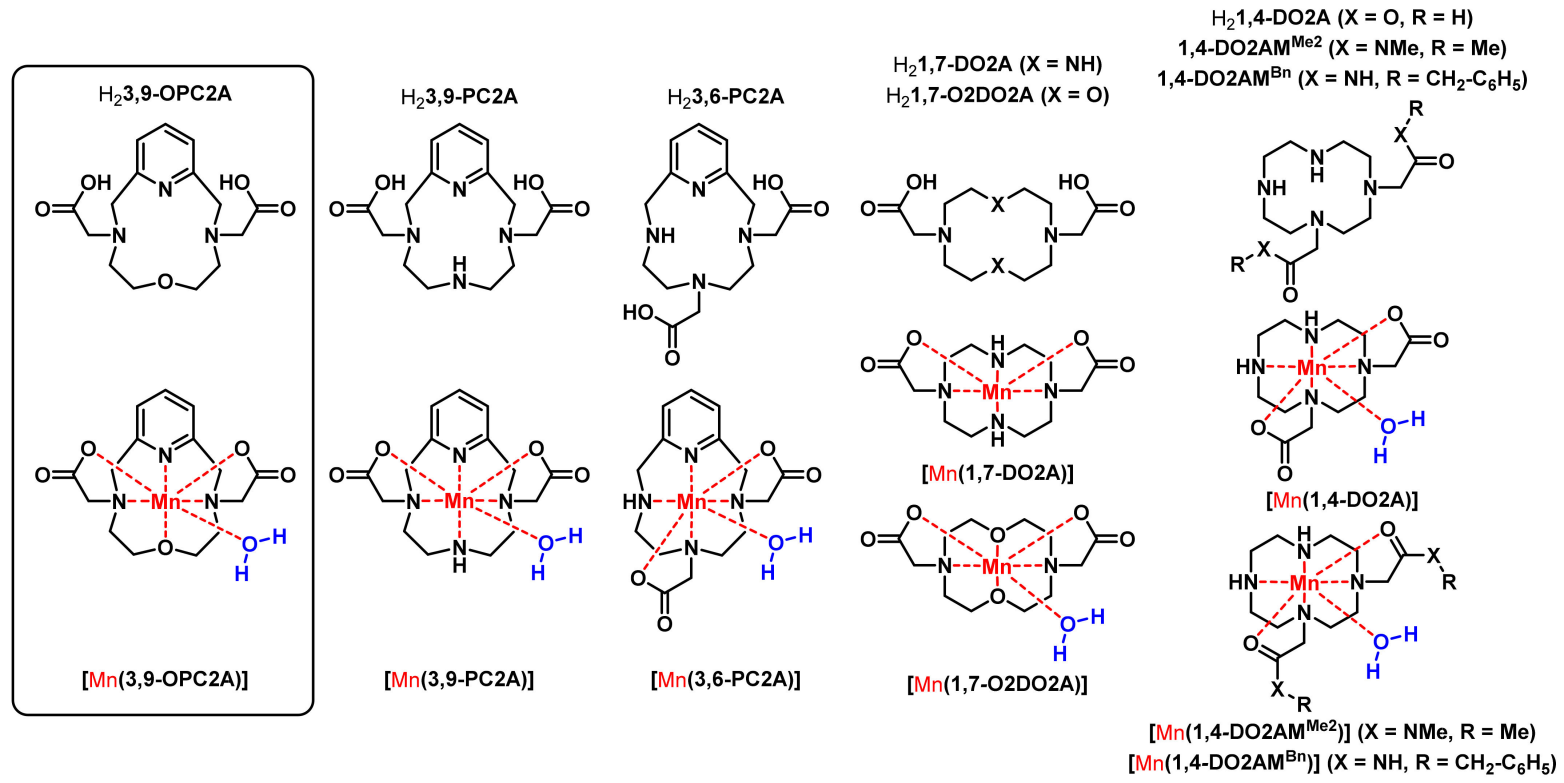
Formulae of the ligands and their Mn(II) complexes, discussed in the present work.

Since their introduction (starting from mid 1980s), Gd(III) complexes have been considered among the safest diagnostic agents. However, the trust in Gd(III)-based agents has suffered considerably, since a direct link was identified between the progressive, potentially fatal disease called nephrogenic systemic fibrosis (NSF) and the use of certain (nearly exclusively linear ligand-based) GBCAs in patients with renal failure (since early 2000s) [1,2]. Restrictions introduced by the regulatory agencies (such as the European Medicines Agency (EMA) and U.S. Food and Drug Administration (FDA)) in 2007, helped to prevent new NSF cases [3]. Lately, evidence has also been gathered on brain and bone accumulation of Gd(III) in patients with normal renal function following multiple CA administration. These findings raise public concerns about the safety of Gd(III) agents and have spurred research towards exploring safe surrogates to Gd(III), in addition to other approaches aiming at the improvement of CA efficiency to allow for dose reduction, or designing more stable/inert Gd(III) chelates. Complexes of paramagnetic essential metal ions, such as Mn(II), Fe(II) or Fe(III), are among the most widely studied candidates in this respect. When applied at elevated concentrations required for MRI applications, Mn(II) compounds also display toxicity [4]. Therefore, Mn(II)-based agents must also be administered in the form of stable and inert complexes, while the metal bound water molecule is required to achieve high relaxivities (increase in the relaxation rate propagated by the presence of 1 mM paramagnetic substance). Unfortunately, the thermodynamic stability and kinetic inertness of Mn(II) complexes are generally lower than that of Gd(III) complexes, or those of other transition metal ion complexes, due to the smaller charge and the lack of ligand-field stabilization energy (high-spin *d*^5^ electron configuration). Mn(II) complexes formed with linear ligands are kinetically labile; a few exceptions known in the literature involve the rigid H_4_***trans*-CDTA** (Scheme S1) and its derivatives (i.e., H_3_**PyC3A**) or H_4_**PhDTA** (Scheme S1) complexes (these Mn(II) complexes have a dissociation half-life of 10–20 h near physiological pH) [5,6,7]. Although these structural entities have been “converted” into bifunctional ligand structures required for bioconjugation and targeting purposes, recently, certain applications (immuno-PET studies and smart/responsive CA candidates) require complexes that are substantially more inert [6,8]. Formation of such complexes are expected if macrocyclic ligands are used. However, the majority of macrocyclic Mn(II) complexes either possess a low relaxivity (because of the lack of metal-bound water molecules) while being inert (e.g., [Mn(**DOTA**)]^2−^ and [Mn(**PCTA**)]^−^, Scheme S1), or they possess low inertness as observed for the bisaquated Mn(II) complexes formed with pentadentate **15Py-aneN_5_** and **15Py-aneN_3_O_2_** macrocyclic ligands and their structural analogs (Scheme S1) [9,10,11]. A compromise between these seemingly contradictory requirements was recently achieved by us and M. Botta and coworkers [12,13], concerning the Mn(II) complexes of H_2_**1,4-DO2A** (Figure 1) and its amide derivatives such as **1,4-DO2AM^Me2^** (*t*_1/2_ of the dissociation are in the order of 50 and 550 h at pH = 7.4, respectively). These chelates possess an average of 0.9 bound water molecules; thus, the relaxivity of their Mn(II) complexes is modest, and the bis(amide) derivatives appears to be more inert than the parent [Mn(**1,4-DO2A**)] chelate (*t*_1/2_ of the dissociation is 550 h at pH = 7.4) [13]. Applying known avenues for attaining high relaxivities (i.e., decreasing the tumbling rate of the complex by the non-covalent binding of the chelates to slowly tumbling macromolecular platforms, such a proteins and dendrimers) resulted in Mn(II) complexes with improved relaxation properties (**1,4-Bn_2_DO2AM^Me^** or **1,4-DO2AM^Bn^**, Scheme S1). The strong non-covalent binding of these Mn(II) complexes to human serum albumin (HSA), can allow the visualization of the vascular system, similar to that observed for the **MS-325** used in the clinical practice as an angiographic agent [14]. As far as the inertness of Mn(II) chelates is concerned, the best candidate so far has been proposed by Toth and coworkers. The ligand used by the authors is based on a rigid bispidine platform, and the monoaquated Mn(II) complex formed by their ligand possesses an inertness similar to that of [Mn(**DOTA**)]^2−^ [15].

Our approach to Mn(II)-based MRI CA candidates is based on the rigid pyclen derivative H_2_**3,9-PC2A** ligand (H_2_**3,9-PC2A** = pyclen-3,9-diacetate), as it forms a stable monoaquated complex with Mn(II) [16]. However, its inertness requires further improvement as [Mn(**3,9-PC2A**)] is readily protonated, favoring an efficient dissociation via the proton-assisted mechanism (as confirmed by the calculation of the electrostatic potential using DFT methods). However, for the H_2_**3,9-PC2A** derivatives recently proposed as smart imaging probes (SCAs) for detecting tissue pH sensing Zn(II) ions or for angiographic imaging (H_2_**3,9-PC2A-EA**, H_2_**3,9-PC2A-BP** and H_2_**3,9-PC2A-DPA**, respectively, Scheme S1) the required inertness is achieved by the attachment of ethylamine (possessing picolyl moieties for Zn(II) sensing) or biphenyl moieties; herein, we intend to eradicate the most basic protonation site of the ligand [17,18,19]. It is achieved by the replacement of one amine nitrogen in the macrocycle for an etheric oxygen atom. This structural modification can also positively affect the hydration number of the Mn(II) ion in the complexes, in a similar manner that was observed for the diacetate of 1,4-diaza-7-oxacyclononane (9-membered 1,4,7-triazacyclononane derivative ligand), or for [Mn(**1,7-O2DO2A**)] in relation to [Mn(**1,7-DO2A**)]—the former complex possesses one metal ion bound water molecule, while none are present in [Mn(**1,7-DO2A**)] (Figure 1) [20,21].

In addition, due to the availability of the long-lived positron emitting ^52^Mn isotope (that can be used in immunoPET, or is applicable for cell tracking, β-cell mass monitoring, and nanomedicines), extensive research is conducted with the aim of finding a suitable chelator for its complexation [22,23]. Due to the long circulation times of radiolabeled mAbs (immunoPET), the inertness of ^52^Mn radiochelates needs to be explored in detail. Thus, in the present paper, we report the synthesis of a newly designed H_2_**3,9-OPC2A** ligand and the equilibrium study of its complexes formed with the most abundant essential metal ions, (Mg(II), Ca(II), Zn(II), and Cu(II)), as well as Mn(II). We also report the dissociation kinetics of the Mn(II) complex in the presence of competing Cu(II) and Zn(II), as characterized by the rates of metal exchange reactions, as well as the serum stability of [Mn(**3,9-OPC2A**)]. A detailed ^1^H and ^17^O NMR (nuclear magnetic resonance) relaxometric study is performed to understand the factors governing the water exchange, while DFT calculations are performed to access the structure of the complex that is compared to that of [Mn(**3,9-PC2A**)]. Radiolabeling of the structurally analogous H_2_**3,9-OPC2A** and H_2_**3,9-PC2A** ligands with ^52^Mn are also performed, in order to assess the applicability of these platforms in ^52^Mn-based PET applications.

## 2. Results and Discussion

### 2.1. Synthesis of the Ligand

**Scheme 1:**
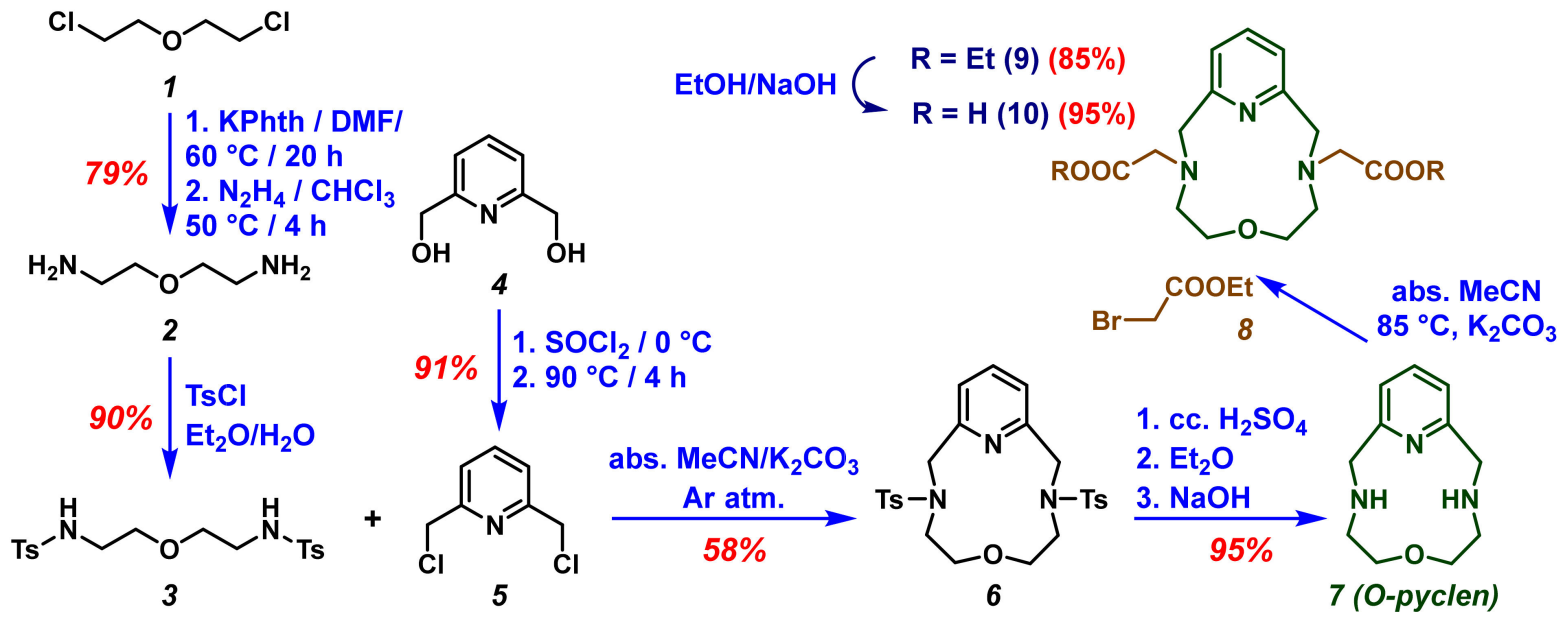
Synthesis route of H_2_**3,9-OPC2A** macrocycle.

Synthesis of the ditosylate **6** was accomplished by E. Weber and F. Vögtle [24], some time ago; however, we examined several approaches and optimized the preparation of the macrocycle. The synthesis of O-pyclen was performed by the condensation of **3** and **5** compounds (Scheme 1). For the preparation of **3**, commercially available bis(2-chloroethyl)ether (**1**) was used as a starting material. The amine function was obtained by Gabriel synthesis using potassium phthalimide [25], and the amino groups of the diamine **2** were protected with the tosyl protecting group (The given protecting group is expected to reduce the number of by-products during the cyclization.). Dichloride **5** was synthesized following the method suggested by H. Su and coworkers [26], with slight modifications. The macrocyclization step to obtain **6,** was carried out in dry solvent under an inert atmosphere using K_2_CO_3_ as a base. Several bases (Na_2_CO_3_, K_2_CO_3_, Cs_2_CO_3_, DIPEA, and TEA) were tested to improve the yield of the macrocyclization, since, along with the strength of the base, the template effect of the cation was also reported to be very important to achieve the desired macrocycles [27,28]. The deprotection of the tosyl groups by using strong acid (conc. H_2_SO_4_) at elevated temperatures, afforded the O-pyclen (**7**) in a nearly quantitative yield. 

The preparation of H_2_**OPC2A** was achieved via the alkylation of the secondary amino groups of macrocycle **7**, by reacting them with various amounts of ethyl-2-bromoacetate (**8**) in anhydrous acetonitrile in the presence of a K_2_CO_3_ base under an inert atmosphere. The alkylating agent was added in several portions (Table 1) to the reaction mixture, and the conversion was followed by the analytical HPLC technique. The monosubstituted (**3-OPCA^OEt^**) and disubstituted (**3,9-OPC2A^OEt^**) products formed during the reaction, appeared to have different retention times in HPLC using the reversed stationary phase, so we could obtain information about the ratio of the formed products (Table 1). During the evaluation of the chromatogram we considered the peak areas of the starting O-pyclen macrocycle, **3-OPCA^OEt^** and **3,9-OPC2A^OEt^** products. The formation of other by-products was negligible in this reaction. The products were purified using the preparative HPLC technique, and MS and NMR measurements were carried out to identify the compounds. The ethyl protecting groups of the macrocycle **9** were sapanoficated using NaOH to achieve the Na_2_**3,9-OPC2A**, which was converted to its acidic form (H_2_**3,9-OPC2A** (**10**)) during the purification procedure using the preparative HPLC technique. The ^1^H-NMR, ^13^C-NMR and MS spectra, as well as the analytical HPLC chromatograms of the products, can be found in the Supplementary Information (Figures S1–S20).

### 2.2. Equilibrium Studies

The protonation constants of H_2_**OPC2A** have been determined by pH-potentiometry in 0.15 M NaCl ionic medium, and the constants determined are listed in Table 2, along with the corresponding values determined for structurally similar ligands used as comparative benchmarks (H_2_**3,9-PC2A**, H_2_**3,6-PC2A**, H_2_**1,4-DO2A**, H_2_**1,7-DO2A**, and H_2_**1,7-O2DO2A**).

The first two protonation events for these ligands occur at the donor atoms of the macrocycle (N-atoms), while those occurring at a more acidic pH can be attributed to the carboxylate groups. The pyclen macrocycle and its derivatives (triacetate derivative H_3_**PCTA**, for instance) exhibit a rather unique protonation sequence: the first protonation occurs at the nitrogen atom positioned trans to the pyridine ring, while, during the second protonation step (involving one *cis*-N-atom), the first proton shifts to the second *cis-*nitrogen atom for a better charge separation [29]. Therefore, the replacement of the amine -NH- group by an etheric -O- atom is expected to affect not only the protonation constant values (as it is observed on going form H_2_**1,7-DO2A** ligand towards the H_2_**1,7-O2DO2A** chelator), but the protonation sequence as well, since the most basic N-atom of the pyclen is missing in O-pyclen [20]. As it can be seen from the data presented in Table 2, the highest protonation constant for the H_2_**3,9-OPC2A** ligand is log *Κ*_1_^H^ = 7.73, while the second protonation can be characterized by a nearly identical constant (log *K*_2_^H^ = 7.66). Based on these values, the H_2_**3,9-OPC2A** ligand possesses the lowest basicity (considering the sum log *K*_1_^H^ + log *K*_2_^H^, although H_2_**3,9-OPC2A** and its analogs possess more protonation constants). A similar decrease in the protonation constants/ligand basicity has been evidenced for the H_2_**1,7-DO2A** derivative H_2_**1,7-O2DO2A** ligand, and was rationalized by the replacement of two nitrogen donor atoms in the cyclen backbone for oxygen atoms [20]. The overall basicity of the series of structurally related ligands follows the trend H_2_**1,7-DO2A** > H_2_**1,4-DO2A** > H_2_**3,6-PC2A** > H_2_**3,9-PC2A** > H_2_**1,7-O2DO2A** ≈ H_2_**3,9-OPC2A**. As these ligands can be treated as structurally related chelators (as far as the size of the macrocycle, nature, and amount of donor atoms are concerned), one can expect a similar trend for the stability constants of their complexes.

The stability and protonation constants of the metal complexes formed with H_2_**OPC2A** were determined by pH-potentiometric titrations, but, for the Cu(II) complex, UV-Vis spectrophotometric measurements were also carried out to supplement the pH-potentiometric titration data (Figures S21 and S22, Supporting Information).

**Figure 2:**
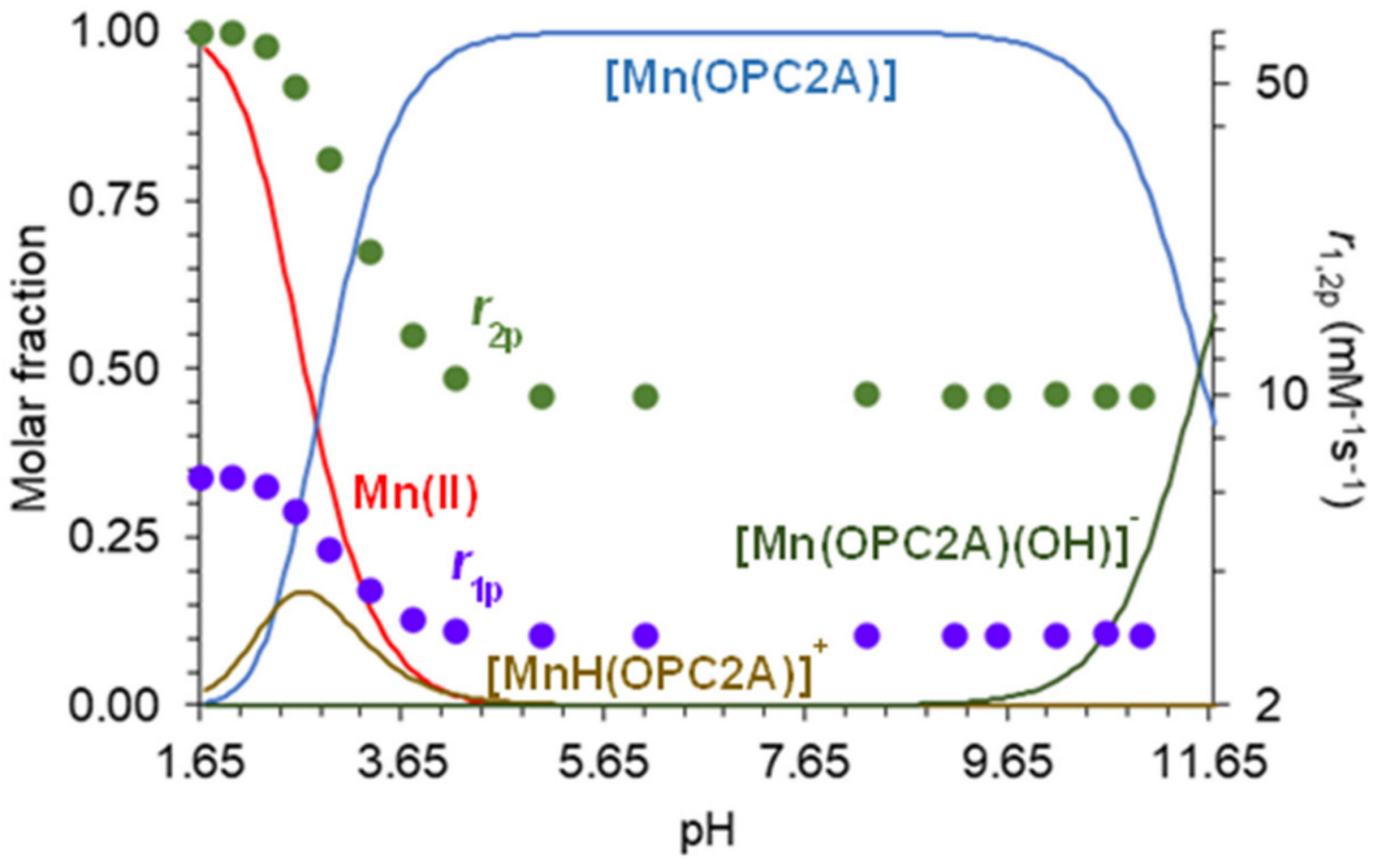
Species distribution curves calculated for the [Mn(**3,9-OPC2A**)], along with the pH dependence of its relaxivity (*r*_1p_: ● and *r*_2p_: ●) (T = 25 °C, *I* = 0.15 M NaCl, 1.41 T).

The validity of the equilibrium model used for the fitting of the pH-potentiometric titration data for the Mn(II) system, was supported by ^1^H relaxometric measurements, when 1/*T*_1_ and 1/*T*_2_ relaxation rates were determined as a function of pH, and their trends were compared to the species distribution curves calculated by using the equilibrium constants determined by pH-potentiometry (Table 3). The equilibrium for all metal ions can be described by considering the formation of only ([M(L)]) and monoprotonated ([M(HL)]) complexes (Table 3). However, for the Mn(II), Cu(II), and Zn(II) ions, the formation of ternary hydroxido species ([M(L)(OH)]) was also considered. A comparison of the pH-relaxation rate profiles normalized to 1 mM with the species distribution curves, confirmed that the equilibrium model applied for the fitting of pH-potentiometric titration data, satisfactorily described the Mn(II) system. As it can be seen in Figure 2, the [Mn(**3,9-OPC2A**)] complex exhibits constant relaxivity (2.88 mM^−1^s^−1^ at 1.41 T, and 25 °C) in a wide pH range (5.0–11.0). The given value is similar to that of macrocyclic Mn(II) complexes possessing 0.9 [(Mn(**1,4-DO2A**)]), i.e., one coordinated water molecule (*q* = 1) in their inner coordination sphere (i.g. [Mn(**3,6-PC2A**)], [Mn(**3,9-PC2A**)], or [Mn(**1,7-O2DO2A**]), and they were higher than the relaxivity of complexes with *q* = 0 (typically 1.4–1.6 mM^−1^s^−1^ as observed for [Mn(**DO3A**)]^−^ or [Mn(**PCTA**)]^−^ [12,16,20,21]. Lowering the pH below pH = 4.0, resulted in a sharp relaxivity increase, reaching the *r*_1p_ value of 6.51 mM^−1^s^−1^ (at 1.41 T and 25 °C) near pH = 1.8. The increase in relaxivity can be associated to the protonation of the complex, followed by its dissociation at a low pH, reaching the *r*_1p_ values characteristic of the [Mn(H_2_O)_6_]^2+^ aqua complex under these conditions (magnetic field and temperature). The formation of the [Mn(**3,9-OPC2A**)] complex was also confirmed by analytical HPLC at pH 7.0 relying on the different retention times of the ligand and the complex (the difference is more than 2 min (Figures S20 and S23)).

The stability constants of the complexes are summarized and compared to those of the chelates formed with H_2_**1,7-DO2A**, H_2_**1,4-DO2A**, H_2_**3,6-PC2A**, H_2_**3,9-PC2A**, and H_2_**1,7-O2DO2A**, in Table 3. H_2_**3,9-OPC2A** forms considerably weaker complexes with all the metal ions involved in the study, than H_2_**3,9-PC2A**, H_2_**3,6-PC2A**, H_2_**1,7-DO2A**, and H_2_**1,4-DO2A** chelators. However, despite the very similar basicity of the H_2_**1,7-O2DO2A** and H_2_**3,9-OPC2A** ligands, the stability of [M(**3,9-OPC2A**)] is notably higher, which can likely be attributed to the rigidity of the ligand. A similar trend in the stability constants can be observed, when the stability of the structurally related ligands is compared (H_2_**3,9-PC2A** vs. H_2_**1,7-DO2A** and, at a somewhat lesser extent, in the case of H_2_**3,6-PC2A** vs. H_2_**1,4-DO2A** ligands). It needs to be emphasized that it can be erroneous to compare the stability of complexes formed with ligands of a different basicity, as they possess different conditional stability near the physiological pH. Therefore, we have calculated the pMn values (pMn = −log(Mn(II)_free_) for the Mn complexes, by using the conditions proposed by É. Tóth and coworkers (c_Mn^2+^_ = c_ligand_ = 1 × 10^−5^ M, T = 25 °C, pH = 7.4) [11]. As it is seen from the data presented in Table 3, the value characteristic for the [Mn(**3,9-OPC2A**)] complex is as high (pMn = 8.69) as it is for the [Mn(**3,9-PC2A**)] chelate (despite its lower stability constant), which is, in fact, the highest known value for a monoaquated macrocyclic Mn(II) complex (pMn = 8.64). This means that more than 99,97% of the total Mn(II) in the system is chelated by the H_2_**3,9-OPC2A** ligand near pH = 7.4 in equilibrium. Therefore, it can be concluded that the conditional stability of the complex did not suffered from the replacement of >NH by an etheric oxygen atom (-O-) in the rim of the pyclen macrocycle.

### 2.3. Relaxivity of the Mn(II) Complex

The relaxivity of Mn(II) complexes is one of the most important parameters that directly correlates with its MRI efficacy. Relaxivity is defined as the longitudinal (*r*_1p_) and transverse (*r*_2p_) water proton relaxation rate enhancement, provided by 1.0 mM concentration of the paramagnetic species. The relaxivity (*r*_1p_ and *r*_2p_) of the Mn(II) complexes were determined using relaxometers operating at 0.49 T and 1.41 T field strength, in a HEPES buffer (*I* = 0.15 M NaCl) at different temperatures from the slopes of plots 1/*T*_1,2_ vs. (Mn(II)) for 4 concentrations of Mn(II) (data shown in Supporting Information Figures S24 and S25), and they are compared in Table 4.

The relaxivity of [Mn(**3,9-OPC2A**)] at all temperatures and field strengths studied are slightly higher than those of the complexes used as comparative benchmarks. This correlates well with the structural difference of the ligand (i.e., the replacement of the secondary amino group by an ether -O- atom), which can affect the number and the exchange rate of the coordinated water (see later, the Section dealing with the ^17^O and NMRD measurements). Similar relaxivity improvement has been previously observed, when applying similar modifications to the structurally related cyclen-based [Mn(**1,7-DO2A**)] (which does not possess a metal-bound water molecule [12]), while the [Mn(**1,7-O2DO2A**)] complex possesses a metal-bound water molecule [20].

### 2.4. 17. O and NMRD Measurements

The relaxivity of paramagnetic metal complexes is governed by several parameters, including the water exchange rate (*k*_ex_), the rotational correlation time (*τ*_R_), and the number of inner sphere water molecules (*q*). In order to obtain information for these parameters, temperature dependent ^17^O NMR measurements (9.4 T) and nuclear magnetic relaxation dispersion (NMRD) experiments were carried out in the frequency range of 0.01–80 MHz, at 3 different temperatures (25, 37, and 50 °C). Since numerous physico-chemical parameters determine the relaxivity, the independent assessment of some of the parameters is essential for the correct characterization. The variable temperature ^17^O NMR measurements can deliver information about *k*_ex_ and *τ*_R_ via the transverse (1/*T*_2_) and the longitudinal (1/*T*_1_) ^17^O relaxation rates, respectively, while the ^17^O chemical shifts (Δ*ω*) depend on *q*. Therefore, the transverse and longitudinal relaxation rates and chemical shifts for a [Mn(**3,9-OPC2A**)] complex and a diamagnetic reference sample (HClO_4_, pH = 3.3) have been determined in the aqueous solution. Since the *T*_1_ values and the chemical shifts do not provide significant differences between the Mn(II) complex and the reference, those are not included in the calculations. 

The ^17^O 1/*T*_2r_ values and the NMRD data have been analyzed simultaneously by the Swift–Connick equations [31], as well as by the Solomon–Bloembergen–Morgan (SMB) [32] and Freed models [33] (see Supporting Information). In this calculation, the water exchange rate, *k*_ex_^298^; its activation enthalpy and entropy, Δ*H*^‡^ and Δ*S*^‡^; the rotational correlational time, *τ*_R_; its activation energy, *E*_R_; the square of the zero-field splitting energy (ZFS), *Δ*^2^; and the correlation time of its modulation, *τ*_v_, have been determined and listed in Table 5, together with corresponding values of other complexes for comparison. As a result of the high number of parameters involved in the calculations, some have to be set to reasonable estimates based on literature data. Thus, the scalar coupling constant, *A*/*ħ*_O_, is set to 33 × 10^6^ rad s^−1^, the distance between the metal ion, and the inner and outer sphere water protons are fixed to *r*_MnH_ = 2.83 Å and *a*_MnH_ = 3.6 Å, respectively; the diffusion coefficient and its activation energy are fixed to *D*_MnH_ = 23 × 10^−10^ m^2^s^−1^ and *E*_MnH_ = 18 kJmol^−1^, and the activation energy of the modulation of the zero-field-splitting, *E*_v_ to 1.0 kJmol^−1^.

**Figure 3:**
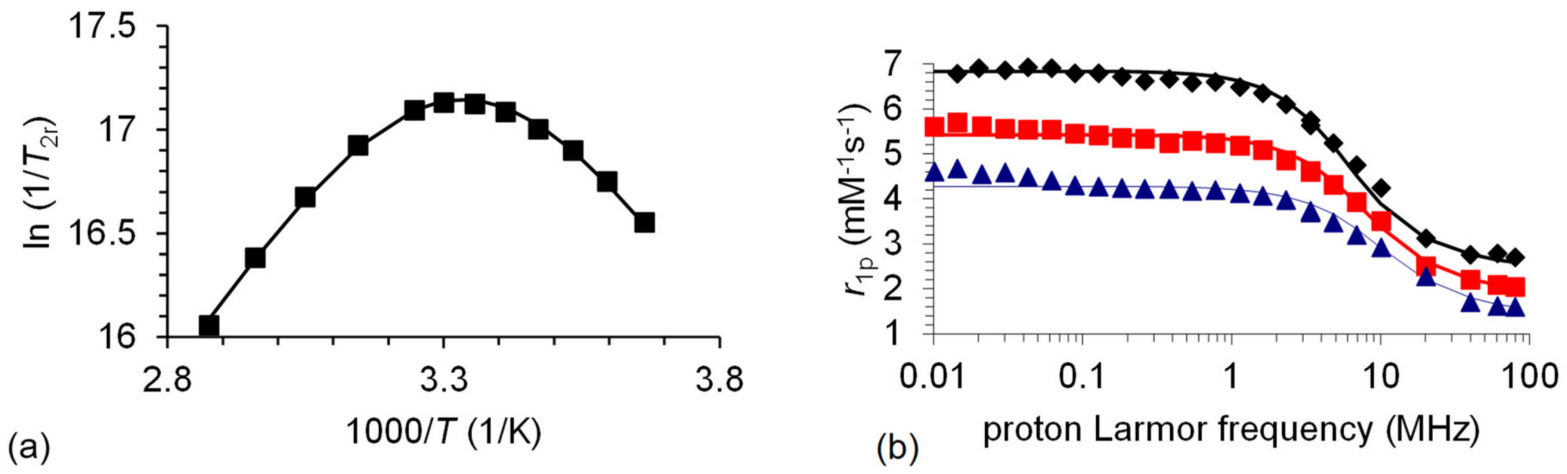
(**a**) Variable temperature reduced transverse ^17^O relaxation rates (9.4 T) recorded for [Mn(**3,9-OPC2A**)]. The line corresponds to the fits of the data. (**b**) ^1^H NMRD profiles of [Mn(**3,9-OPC2A**)] at 25 (♦), 37 (■), and 50 (▲) °C. The curves are the fitted ones.

The hydration number has been fixed to *q* = 1, based on the relaxivity value measured at 20 MHz (*r*_1_ = 3.09 mM^−1^s^−1^), and on the value, 0.97 ± 0.2, determined for *q* using the method proposed by Gale et al. [34]. The experimental data and the fitted curves are shown in Figure 3.

As it is demonstrated by Figure 3a, the reduced transverse relaxation rates (1/*T*_2r_) are in the fast exchange regime above 298 K, while they turn to the intermediate and slow region below that temperature. In the slow exchange regime, the water exchange rate is directly (and accurately) determined from the 1/*T*_2r_ values. The *k*_ex_^298^ value of [Mn(**3,9-OPC2A**)] is less than the half of that determined for the [Mn(**3,6-PC2A**)] and [Mn(**3,9-PC2A**)] chelates, which can only be the result of the substitution of the *trans*-N of the macrocycle to an O donor atom. This modification causes an increase in the rigidity of the coordination cage and significantly inhibits the internal rearrangement, which is necessary for the water exchange process. Water exchange occurs 20 times faster on [Mn(**1,4-DO2A**)] [21] than on [Mn(**3,9-OPC2A**)], due to the more flexible structure of the former, but the amidation of the pendant arms of H_2_**1,4-DO2A** brings the *k*_ex_^298^ values of [Mn(**1,4-DO2AM^Me2^**)]^2+^ [13] and [Mn(**3,9-OPC2A**)] complexes close. Interestingly, the [Mn(**3,9-OPC2A**)] and [Mn(**1,7*-*O2DO2A**] chelates show an identical water exchange rate. The activation entropy obtained for [Mn(**3,9-OPC2A**)] is close to zero (Δ*S*^‡^ = −1.9 ± 0.8 JK^−1^mol^−1^), suggesting an interchange mechanism for the water exchange.

The NMRD profiles recorded for [Mn(**3,9-OPC2A**)] have the typical shape of low-molecular-weight complexes with a single dispersion between 1–10 MHz (Figure 3b). The *r*_1p_ values decrease with the increasing temperature in the overall frequency range, indicating that the relaxivity is controlled by fast rotation, as it has earlier been observed for several small Mn(II) complexes [21,35,36]. The rotational correlation time (*τ*_rH_^298^) is similar for the 5 complexes that were compared, and reflects their similar molecular size.

### 2.5. Kinetic Studies

The kinetic inertness of the complexes considered for in vivo applications is an important parameter. It can be characterized by using various approaches. One of the possibilities is to study the rates of dissociation of the complexes, by following the transmetalation reactions occurring between the complex and the large excess (applied to ensure pseudo-first-order conditions) of a suitable exchanging metal ions (essential metal ions, such as Zn(II) or Cu(II) are often used for this purpose). The rates of dissociation reactions are explored as a function of pH (in a wide pH range) as well as in a wide metal ion concentration range, in order to obtain information about the contribution of various mechanisms (spontaneous, acid-, or metal-assisted dissociation). Such an extensive study can be time-consuming (especially when the complex is inert); therefore, P. Caravan and coworkers [6] suggested the study of the dissociation of the complexes at pH = 6.0 (set by 50 mM MES buffer), by following the transmetalation of the complex propagated by 25 equivalents of Zn(II) at 37 °C and comparing the pseudo-first-order rate constants (or the dissociation half-lives). Owing to the high *r*_2p_ relaxivity of the free Mn(II) ion released as a result of dissociation (64.3 and 54.2 mM^−1^s^−1^ at 1.41 T and 25 and 37 °C, respectively), measuring the relaxation rates as a function of time is a very sensitive method to probe the inertness of Mn(II) chelates. Results obtained for different complexes under the same conditions can be compared, and the order of relative inertness can be accessed rapidly. Finally, in the field of nuclear medicine, the “serum stability” is often determined, which, in fact, reflects dissociation in the blood serum (i.e., the dissociation initiated by the serum components). Upon the dissociation of the complex, the Mn(II) reacts with the serum components (HSA for instance), which results in a considerable relaxivity enhancement caused by the formation of Mn(II) species with a longer rotational correlation time (a remarkable increase in molecular mass). Therefore, ^1^H relaxometry is an easily accessible tool for following the transmetalation/transchelation reactions of paramagnetic complexes occurring in blood serum.

Metal exchange reactions occurring with Zn(II) at pH = 6.0: first, we evaluated the rate of the metal exchange reaction occurring with Zn(II) under the conditions proposed by P. Caravan and coworkers [6], in order to have an idea of how the replacement of the secondary amine group for an etheric oxygen in the rim of the pyclen macrocycle affects the inertness of its corresponding Mn(II) complex. The plot of the 1/*T*_2_ values as a function of time for the [Mn(**3,9-OPC2A**)] and [Mn(**3,9-PC2A**)] complexes are shown in Figure S26 (Supporting Information). Fitting the measured data for the [Mn(**3,9-OPC2A**)] by the monoexponential function gives the rate constant *k*_obs_ = (1.74 ± 0.04) × 10^−5^ s^−1^. The value is circa 20 times smaller than that previously reported for [Mn(**3,9-PC2A**] (*k*_obs_ = (3.28 ± 0.03) × 10^−4^ s^−1^), and nearly 40 times lower than that of [Mn(**PyC3A**)] (*k*_obs_ = (6.76 ± 0.04) × 10^−4^ s^−1^), a Mn(II) chelate recently proposed as a possible Gd(III) alternative CA candidate. These preliminary results show that the substitution of the secondary amino group in H_2_**3,9-PC2A** for an etheric oxygen atom (H_2_**3,9-OPC2A**) considerably improves the inertness of the formed Mn(II) complex.

**Figure 4:**
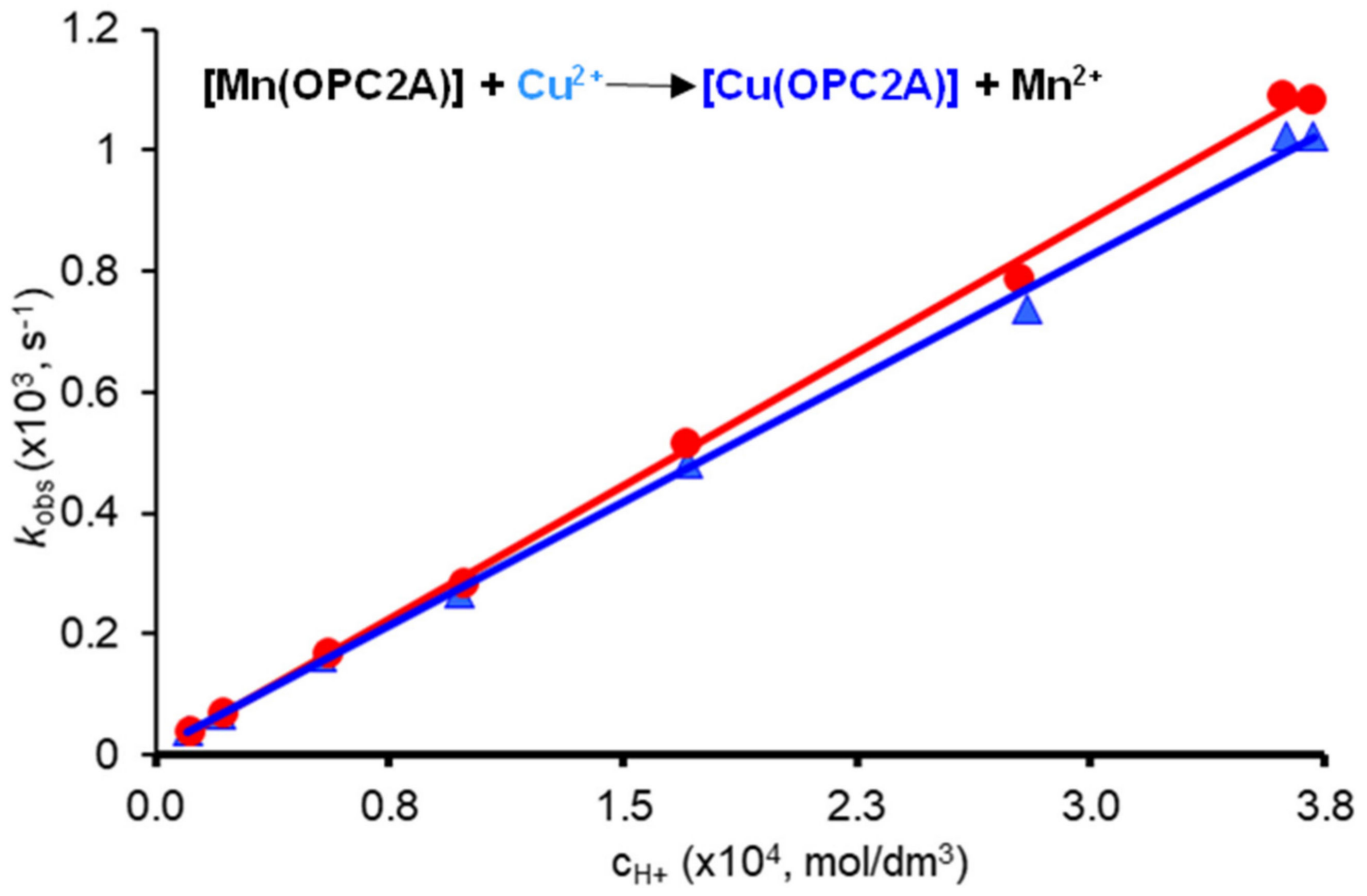
Plot of the pseudo-first-order rate constants (*k*_obs_) as a function of [H^+^] ion concentration for [Mn(**3,9-OPC2A**)], observed by using different exchanging metal ion concentrations (20-fold (●) and 40-fold (▲) Cu(II) excess).

Metal exchange reactions occurring with Cu(II) studied as a function of pH: a more detailed dissociation kinetic study was performed by using the Cu(II) ion. Metal exchange reactions occurring with Cu(II) were followed by UV-Vis as a function of the pH (in the pH range of 3.44–4.97) and metal ion concentration (20- and 40-fold excess of Cu(II) was applied), in order to evaluate the rates of spontaneous (*k*_0_ rate constant) and/or proton assisted (*k*_1_ rate constant) and/or metal ion provoked dissociation (*k*_M_). The pseudo-first-order rate constants determined by fitting the time–absorbance curves are shown in Figure 4.

As it can be seen in Figure 4, the rate constants (*k*_obs_) are proportional to the H^+^ ion concentration, while they slightly decrease with the increasing Cu(II) concentration. Therefore, one can assume that [Mn(**3,9-OPC2A**)] dissociates by following spontaneous (the positive value of the intercept with the y axis) and proton assisted pathways, while Cu(II) is involved in the formation of a dinuclear intermediate with a relatively low stability [Mn(**3,9-OPC2A**)Cu]^2+^, which is a “dead-end” complex (i.e., its formation reduces the concentration of the reactive protonated species, thereby slowing down the reaction). The dissociation pathways followed by [Mn(**3,9-OPC2A**)] can be accounted for by Equation (1), where [Mn(L)]_t_ is the total concentration of the Mn(II) complex, and the *k*_0_, *k*_H_*,* and *k*_M_ are the rate constants characterizing the spontaneous, the proton and metal ion assisted pathways, respectively.

(1) $$-\frac{d[\mathrm{Mn}(L)]}{\mathrm{dt}}=k_{\mathrm{obs}}{\left[\mathrm{Mn}(L)\right]}_{t}=k_{0}[\mathrm{Mn}(L)]+k_{H}[\mathrm{Mn}(\mathrm{HL})]+k_{H}^{H}\left[\mathrm{Mn}\left(H_{2}L\right)\right]+k_{M}[\mathrm{Mn}(L)\mathrm{Cu}]$$

Using the expressions for the stability and protonation constants of the complexes, and that of the dinuclear intermediate [Mn(L)Cu], one can derive Equation (1) (in this expression *k*_1_ = *k*_H_ × *K*_MnL__×H_; *k*_2_ = *k*_H_^H^ × *K*_MnL__×H_; × *K*_MnLH__×H_; *k*_3_ = *k*_M_ × *K*_MnL__×Cu_; where *K*_MnL__×H_; and *K*_MnLH__×H_ are the protonation constants of [Mn(**3,9-OPC2A**)] and *K*_MnL__×Cu_ is the stability constant of the dinuclear [Mn(**3,9-OPC2A**)Cu]^2+^ intermediate, which is used for the fitting of the *k*_obs_ rate data—the proton assisted dissociation pathways involving a diprotonated intermediate, characterized *k*_2_, play a negligible role in the dissociation of the [Mn(**3,9-OPC2A**)] complex under the experimental conditions used for kinetic studies.

(2) $$k_{\mathrm{obs}}=\frac{k_{0}+k_{1}\left[H^{+}\right]}{1+K_{H}\left[H^{+}\right]+K_{M}\left[{\mathrm{Cu}}^{2+}\right]}$$

The rate and equilibrium constants obtained by fitting the pseudo-first-order rate constants to Equation (2) are shown and compared with those of the complexes used as comparative benchmarks in Table 6. As it is seen from the data presented in Table 6, the rate constant characterizing the proton assisted dissociation of the H_2_**3,9-OPC2A** complex is, by far, the smallest among the Mn(II) complexes formed with structurally related diacetate ligands. In fact, the proton-assisted dissociation is less efficient at promoting the dissociation of the [Mn(**3,9-OPC2A**)] complex, as the *k*_1_ value was found to be considerably smaller (*k*_1_ = 2.81 vs. 8.7 M^−1^s^−1^) than that of the positively charged [Mn(**1,4-DO2AM^Me2^**)]^2+^ complex, as evidenced by Forgács and coworkers (H_2_**1,4-DO2AM^Me2^** is a bis(amide) derivative of the H_2_**1,4-DO2A** chelator, synthesized with the aim of improving the dissociation kinetic properties based on the data evidenced for Gd(III) complexes in the literature. (Figure 1)) [13]. Similar conclusions can be obtained by comparing the half-lives of the dissociation extrapolated to pH = 7.4 (Table 6 where *t*_1/2_^pH = 7.4^ = ln2/*k*_obs_^pH = 7.4^ using c_Zn(II)_ being equal to 1 × 10^−5^ M), calculated with the aim of directly comparing the inertness of different Mn(II) complexes (which can be difficult, when solely based on the rate constants as the contribution of different reaction pathways to the dissociation of Mn(II) complexes in the presence of various amounts of metal ions at a different pH). The data shown in (Table 6) confirm that the [Mn(**3,9-OPC2A**)] possesses very promising dissociation kinetic properties, as its inertness is considerably higher than that of all other related systems, including the [Mn(**1,4-DO2AM^Me2^**)]^2+^ complex. However, we can obtain a seemingly reliable rate constant data characterizing the spontaneous dissociation, which has a strong effect on the rate of dissociation of the complex (or its half-life) at a high pH, in which the contribution of the acid-assisted dissociation is less pronounced. Whereas, one can observe a noticeable difference when comparing the half-life of dissociation at pH = 7.4, measured using large Zn(II) excess (4.81 × 10^−6^ s^−1^ that translates to a half-life of 40.0 h) with that extrapolated using the rate constants determined by studying the Cu(II) exchange reactions (*t*_1/2_ = 22.8 h). A possible explanation for this is that the contribution of the spontaneous dissociation is much smaller than expected, based on the calculation involving the rate constants (this is in line with the studies recently performed for the Mn(II) complexes of macrocyclic ligands, as, in the majority of the cases, the contribution of the spontaneous dissociation was not observed). Therefore, the value of *k*_0_ is suggested to be treated as the higher limit of the given rate constant.

Serum “stability” of the [Mn(**3,9-OPC2A**)] chelate: during MRI investigations, the CAs are injected into the vein of the patients and, following their injection, the complexes can meet with different proteins present in the blood serum, which are known to bind the essential metal ions in relatively strong manner. Therefore, the plasma proteins can potentially initiate transmetalation/transchelation reactions, especially in the case of Mn(II) complexes whose thermodynamic stability is lower than the stability of the chelates formed with essential metal ions, such as Zn(II) or Cu(II). What is more, for some Mn(II) complexes, depending upon the stability constants, of course, the Ca(II) ions present at high concentrations in the serum can initiate the dissociation of the complex, as in the case of [Mn(**EGTA**)]^2^^−^, [Mn(**BAPTA**)]^2^^−^, or [Mn(**CaM**)], for instance [37]. In nuclear medicine, serum “stability” studies are routinely performed, in order to access the integrity of the chelates in the given competitive media. Luckily, ^1^H relaxometry can also be applied for the same purpose because the Mn(II) dissociating from its complexes forms an adduct with HSA, possessing high relaxivities (considerably higher than that of the free Mn(II) or its chelate). Upon the binding of Mn(II) to HSA, the relaxivity is expected to increase from 7.92 mM^−1^s^−1^ (characteristic for the Mn(II) ion in the pure aqueous solution) to 97.2 mM^−1^s^−1^ (at 0.47 T and 25 °C) or 76.35 mM^−1^s^−1^ (at 1.41 T and 25 °C) [38,39]. Our results indicate that the relaxation rate of the samples containing the [Mn(**3,9-OPC2A**)] chelate remained constant for at least 80 h, indicating the negligible dissociation of the complex in serum solution prepared from Seronorm (Figure S27, Supporting Information). One should note, however, that the relaxivity of the complex in the Seronorm solution slightly increased (*r*_1p_ is 5.09 mM^−1^s^−1^ at 1.41 T and 25 ^°^C), compared to the values obtained in the pure aqueous solution (2.70 mM^−1^s^−1^ at 1.41 T and 25 ^°^C), indicating a weak interaction of the uncharged complex with some of the serum components.

### 2.6. Radiochemistry

Antibodies are increasingly applied in modern medicine, for diagnostics and targeted monoclonal antibody (mAb) therapy. Positron-emitting ^52^Mn (*t*_1/2_ = 5.59 days) is an excellent tool for the given purpose. Due to the long circulation times of radiolabeled mAbs, the inertness of the radiochelates (attached to them as diagnostic/therapeutic ”bullets”) can be the bottleneck in the way of their application. Therefore, we designed experiments to investigate the chelation properties of the newly synthesized H_2_**3,9-OPC2A** ligand and the related H_2_**3,9-PC2A** chelators with ^52^Mn radioisotopes. The stability of the radiolabeled complexes was also examined under different conditions, in order to gain information about the applicability of the newly synthesized platform in ^52^Mn-based PET applications.

**Figure 5:**
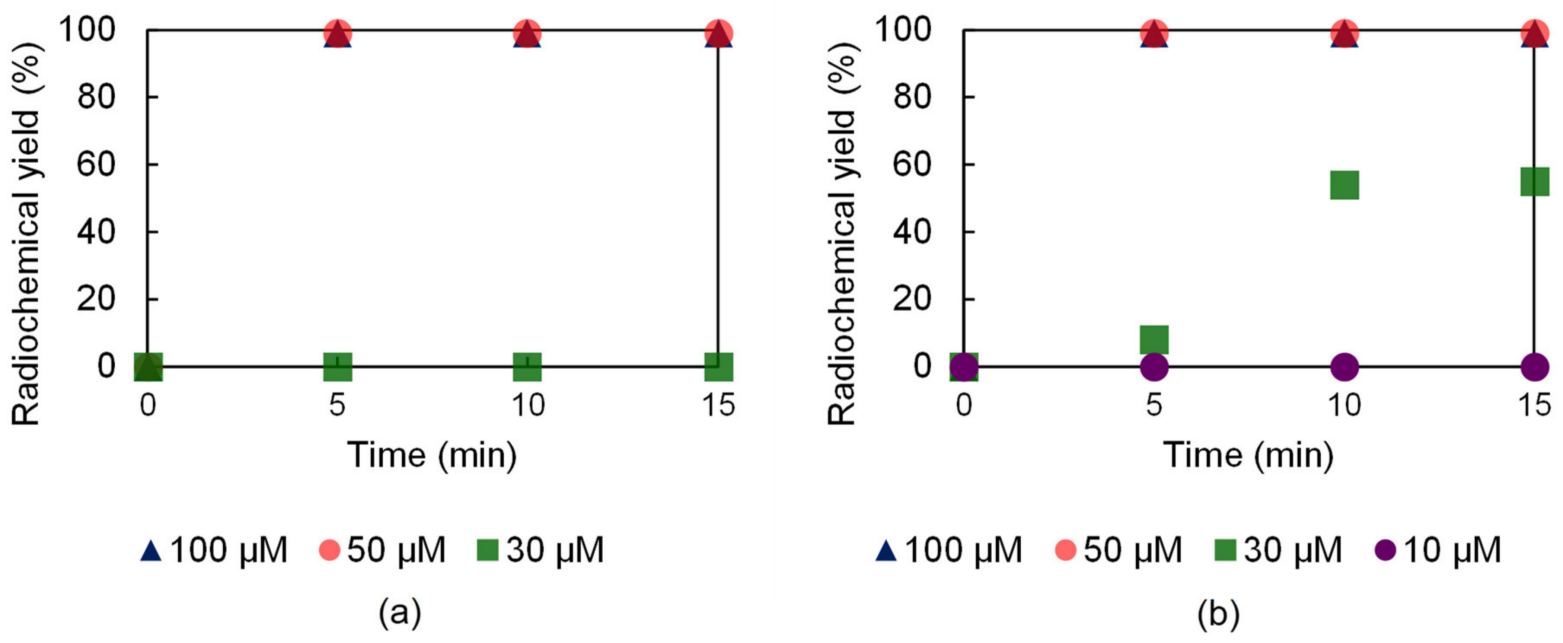
^52^Mn labeling effectiveness of H_2_**3,9-PC2A** (**a**) and H_2_**3,9-OPC2A** (**b**) at 95 °C.

The ^52^Mn labeling performance of the H_2_**3,9-OPC2A** and H_2_**3,9-PC2A** ligands was investigated using different ligand concentrations (10, 25, 30, 50, 75, 100, and 150 µM), at 95 and 37 °C. The radiolabeling of both ligands, by applying 50 µM ligand concentration, was almost complete in 5 min at 95 °C (Figure 5). However, in the case of H_2_**3,9-OPC2A** at 30 µM ligand concentration, a slow formation kinetics was noticed, whereas, in the case of H_2_**3,9-PC2A**, this ligand concentration was not high enough to achieve radiolabeling (Figure 5).

**Figure 6:**
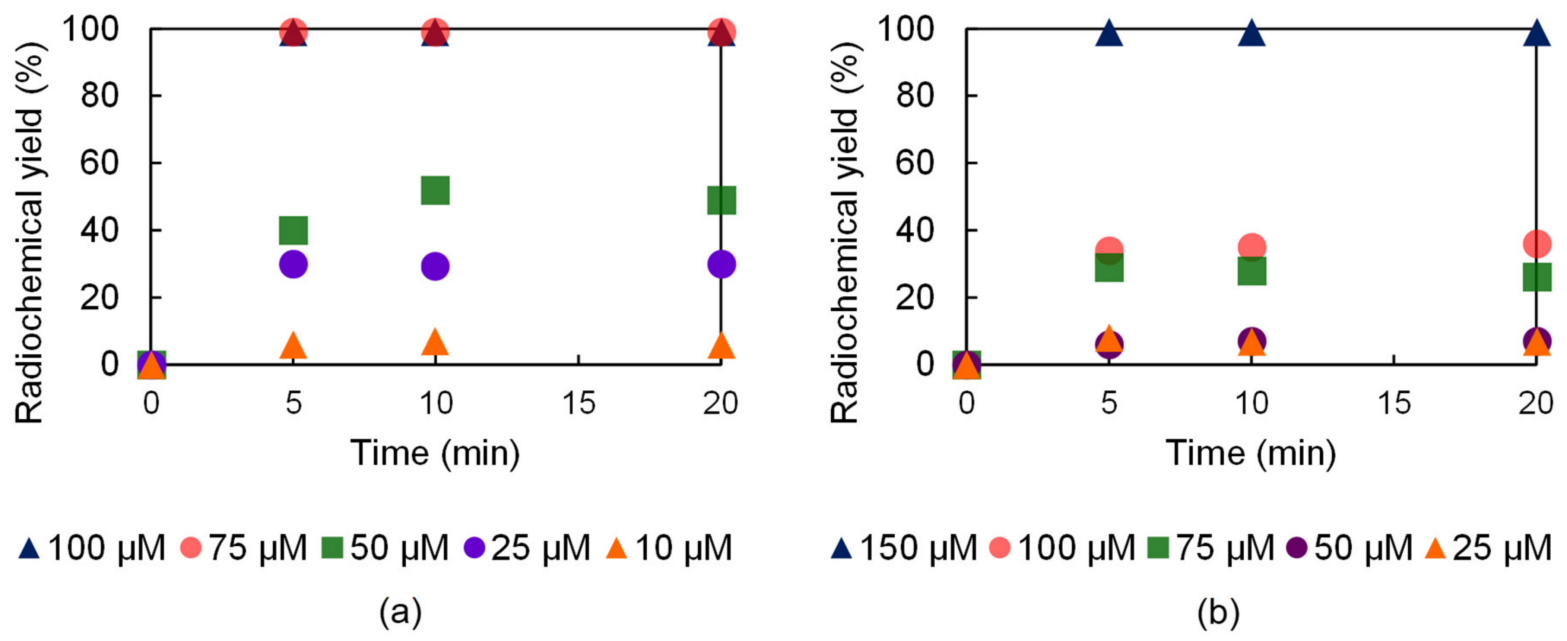
^52^Mn labeling effectiveness of H_2_**3,9-PC2A** (**a**) **and** H_2_**3,9-OPC2A** (**b**) at 37 °C.

The labeling efficiency of the H_2_**3,9-PC2A** and H_2_**3,9-OPC2A** ligands was also examined at 37 °C, since this temperature was more suitable for antibody labeling. Both ligands were able to chelate the ^52^Mn isotope at 37 °C, but these radiolabeling reactions required higher ligand concentrations. The complete radiolabeling of the H_2_**3,9-PC2A** ligand with ^52^Mn was achieved at 75 µM ligand concentration in 5 min at 37 °C, whereas, under these conditions, a considerably higher (150 µM) ligand concentration was necessary in the case of H_2_**3,9-OPC2A**. Interestingly, at lower ligand concentrations, the longer reaction time did not improve the radiochemical yield (Figure 6).

**Figure 7:**
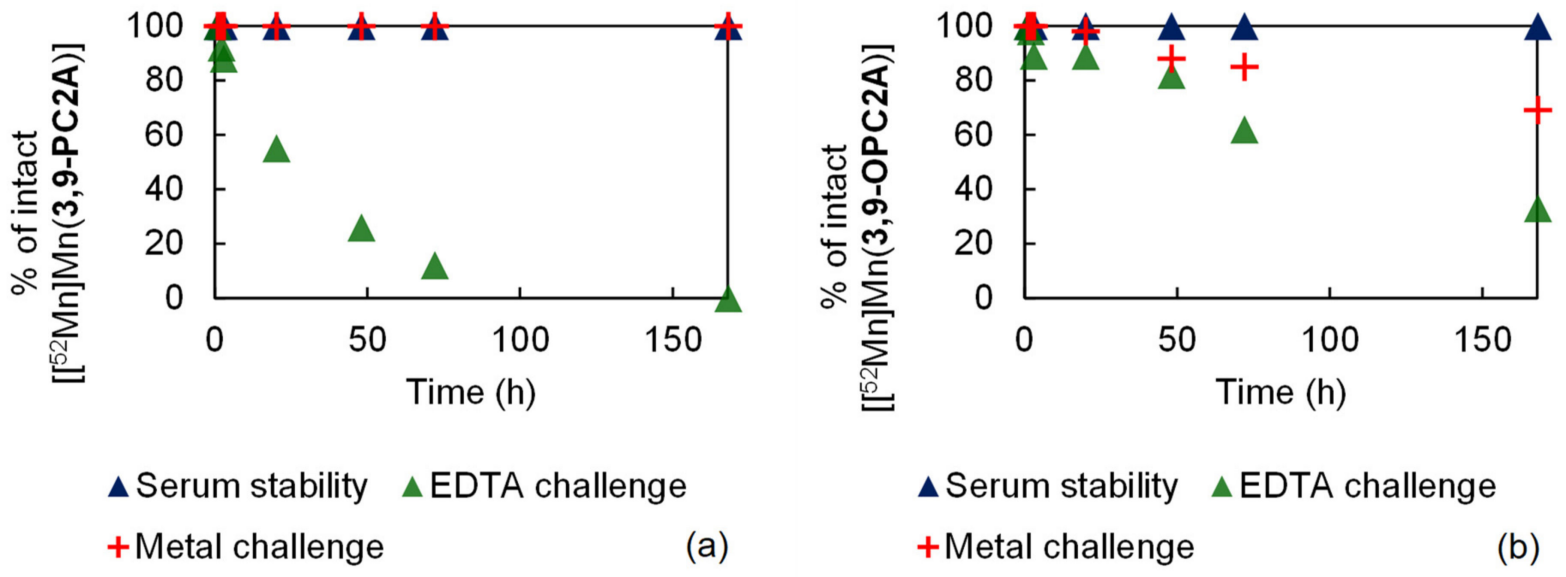
Results of the stability tests of [[^52^Mn]Mn(**3,9-PC2A**)] (**a**) and [[^52^M]Mn(**3,9-OPC2A**)] (**b**).

The inertness of the purified [[^52^Mn]Mn(**3,9-PC2A**)] and [[^52^Mn]Mn(**3,9-OPC2A**)] was evaluated with the serum stability test; transchelation by the EDTA ligand and transmetalation was provoked by a cocktail of essential metal ions (Zn(II), Cu(II), Mg(II), and Ca(II)). The results of these studies are presented in Figure 7. For the serum stability measurement, both radiolabeled complexes were incubated with rat serum at room temperature. According to the radio-TLC chromatograms, both ^52^Mn labeled complexes were stable during the in vitro test, since they remained intact even after seven days. For the H_4_**EDTA** challenge, the [[^52^Mn]Mn(**3,9-PC2A**)] and [[^52^Mn]Mn(**3,9-OPC2A**)] complexes were mixed with 0.2 M H_4_**EDTA** (pH 7.4) at room temperature, respectively. In the case of [[^52^Mn]Mn(**3,9-PC2A**)] the transchelation was complete, while, in the case of [[^52^Mn]Mn(**3,9-OPC2A**)], it was slower and was not completed after seven days. The inertness of both radiolabeled complexes against the dissociation provoked by the endogenous metal ions, was monitored by radio-TLC as a function of time. For the metal challenge the [[^52^Mn]Mn(**3,9-PC2A**)] and [[^52^Mn]Mn(**3,9-OPC2A**)] complexes were mixed with a 1:1 mixture of 0.1 mM ZnCl_2_ and with 0.01 mM CuCl_2_ (1 µL), and a 1:1 mixture of 1.02 mM MgCl_2_ and 2.28 mM CaCl_2_ (50 µL) at room temperature, respectively. During the metal challenge, the [[^52^Mn]Mn(**3,9-PC2A**)] complex stayed intact, while about 30 % of [[^52^Mn]Mn(**3,9-OPC2A**)] dissociated, during the course of the study (7 days).

Finally, the octanol/water partition coefficient (log*P*) of the complexes was determined, and was found to be −2.51 for [[^52^Mn]Mn(**3,9-PC2A**)] and −2.56 for [[^52^Mn]Mn(**3,9-OPC2A**)]. These log*P* values indicate that both radiolabeled compounds are hydrophilic, and there is no significant difference in the polarity of the [[^52^Mn]Mn(**3,9-PC2A**)] and [[^52^Mn]Mn(**3,9-OPC2A**)] complexes.

### 2.7. DFT Calculations

**Figure 8:**
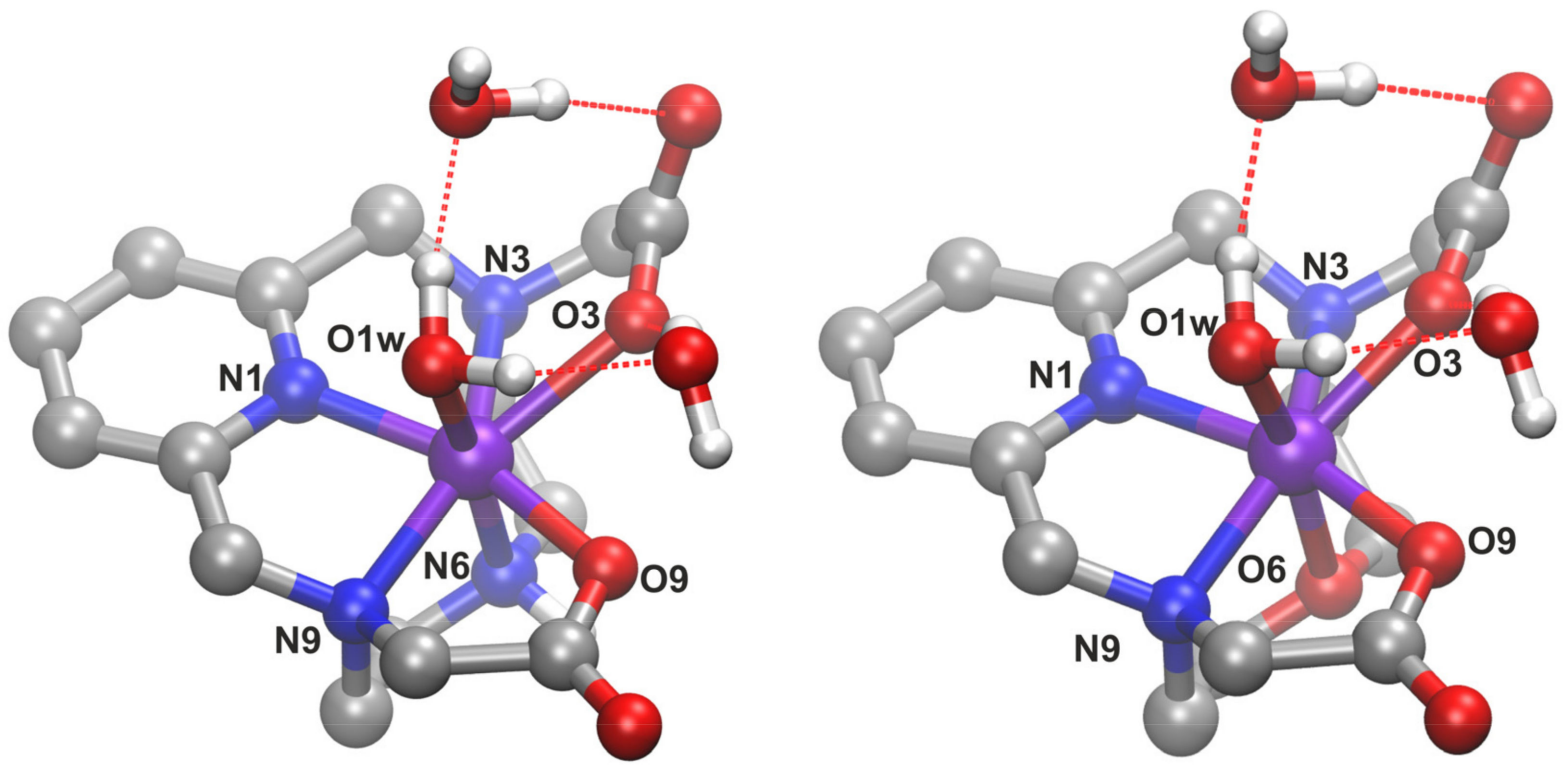
Ball-and-stick representation of the optimized structures of the [Mn(**3,9-PC2A**)] (**left**) and [Mn(**3,9-OPC2A**)] (**right**) complexes. Geometrical data on bonding can be found in Table 7 (O: red; N: blue; C: gray; H: white; and Mn: violet).

In order to understand the changes in the structure of the complex upon replacing the position 6 NH moiety of H_2_**3,9-PC2A** with an oxygen atom, we performed DFT calculations on the corresponding structures. The complexes were built based on an earlier study on analogous compounds [16]. To better account for the interaction with the solvent, we included two explicit second-sphere water molecules beyond implicit solvation [16,40]. Ball-and-stick images of the optimized complexes, H_2_**3,9-PC2A** and H_2_**3,9-OPC2A**, are shown in Figure 8, whereas their total energies and XYZ coordinates can be found in the supporting information (Table S1).

Interestingly, the obtained bond distances involving the manganese atom are significantly shorter within **3,9-PC2A**, than in our earlier report [16], for which the reason probably lies in the different functional used in the two sets of calculations. The shortest coordination within this structure is with the nitrogen atom of the pyridine ring, followed closely by the O9 atom of a carboxylate group (Table 7). The Mayer bond orders (MBO) [41,42] underscore the strength of these bonds. Similar to earlier observations [16], the bond between N3 and the metal atom seems to be rather weak. The lack of strong electron donation to the manganese, leaves the partial atomic charges of N3 significantly more negative, and the Mayer bonded valence (MBV) [41,42] lower than the chemically equivalent N9 (Table 8). According to the bonding descriptors and the partial atomic charges, a clear difference in the coordination between the two carboxylate groups can be observed, presumably due to the stronger interaction of the hydrogen bonding water molecules at the carboxylate moiety at position 3, which compete with the manganese atom for this group, thereby rendering its coordination weaker. The interaction of the negatively charged carboxylate groups with the metal center is not merely electrostatic, since the bonds exhibit a significant covalent character as well, as shown by the MBOs (Table 7). In the present context, the most important interplay within the complex is obviously the Mn–N6 bond, which is obviously affected the most by the substitution with an oxygen atom. According to both the corresponding bond length and MBO, the bonding between the metal and this site of the ligand significantly contributes to the coordination.

The overall geometry of the complex barely changes upon the introduction of the oxygen atom (Figure 8, Table 7), indicating similar structures within the two compounds. However, the weaker coordination of the O6 oxygen atom in H_2_**3,9-OPC2A**, compared to N6 in H_2_**3,9-PC2A**, is apparent; despite the smaller size of the oxygen atom, the Mn–O6 bond is longer than the Mn–N6, while the MBO value drops to almost its half. This weakened coordination is partly compensated by a somewhat stronger Mn–O3 bond. Nonetheless, the overall metal–ligand interaction is notably weaker in H_2_**3,9-OPC2A** than in H_2_**3,9-PC2A**, as shown by the partial atomic charge at the manganese atom that is more positive in [Mn(**3,9-OPC2A**)] (Table 8), indicating a less efficient electron donation from the ligand. This trend is especially conspicuous in the MBV and Mayer free valence data (MFV, Table 8), showing that the metal atom participates less in the bonding, and more of its valence is left free. Interestingly, despite these changes in coordination, the spin populations do not show any significant change at the metal center.

We also attempted to calculate the proton affinity of H_2_**3,9-PC2A** and H_2_**3,9-OPC2A**, to support the experimental findings regarding the decreased basicity upon the exchange of the position 6 nitrogen atom to an oxygen. However, during all the geometry optimizations of the protonated H_2_**3,9-OPC2A**, the proton spontaneously transferred from the oxygen atom at position 6 to one of the carboxylate moieties, resulting in a species with a different constitution. However, while the basicity of H_2_**3,9-OPC2A**, at position 6, cannot be assessed directly in a quantitative manner, the apparent lack of any relevant minima at the potential energy surface indicates that even the relatively weakly basic carboxylate groups have a higher affinity for the proton than the oxygen atom in question. Thus, the calculations show that―in accordance with the experiments―the position 6 oxygen atom is not basic, and the replacement of the nitrogen atom at that site effectively decreases the basicity of the ligand.

## 3. Summary/Conclusions

A new hexadentate ligand was designed to improve the kinetic properties of its complex formed with an Mn(II) ion. A pyclen derivative macrocycle, in which the nitrogen atom situated trans to the pyridine was replaced by an oxygen atom, was synthesized by following the Richman–Atkins macrocyclization protocol, while the ligand was obtained following the alkylation of the secondary nitrogen atoms with protected sidearms. The replacement of the nitrogen atom for an oxygen atom in the H_2_**3,9-OPC2A** ligand, resulted in a noticeable drop in ligand basicity, as well as the stability constants of the complexes formed with Mn(II) and the most abundant essential metal ions, such as Mg(II), Ca(II), Cu(II), and Zn(II). However, the conditional constants, such as the pMn values, remained unaffected. Dissociation kinetic studies indicated that [Mn(**3,9-OPC2A**)] had the lowest value of the rate constant (*k*_1_) characteristic for the proton-assisted dissociation among the complexes formed with structurally similar (pyclen and cyclen) diacetate systems. Moreover, the *k*_1_ value was considerably smaller than that of the [Mn(**1,4-DO2AM^Me2^**)]^2+^ complex that was previously designed to improve the kinetic inertness of the Mn(II) complexes. This improvement was the result of the absence of the most basic >NH group in the backbone of the H_2_**3,9-PC2A** ligand.

Labeling experiments performed with the positron emitter ^52^Mn isotope indicate that the H_2_**3,9-OPC2A** ligand can be labeled more efficiently at high temperatures than the nitrogen atom containing parent H_2_**3,9-PC2A** chelator, whereas, at low temperatures (37 °C), H_2_**3,9-PC2A** displays better labeling features. As far as their dissociation is concerned, both labeled chelates perform well in human serum (in line with the results of the studies performed in the Seronorm solutions in the mM concentration range), while transchelation reactions using a large excess of **EDTA** confirmed a better dissociation kinetic profile for [Mn(**3,9-OPC2A**)]. On the other hand, the transmetalation reaction provoked by a cocktail of essential metal ions occurs at a greater extent for the [Mn(**3,9-OPC2A**)] chelate.

[Mn(**3,9-OPC2A**)] possesses one metal bound water molecule that exchanges relatively fast with the bulk solvent, yet its exchange rate is nearly three times lower than that of the parent [Mn(**3,9-PC2A**)] chelate. As expected, [Mn(**3,9-PC2A**)] and [Mn(**3,9-OPC2A**)] have very similar relaxivities, indicating that relaxivity is governed by fast rotation of such a low molecular weight paramagnetic species. The DFT calculations performed on the Mn(II) complex of H_2_**3,9-OPC2A**, indicate that the geometry of the complex does not severely change upon the replacement in the ligand of a nitrogen atom for an oxygen atom. However, the calculated bond lengths indicate the weaker coordination of the oxygen atom in H_2_**3,9-OPC2A** than that of the nitrogen atom in H_2_**3,9-PC2A**. Altogether, H_2_**3,9-OPC2A** appears to be a promising ligand for Mn(II) chelation, both in the context of MRI and/or ^52^Mn PET applications.

## 4. Materials and Methods

### 4.1. General Methods

Starting commercial reagents/solvents that were purchased from Sigma-Aldrich (St. Louis, MO, USA) and the Tokyo Chemical Industry (Tokyo, Japan) were used without further purification, unless otherwise stated. Deionized Milli-Q water was used for the preparation of all aqueous solutions (equilibrium/kinetic/relaxometric studies). The ^1^H and ^13^C NMR spectra were recorded on a Bruker Avance DRX 360 MHz spectrometer (chemical shifts are given as δ values with reference to the deuterated solvents (CDCl_3_, D_2_O, CD_3_CN, and CD_3_OD) and the coupling constants are reported in Hz). Mass spectra were recorded on a maXis II UHR ESI-QTOF MS Bruker instrument, in the Laboratory of Instrumental Analysis, the Department of Inorganic and Analytical Chemistry of the University of Debrecen. Analytical HPLC using Waters Alliance 2690 HPLC (Waters Inc., Milford, MA, USA) system, equipped with a Luna C18(2) 150 mm × 4.6 mm, 100 Å, 3 μm column (Phenomenex Inc., Torrance, CA, USA), at 25 °C temperature detection at 260 nm with a flow rate of 1.00 mL/min was applied to follow the reactions (conversion, assignment of byproducts etc.) and control the purity of the products. Prior to its use in the analytical experiments, the final product was purified by preparative HPLC using the YL9100 HPLC system (Youngin Chromass, Anyang-si, Korea) equipped with a Luna C18(2) column (250 mm × 21.2 mm, 100 Å, 5 μm, Phenomenex Inc., Torrance, CA, USA). The solvent flow rate, in this case, was 25.0 mL/min using a gradient elution with eluent 5 mM TFA in water and MeCN (Table 9). For the purification of the other components, we used the CombiFlash^®^ EZ Prep compact flash preparative chromatography system (Teledyne Isco Inc., Lincoln, NE, USA) applied with a Redisep RF Gold^®^ silica gel disposable flash column (12 gramm, 60 Å). The purity of the product was >99.8%, as determined by reverse-phase HPLC with UV-VIS detection at 220 and 260 nm.

### 4.2. Synthesis and Characterization of New Compunds

Important synthons, such as 2,6-bis(chloromethyl)pyridine or bis(2-aminoethyl)ether, were synthesized following the published procedures [25,26], and their synthesis was included in the ESI.

***N*,*N*-(oxydiethane-2,1-diyl)bis(4-methylbenzenesulfonamide) (3):** bis-(2-aminoethyl)-ether (**2**, 4.00 g, 38.4 mmol, 1.0 equivalent) was dissolved in 50 mL water. The pH of the solution was adjusted to 12 by using solid NaOH (3.23 g, 80.7 mmol, 2.1 eq.). 4-toluenesulfonyl chloride (15.4 g, 80.7 mmol, 2.1 eq.) was dissolved in diethyl ether and added dropwise to the vigorously stirred aqueous solution of amine. After the evaporation of the ether, a yellowish precipitate formed, which was collected by the careful decantation of the remaining aqueous solution. The excess water was decantated form the precipitate and the solid was suspended with 40 mL of cold, freshly distilled water. The suspension was stirred for 2 h and the precipitate was filtrated. The white solid was washed with cold water and diethyl-ether, and dried under vacuum. In case it is required, the product can be recrystallized from hot ethanol. Yield: 14.10 g (89 %) as a white solid. ^1^H-NMR (CDCl_3_) δ (ppm): 7.74 (4H, d, *J* = 8.1 Hz, aromatics), 7.27 (4H, d, *J* = 8.1 Hz, aromatics), 5.51 (2H, s, -N*H*-), 3.32 (4H, t, *J* = 5.1 Hz, -C*H_2_*-), 3.05 (4H, t, *J* = 5.1 Hz, -C*H_2_*-), 2.40 (6H, s, -C*H_3_*); ^13^C-NMR (CDCl_3_) δ (ppm): 143.4, 137.1 (2 × 2C, *C_q_*, aromatics), 129.8, 127.2 (2×4C, aromatics), 69.2, 42.9 (2×2C, -*C*H_2_-), 21.6 (2C, -*C*H_3_); ESI-MS (m/z, positive mode): (M+Na)^+^_calc._: 435.1019, (M+Na)^+^_found_: 435.1021; and analytical HPLC injection: *t*_R_ = 13.21 min.

**3,9-Bis((4-methylphenyl)sulfonyl)-6-oxa-3,9,15-triazabicyclo(9.3.1)entadeca-1(15),11,13-triene (6):** molecular sieves were loaded into a dry, heated flask. The 2,6-bis(chloromethyl)pyridine (**5,** 1.28 g, 7.27 mmol, 1.0 eq.) was dissolved in 25 mL of dry acetonitrile and this solution was added, dropwise, to the heated reaction mixture containing the *N*,*N*-(oxydiethane-2,1-diyl)bis(4-methylbenzenesulfonamide) (**3,** 3.00 g, 7.27 mmol, 1.0 eq.) in dry CH_3_CN (10 mL) and K_2_CO_3_ (10.05 g, 72.7 mmol, 10 equivalent) The reaction was stirred at reflux temperature under argon atmosphere. After the completion of the reaction, as monitored by analytical HPLC, the precipitate was filtered and the solvent from the filtrate was removed under reduced pressure. The residue was purified by flash chromatography (dichloromethane/methanol were used as mobile phases). Alternatively, the product can be purified by crystalizing it from boiling ethanol. In this case, the crude product has to be dissolved in a small amount of chloroform (5 mL), and boiling ethanol needs to be added to the solution (25 mL) until it becomes cloudy. Small sized white crystals can be obtained by keeping the solution in the fridge at 4 ^°^C. Yield: 2.21 g (59 %) as a white solid powder. ^1^H-NMR (CD_3_CN) δ (ppm): 7.77 (4H, t, *J* = 8.2 Hz, aromatics), 7.65 (1H, t, *J* = 7.6 Hz, aromatics), 7.42 (4H, d, *J* = 8.2 Hz, aromatics), 7.20 (2H, d, *J* = 7.6 Hz, aromatics), 4.29 (4H, s, -C*H_2_*-), 3.51 (4H, t, *J* = 4.7 Hz, -C*H_2_*-), 3.16 (4H, t, *J* = 4.7 Hz, -C*H_2_*-), 2.42 (6H, s, -C*H_3_*); ^13^C-NMR (CD_3_CN) δ (ppm): 157.1, 144.7 (2×2C, *C_q_*, aromatics), 138.2 (1C, aromatics), 136.5 (2C, *C_q_*, aromatics), 130.7, 128.3 (2×4C, aromatics), 123.6 (2C, aromatics), 70.5, 56.1, 50.6 (3×2C, -*C*H_2_-), 21.5 (2C, -*C*H_3_); ESI-MS (m/z, positive mode): (M+H)^+^_calc._: 516.1621, (M+H)^+^_found_: 516.1622; and analytical HPLC injection: *t*_R_ = 11.27 min.

**6-Oxa-3,9,15-triazabicyclo(9.3.1)pentadeca-1(15),11,13-triene (7)**: the 3,9-bis((4-methylphenyl)sulfonyl)-6-oxa-3,9,15-triazabicyclo(9.3.1)pentadeca-1(15),11,13-triene (**6**, 0.50 g, 0.970 mmol, 1.0 eq.) was dissolved in cc. H_2_SO_4_ (2.00 mL). The reaction was performed in a microwave reactor (120 °C, 20 W, 5 min.), and then it was cooled down, first to room temperature and later it was cooled further to 0 °C, by using a water–ice cooling mixture. Cold ether was added to the mixture in small portions, by applying continuous cooling and mixing, which resulted in a precipitate formation. The solvent was decanted from the dihydrogen sulphate salt of the precipitated macrocycle, and the residue was dissolved in 20 mL of distilled water. The pH of the solution was adjusted to 13.2 by using solid NaOH, and the product was extracted 3 times using chloroform (20 mL). The combined organic phase was dried over MgSO_4_ and evaporated under vacuum. Yield: 0.19 g (95%) as an orange oil. ^1^H-NMR (CD_3_CN) δ (ppm): 7.57 (1H, t, *J* = 7.6 Hz, aromatics), 7.04 (2H, d, *J* = 7.6 Hz, aromatics), 3.83 (4H, s, -C*H_2_*-), 2.96 (4H, t, *J* = 4.7 Hz, -C*H_2_*-), 2.65 (4H, t, *J* = 4.7 Hz, -C*H_2_*-); ^13^C-NMR (CD_3_CN) δ (ppm): 161.2 (2C, *C_q_*, aromatics), 137.4 (1C, aromatics), 121.5 (2C, aromatics), 69.9, 54.4, 50.0 (3×2C, -*C*H_2_-); ESI-MS (m/z, positive mode): (M+H)^+^_calc._: 208.1444, (M+H)^+^_found_: 208.1443; and analytical HPLC injection: *t*_R_ = 2.77 min.

**3,9-Diethyl-6-oxa-3,9,15-triazabicyclo(9.3.1)pentadeca-1(15),11,13-triene-3,9-diacetic ester (9):** the mixture of 6-oxa-3,9,15-triazabicyclo(9.3.1)pentadeca-1(15),11,13-triene (**7**, 0.30 g, 1.45 mmol, 1.0 eq.) and dry K_2_CO_3_ (0.44 mg, 3.19 mmol, 2.2 eq.) was loaded into a multi-necked flask, and the mixture was suspended in 80 mL of dry acetonitrile. The reaction was stirred at reflux temperature under argon atmosphere. The solution of the ethyl bromoacetate (**8**, 0.36 mL, 3.19 mmol, 2.2 eq., 1.22 g/mL) in acetonitrile (5 mL) was added dropwise to the acetonitrile suspension of the macrocycle and K_2_CO_3_. After completion of the reaction, monitored by analytical HPLC, the precipitate was filtered. The solvent was evaporated under reduced pressure, and the residue was dissolved in distilled water. The obtained crude product was purified with HPLC (Luna 10u-Prep C18(2) 100A (*2*50 × 21.20 mm; 10 μm) column), and ACN:H_2_O/TFA was applied as eluent (ACN: acetonitrile and TFA: trifluoroacetic acid). TFA was present only in water, in 0.005 M concentration. The yield of the analytically pure product was 0.46 g (85 %), in a form of yellow oil. ^1^H-NMR (CD_3_CN) δ (ppm): 8.27 (1H, t, *J* = 7.9 Hz, aromatics), 7.61 (2H, d, *J* = 7.9 Hz, aromatics), 4.43 (4H, s, -C*H_2_*-), 4.14 (4H, q, *J* = 7.1 Hz, -C*H_2_*-), 3.72 (4H, s, -C*H_2_*-), 3.44 (4H, t, *J* = 4.5 Hz, -C*H_2_*-), 3.02 (4H, t, *J* = 4.5 Hz, -C*H_2_*-), 1.22 (6H, t, *J* = 7.1 Hz, -C*H_3_*); ^13^C-NMR (CD_3_CN) δ (ppm): 171.9 (2C, *C_q_*, C=O), 155.0 (2C, *C_q_*, aromatics), 145.7 (1C, aromatics), 124.1 (2C, aromatics), 68.8, 61.6, 57.9, 57.1, 56.0 (5 × 2C, -*C*H_2_-), 14.4 (2C, -*C*H_3_); ESI-MS (*m*/*z*, positive mode): (M+Na)^+^_calc._: 402.1999, (M+H)^+^_found_: 402.2000; and analytical HPLC injection: *t*_R_ = 7.86 min.

**6-Oxa-3,9,15-triazabicyclo(9.3.1)pentadeca-1(15),11,13-triene-3,9-diacetic acid (3,9-OPC2A) (10):** The 3,9-Diethyl-6-oxa-3,9,15-triazabicyclo[9.3.1]pentadeca-1(15),11,13-triene-3,9-diacetic ester (**9**, 0.15 g, 0.395 mmol, 1 eq.) was dissolved in distilled water (5.0 mL), and solid NaOH (47 mg, 1.19 mmol, 3 eq.) was added to the solution. The reaction was heated to 80 °C and kept at this temperature for an hour, followed by analytical HPLC. After 1 h, the solvent was removed under reduced pressure and the residue was purified by preparative HPLC. Luna 10u-Prep C18(2) 100A (*2*50* ×* 21.20 mm; 10 μm) column was used for the separation and ACN:H_2_O/TFA was applied as eluent (ACN: acetonitrile; TFA: trifluoroacetic acid). TFA was present only in water in 0.005 M concentration. Yield: 0.12 g (95 %) as a colorless oil. ^1^H-NMR (CD_3_OD) δ (ppm): 8.18 (1H, t, *J* = 7.9 Hz, aromatics), 7.61 (2H, d, *J* = 7.9 Hz, aromatics), 4.75 (4H, s, -C*H_2_*-), 4.12 (4H, s, -C*H_2_*-), 3.46 (4H, t, *J* = 4.5 Hz, -C*H_2_*-), 3.43 (4H, t, *J* = 4.5 Hz, -C*H_2_*-); ^13^C-NMR (CD_3_OD) δ (ppm): 170.4 (2C, *C_q_*, C=O), 152.9 (2C, *C_q_*, aromatics), 142.8 (1C, aromatics), 123.6 (2C, aromatics), 66.7, 59.7, 58.4, 57.4 (4 × 2C, -*C*H_2_-); ESI-MS (*m*/*z*, positive mode): (M+H)^+^_calc._: 324.1554, (M+H)^+^_found_: 324.1557; and analytical HPLC injection: *t*_R_ = 2.84 min.

### 4.3. Equilibrium Studies

Metal stock solutions were prepared from the highest analytical grade chemicals, and their concentrations were determined by complexometric titration with standardized Na_2_H_2_**EDTA** and eriochrome black T indicator in the presence of ascorbic acid, and potassium hydrogen tartrate for MnCl_2_, murexide indicator for CaCl_2_ and CuCl_2_, eriochrome black T for MgCl_2_ and xylenol orange in the presence of hexamethylenetetramine for the solution of ZnCl_2_. The concentration of the ligand stock solution was determined by pH-potentiometric titrations. The protonation constants of the ligand were determined by the pH potentiometric titrations with 0.15 M NaOH, using 0.0025 M ligand solutions. The ionic strength was set to 0.15 M by using NaCl. The titrated samples (starting volume of 6 mL) were stirred mechanically, and thermostated at 25 °C by a circulating water bath (±0.1 °C). The protonation constants of the ligand (log *K*_i_^H^) are defined as follows:

(3) $$K_{i}^{H}=\frac{\left[H_{i}L\right]}{\left[H_{i-1}L\right]\left[H^{+}\right]}$$

where *i* = 1, 2, …, 4 and (H*_i_*_−1_L) and (H^+^) are the equilibrium concentrations of the ligand (*i* = 1), its protonated forms (*i* = 2, …, 4), and hydrogen ion, respectively. The stability constants of the complexes are defined as follows:

(4) $$K_{\mathrm{ML}}=\frac{\left[\mathrm{ML}\right]}{\left[M][L\right]}$$

(5) $$K_{\mathrm{ML}}^{H}=\frac{\left[\mathrm{MHL}\right]}{[\mathrm{ML}]\left[H^{+}\right]}$$

(6) $$K_{\mathrm{ML}}^{\mathrm{OH}}=\frac{[M(\mathrm{OH})L]\left[H^{+}\right]}{\left[\mathrm{ML}\right]}$$

To avoid the effect of CO_2_, N_2_ gas was bubbled through the solutions during the titrations process. The pH-potentiometric titrations were performed with a Metrohm 785 DMP Titrino titration workstation, with the use of a Metrohm 6.0234.100 combined electrode in the pH range of 1.75–11.8. For the calibration of the pH meter, KH-phtalate (pH = 4.005) and borax (pH = 9.177) buffers were used, and the H^+^ ion concentrations were calculated from the measured pH values by applying the method proposed by Irving et al. [43]. A solution of approximately 0.01 M HCl was titrated with a 0.15 M NaOH solution (0.15 or 1.00 M NaCl), and the differences between the measured and calculated pH values (for the points with pH < 2.4) were used to calculate the (H^+^) from the pH values measured in the titration experiments. The measured points with pH > 11.0 of the acid-base titration were used to calculate the ionic product of the water, which was found to be 13.845 (0.15 M NaCl) and 13.825 (1.0 M NaCl) under our experimental conditions. For the calculation of the equilibrium constants, the PSEQUAD program was used [44]. The stability constants of the metal complexes (except for Cu(II)) were determined using the direct pH-potentiometric method, by titrating samples with 1:1 metal-to-ligand ratios (the number of fitted data pairs were between 127–212), allowing 1 min for the sample equilibration to occur. The stability constants of the Cu(II) complex were too high to be determined by pH-potentiometry; hence, a direct UV-VIS spectrophotometric method was used. The spectrophotometric measurements were performed with a JASCO V-760 UV-VIS spectrophotometer (JASCO International Co., Ltd., Tokyo, Japan) at 25 °C, using semimicro 1.0 cm cells. For the determination of the stability constant, the absorbance was measured at 8 different acid concentrations (falling in the acid concentration range of 0.11–1.00 M), at 17 wavelengths between 640 and 800 nm (the concentration of the complex was 3.04 mM). The molar absorption coefficient of the Cu(II) and that of the [Cu(**3,9-OPC2A**)] were determined in a separate experiment, while that of the protonated [CuH(**3,9-OPC2A**)]^+^ chelate was obtained by the UV-VIS titartion [Cu(**3,9-OPC2A**)] of the complex (Figure S22). A pH-potentiometric titration of the Cu(II) complex was also performed in the pH range 1.75–11.85, with a starting volume of 6 mL, c_Cu(II)_ = c_lig_ = 2.5 mM, and these data were treated simultaneously with those obtained from the UV-VIS spectrophotometric measurements (Figures S21 and S22).

### 4.4. Relaxation Properties

The ^1^H longitudinal (*T*_1_) and transverse (*T*_2_) relaxation times were measured by using Bruker Minispec MQ-20 and MQ-60 NMR analyzers (Bruker corporation, Billerica, MA, USA). The temperature of the sample holder was set at 25.0 or 37 (±0.2) °C, and controlled with a circulating water bath thermostat. The *T*_1_ values of the samples were determined by means of the inversion recovery method (180°–τ–90°), averaging 4–6 data points obtained at 12 different τ delay times, while *T*_2_ data were collected by using the Carl–Purcell–Meiboom–Gill (CPMG) spin-echo pulse sequence. Relaxivities were determined by published procedures (from the slopes of the plots of 1/*T*_1,2_ vs. (Mn(II)) for 4 concentrations of Mn(II)), in samples of 0.3–0.4 mL volume [45,46] (data shown in Figures S24 and S25, Supplementary Information). The pH were set by either using a 0.05 M 4-(2-hydroxyethyl)-1-piperazine-ethanesulfonic acid (HEPES) buffer (pH = 7.4) or monitored (pH profiles) using a Metrohm 827 pH lab pH meter and a Metrohm 6.0234.100 combined electrode, in the pH range of 1.75–11.8.

### 4.5. Kinetic Studies

The inertness of the [Mn(**3,9-OPC2A**)] complex was studied by various methods. The transmetalation reaction initiated by the Zn(II) ions was monitored by measuring the *T*_2_ relaxation times as a function of time at pH = 6.0, set by a 50 mM 2-(N-morpholino)ethanesulfonic acid (MES) buffer in the presence of 25 equivalents of Zn(II) ions at 37 °C. These conditions were recently used by P. Caravan et al., and employed in the present study to obtain data that can be directly compared with that of [Mn(**PyC3A**)]^–^ [6].

Metal exchange reactions occurring between the complex and Cu(II) (in the presence of a 20- and 40-fold excess, to ensure pseudo-first-order conditions) was investigated at 25 °C and 0.15 M NaCl ionic strength, by using a JASCO V-760 UV-VIS spectrophotometer at 290 nm. The concentration of the complexes were set to 0.27 mM for the Mn(II) complex of H_2_**3,9-OPC2A**, while the concentration of CuCl_2_ was as follows: 5.73 and 11.26 mM. All the kinetic studies were performed by using a noncoordinating buffer, N,N’-dimethyl piperazine (DMP, log *K*_2_^H^ = 4.19) at 0.05 M concentration to maintain a constant pH in the samples, in the pH range of 3.46–4.97. The values of the pseudo-first-order rate constants (*k*_obs_) were determined using the following equation: A_t_ = A_v_ + (A_0_ − A_v_)e^kobs×t^, where A_t_ is the absorbance at time t, A_0_ is the absorbance of the reactants, and A_v_ is the absorbance of the products.

The serum stability of the Mn(II) complexes was studied by following the *r*_1p_ relaxation rates of the complex over time, using 1.0 mM solutions in SeronormTM (Seronorm = commercially available lyophilized human blood serum, Sero, Stasjonsveien, Norway) at pH = 7.43 and 25 °C.

### 4.6. H- and ^17^O-NMR Relaxometry

A Bruker Avance 400 (9.4 T) spectrometer was used to measure the longitudinal (1/*T*_1_) and transverse (1/*T*_2_) relaxation rates, and the chemical shifts of an aqueous solution of the [Mn(**3,9*-*OPC2A**)] complex (pH = 7.4, 1.3 mM) and a diamagnetic reference (HClO_4_ acidified water, pH = 3.3) in the temperature range of 273–348 K. The temperature calibration was performed using literature protocols relying on ethylene glycol and methanol standards [47]. The 1/*T*_1_ and 1/*T*_2_ values were determined by the inversion−recovery and the Carr−Purcell−Meiboom−Gill spin echo technique, respectively [48]. To avoid susceptibility corrections of the chemical shifts, a glass sphere fitted into a 10 mm NMR tube was used to contain the samples. ^17^O enriched water (10% H_2_^17^O, CortecNet) was added to the solutions to improve sensitivity (circa 1% enrichment). Proton NMRD profiles of the [Mn(**3,9*-*OPC2A**)] complex (1.00 mM, pH = 7.4) were recorded in aqueous solution on a Stelar SMARTracer Fast Field Cycling relaxometer (0.01−10 MHz), and a Bruker WP80 NMR electromagnet adapted to variable field measurements (20−80 MHz) and controlled by a SMARTracer PC-NMR console (Stelar s.r.l., Mede, Italy). The temperature was monitored by a VTC91 temperature control unit and maintained by a gas flow. The temperature was determined by previous calibration with a Pt resistance temperature probe. The least-squares fit of the ^17^O NMR and the NMRD data, was performed using a Visualiseur/Optimiseur [49] running on a MATLAB 8.3.0 (R2014a) platform.

### 4.7. Computational Details

DFT calculations were performed by using the Orca program with the TPSSh functional [50,51], and the def2-TZVPP basis set [52], using the D3 correction for dispersion interactions with Becke–Johnson damping [53,54], with a grid setting of 5. This functional has been noted for its good performance regarding Mn(II) complexes [55]. To account for the solvent effects from the aqueous medium, the CPCM implicit solvation model was used [56]. The geometry of each structure was fully optimized with the tight settings of Orca, followed by a subsequent frequency calculation to make sure that the obtained stationary point is a minimum on the potential energy surface.

### 4.8. Radiochemistry

**General.** All solvents and reagents that were used for the radiochemical experiments possessed high purity, to avoid metallic contamination. Ultrapure (u.p.) water and u.p. HCl were purchased form Carl Roth and absolute ethanol form Merck. The Cr pieces were obtained from Alfa Aesar. The ^52^Mn radioisotope was prepared in GE PETtrace cyclotron at the Division of Nuclear Medicine and Translational Imaging, Department of Medical Imaging, University of Debrecen, Hungary. Activity measurements were carried out with a CAPINTEC CRC-15PET dose calibrator and a Perkin Elmer Packard Cobra gamma counter. Radio-TLC was performed on TLC Silica gel 60 (form Merck) and analyzed using a MiniGita TLC-Scanner with GINA-Star TLC software. AG^®^ 1-X8 resin was purchased from Bio-Rad Laboratories. The purification of the labeled compounds was carried out with a Waters SepPak^®^ C18 plus cartridge.

**Production and purification of ^52^Mn isotope.** The ^52^Mn isotope was produced by proton irradiation with a 20 MeV beam on a natural Cr target (99.99%, 0.1–0.31 in, ~250 mg) via a ^52^Cr(p, n)^52^Mn reaction [57]. A 60 min irradiation with a 20 μA beam current yielded an approx. 150 MBq ^52^Mn isotope. The purification of the radionuclide was achieved with the previously described method, used developed by Fonslet et al. [58], which was based on the solid phase extraction of manganese from acid/organic solvent mixtures, reported earlier by Graves et al. [59]. After a 24 h decay period, the irradiated Cr target was dissolved in 2 mL of concentrated HCl. The solution was concentrated to ~1.5 mL and solid CrCl_3_ was observed. The solution was separated from the precipitation and diluted to 50 mL with absolute ethanol, and transferred onto an AG^®^ 1-X8 extraction column (300 mg), which was preconditioned with 3 *v*/*v*% concentrated HCl in absolute ethanol (5 mL). The column was washed with 20 mL of 3 *v*/*v*% concentrated HCl in absolute ethanol, and the ^52^Mn isotope was eluted with 0.5 mL of 0.1 M HCl. The ^52^Mn solution was concentrated to dryness and redissolved in 300 µL of concentrated HCl, and then diluted to 10 mL with absolute ethanol. The process was repeated 2 more times, but, for the second time, 200 mg, and for the third time, 100 mg of AG^®^ 1-X8 resin was used for the preparation of the extraction column. Finally the ^52^Mn isotope (~100 MBq) was eluted with 400 µL of 0.1 M HCl and used for radiolabeling experiments.

**^52^Mn radiolabeling of** H_2_**3,9-PC2A.** A volume of 200 µL of 1 M HEPES buffer (pH = 7) and 50 µL of H_2_**3,9-PC2A** (1 mg/mL aqueous solution, 0.155 µmol) were added to 250 µL of ^52^Mn in 0.1 M HCl (~30 MBq) solution. The reaction mixture was heated at 95 °C for 10 min, and was then passed through a SPE cartridge (SepPak^®^ C18 Plus, Waters) preconditioned with 5 mL of ethanol and 5 mL of water. After the purging of the cartridge with 1 mL of water, the labeled complex was eluted with 400 µL of 33 *v*/*v*% ethanol. This solution was concentrated to dryness and the [[^52^Mn]Mn(**3,9-PC2A**)] was redissolved in 400 µL of water and used for stability studies. The radiochemical purity of the [[^52^Mn]Mn(**3,9-PC2A**)] was determined by radio-TLC using TLC Silica gel 60 plate developed with a 2:1 mixture of water and acetonitrile as the mobile phase (Figure S28).

**^52^Mn radiolabeling of** H_2_**3,9-OPC2A.** The ^52^Mn radiolabeling of H_2_**3,9-OPC2A** and the purification of the labeled complex was performed in a similar manner as described in the case of [[^52^Mn]Mn(**3,9-PC2A**)]. 200 µL of 1 M HEPES buffer (pH = 7) and 50 µL of H_2_**3,9-OPC2A** (1 mg/mL aqueous solution, 0.154 µmol) were added to ^52^Mn in 0.1 M HCl (~30 MBq, 250 µL). After heating at 95 °C for 10 min, the reaction mixture was purified using SepPak^®^ C18 Plus cartridge, as described above. The radiochemical purity of [[^52^Mn]Mn(**3,9-OPC2A**)] was determined by the radio-TLC method, as mentioned before (Figure S28).

The investigation of ^52^Mn labeling efficiency of H_2_**3,9-PC2A** and H_2_**3,9-OPC2A** chelators using different ligand concentrations (10, 25, 30, 50, 75, 100, and 150 µM) at 95 and 37 °C. 1 M HEPES buffer (pH = 7, 20 µL), and 5 µL of H_2_**3,9-OPC2A** or H_2_**3,9-PC2A** aqueous solution with different concentrations, were added to 25 µL ^52^Mn in 0.1 M HCl (~3 MBq). The applied ligand concentrations in the mixtures were as follows: 100 µM, 75 µM, 50 µM, 25 µM, and 10 µM for H_2_**3,9-PC2A**, and 150 µM; 100 µM; 75 µM; 50 µM; and 25 µM for H_2_**3,9-OPC2A**, respectively. The reaction mixtures were heated at 95 and 37 °C, respectively. Then, these mixtures were analyzed by the above-mentioned radio-TLC method at different time points of 5, 10, 15, and 20 min.

**Stability test of [[^52^Mn]Mn(3,9-PC2A)] and [[^52^Mn]Mn(3,9-OPC2A)] in rat serum.** 100 µL aqueous solution of each radiolabeled complex (~7.5 MBq) was mixed with 100 uL of rat serum, and incubated at room temperature. Aliquots were taken and analyzed at the beginning and after 1, 2, 3, and 20 h, and 2, 3, and 7 days later, by the radio-TLC method, as explained mentioned above.

**EDTA challenge of [[^52^Mn]Mn(3,9-PC2A)] and [[^52^Mn]Mn(3,9-OPC2A)].** 50 µL aqueous solution of each radiolabeled complex (~3.75 MBq) was incubated with 50 µL of 0.2 M **EDTA** (pH 7.4) solution at room temperature. Aliquots were taken and analyzed by the radio-TLC method, as mentioned above, at the beginning and after 1, 2, 3, and 20 h, and 2, 3, and 7 days incubation time.

**Metal challenge of [[^52^Mn]Mn(3,9-PC2A)] and [[^52^Mn]Mn(3,9-OPC2A)].** 49 µL aqueous solution of each radiolabeled complex (~3.75 MBq) was incubated with a 1:1 mixture of 0.1 mM ZnCl_2_ and 0.01 mM CuCl_2_ (1 µL), and a 1:1 mixture of 1.02 mM MgCl_2_ and 2.28 mM CaCl_2_ (50 µL) at room temperature. Samples were taken from the mixtures at the beginning and after 1, 2, 3, and 20 h, and 2, 3, and 7 days later. For the analysis of the samples, the above-mentioned radio-TLC method was applied.

**Determination of logP values of the [[^52^Mn]Mn(3,9-PC2A)] and [[^52^Mn]Mn(3,9-OPC2A)] chelates.** 100 µL aqueous solution of each radiolabeled complex (~7.5 MBq) was added to 100 µL of octanol. The mixture was shaken with a vortex shaker (5 min, 1000 rpm) and centrifuged (5 min, 10,000 rpm). A total of 3 × 20 µL from the octanol and 3 × 20 µL from the water were pipetted into test tube, respectively. The radioactivity of the samples was measured with a gamma detector.

## Figures and Tables

**Table 1:**
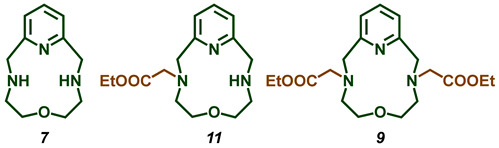
The ratio of the formed compounds during the alkylation of the O-pyclen macrocycle with different amounts of ethyl-2-bromoacetate (information about the applied method of analytical HPLC are in the ESI).

Equivalent Amount of Compound 8	O-pyclen (7) *t*_R_ = 2.77 min	3-OPCA^OEt^ (11) *t*_R_ = 4.74 min	3,9-OPC2A^OEt^ (9) *t*_R_ = 7.86 min
0.4	52.1%	47.9%	-
0.8	17.5%	66.7%	15.8%
1.2	-	54.1%	45.9%
1.6	-	16.2%	83.8%
2.0	-	1.9%	98.1%

**Table 2:** Protonation constants of the H_2_**3,9-OPC2A,** H_2_**3,9-PC2A,** H_2_**3,6-PC2A,** H_2_**1,4-DO2A,** H_2_**1,7-DO2A**, and H_2_**1,7-O2DO2A** ligands (T = 25 °C, *I* = 0.15 M NaCl; the values shown in parenthesis are the standard deviations).

	H_2_**3,9-OPC2A**	H_2_**3,9-PC2A ^a^**	H_2_**3,6-PC2A ^a^**	H_2_**1,4-DO2A ^b^**	H_2_**1,7-DO2A ^b^**	H_2_**1,7-O2DO2A ^c^**
log *K*_1_^H^	7.73(2)	12.25	10.72	11.44	11.69	8.05
log *K*_2_^H^	7.66(1)	5.97	8.37	9.51	9.75	7.43
log *K*_3_^H^	2.13(1)	3.47	3.81	4.14	3.97	2.06
log *K*_4_^H^	-	1.99	1.26	1.55	2.68	-
Σlog *Κ*_2_^H^	15.39	18.22	19.09	20.95	21.44	15.48

^a^ Ref. [16]; ^b^ Ref. [12]; and ^c^ Ref. [20].

**Table 3:** Stability constants (log*K*_ML_), protonation constants (log*K*_ML_^Hi^), and pMn values for the metal complexes formed with H_2_**3,9-OPC2A**, H_2_**3,9-PC2A**, H_2_**3,6-PC2A**, H_2_**1,4-DO2A**, H_2_**1,7-DO2A**, and H_2_**1,7-O2DO2A** ligands (T = 25 °C, *I* = 0.15 M NaCl; the values shown in parenthesis are the standard deviations).

		H_2_**3,9-OPC2A**	H_2_**3,9-PC2A ^a^**	H_2_**3,6-PC2A ^a^**	H_2_**1,4-DO2A ^b^**	H_2_**1,7-DO2A ^b^**	H_2_**1,7-O2DO2A ^c^**
	Σlog *Κ*_2_^H^	15.39	18.22	19.09	20.95	21.44	15.48
Mg^2+^	log *K*_MnL_	7.02(1)	9.84;(8.4) ^d^	8.11	-	-	3.91
	log *K*_MnHL_	-	5.91	5.87	-	-	-
Ca^2+^	log *K*_MnL_	8.27(1)	9.92;(10.0) ^d^	9.57	8.62	8.86	6.96
	log *K*_MnHL_	-	5.08	5.27	-	-	-
Mn^2+^	log *K*_MnL_	13.03(1)	17.09	15.53	15.68	14.64	9.38
	log *K*_MnHL_	2.40(2)	2.14	3.06	4.15	4.40	-
	log *K*_MnLOH_	11.49(1)	-	-	-	-	12.38
	pMn ^e^	8.69	8.64	8.09	7.27	6.52	6.67
Zn^2+^	log *K*_MnL_	14.81(1)	19.49	20.37	18.03	18.86	10.55
	log *K*_MnHL_	2.08(3)	2.74	2.36	3.58	4.23	-
	log *K*_MnLOH_	10.95(2)	-	-	1.65 ^f^	1.78 ^f^	11.40
Cu^2+^	log *K*_MnL_	18.41(4) ^g^	23.58 ^g^	24.09 ^g^	24.43 ^g^	24.24	14.56
	log *K*_MnHL_	1.92(4) ^g^	2.12 ^g^	2.37 ^g^	2.95	3.06	2.46
	log *K*_MnLOH_	10.50(5)	-	-	-	-	11.95

^a ^Ref. [16]; ^b^ Ref. [12]; ^c^ Ref. [20]; ^d^ Ref. [30]; ^e^ Ref. [11]; ^f^ determined by the simultaneous fitting of pH-pot. and UV-Vis spectrophotometric data; and ^g^ the values shown are the second protonation constants of the complexes (log *K*_M(HL)*H_).

**Table 4:** The relaxivities (mM^−1^s^−1^) of the Mn(II) complexes formed with H_2_**3,9-OPC2A**, H_2_**3,9-PC2A**, H_2_**3,6-PC2A**, H_2_**1,4-DO2A**, H_2_**1,7-DO2A**, and H_2_**1,7-O2DO2A** ligands at 25 °C and pH = 7.4 (the values shown in parenthesis are the standard deviations).

		H_2_**3,9-OPC2A** (25 °C/37 °C)	H_2_**3,9-PC2A ^a^**	H_2_**3,6-PC2A ^a^**	H_2_**1,4-DO2A ^b^**	H_2_**1,7-DO2A ^b^**	H_2_**1,7-O2DO2A ^c^**
0.49 T	*r*_1p_	3.13(2)/2.54(1)	2.91	2.72	2.10	1.50	2.86
	*r*_2p_	5.15(3)/4.17(4)	3.96	3.49	-	-	-
1.41 T	*r*_1p_	2.72(2)/2.06(1)	2.24	2.40	-	-	-
	*r*_2p_	9.90(5)/7.37(3)	4.82	4.20	-	-	-

^a^ Ref. [16]; ^b^ Ref. [12]; and ^c^ Ref. [20].

**Table 5:** Relaxivity and best-fit parameters obtained from the analysis of ^17^O NMR and NMRD data for the (Mn(**3,9-OPC2A**)) complex, compared with the corresponding values of [Mn(**3,9-PC2A**)], [Mn(**1,4-DO2A**)], Mn(**1,4-DO2AM^Me2^**]^2+^, and [Mn(**1,7-O2DO2A**)].

**Parameter**	[(Mn(**3,9-OPC2A**)]	[Mn(**3,9-PC2A**)] ^a^	[Mn(**1,4-DO2A**)] ^b^	[Mn(**1,4-DO2AM**^Me2^)]^2+ c^	[Mn(**1,7-O2DO2A**)] ^d^
r_1_^298^_20 MHz_/mM^−1^s^−1^	3.09	2.91	2.1	2.5	2.86
k_ex_^298^/10^7^s^−1^	5.3 ± 0.4	12.6	113	11.5	5.3
ΔH^‡^/kJmol^−1^	28.5 ± 1.7	37.5	29.4	36.9	29.7
ΔS^‡^/JK^−1^mol^−1^	−1.9 ± 0.8	–	–	-	2.8
τ_R_^298^/ps	40.0 ± 1.1	–	46	53	46.7
E_R_/kJmol^−1^	14.8 ± 0.6	–	19.1	19.1	20 ^e^
Δ^2^/10^18^s^−2^	17.8 ± 3.6	–	481	510	111
τ_v_^298^/ps	19.3 ± 3.0	–	4.4	5.5	–
q ^e^	1	1	0.87	0.87	1

^a^ Ref. [16]; ^b^ Ref. [21]; ^c^ Ref. [13]; ^d^ Ref. [20]; ^e^ fixed during the fitting procedure.

**Table 6:** Rate and equilibrium constants and half-lives at 7.4, for the dissociation reactions of [Mn(**3,9-OPC2A**)], [Mn(**3,9-PC2A**)], [Mn(**3,6-PC2A**)], [Mn(**1,4-DO2A**)], Mn(**1,4-DO2AM^Me2^**)]^2+^, and [Mn(**1,7-O2DO2A**)] complexes (T = 25 °C, *I* = 0.15 M NaCl).

	[Mn(**3,9-OPC2A**)]	[Mn(**3,9-PC2A**)] ^a^	[Mn(**3,6-PC2A**)] ^a^	[Mn(**1,4-DO2A**)] ^b^	[Mn(**1,4-DO2AM**^Me2^)]^2+ c^	[Mn(**1,7-O2DO2A**)] ^d^
*k*_0_ (s^−1^)	(8.6 ± 1.1) × 10^−6^	– ^e^	– ^e^	– ^e^	– ^e^	– ^e^
*k*_1_ (M^−1^s^−1^)	2.81 ± 0.07	221	70	99	8.7	85
*k*_2_ (M^−2^s^−1^)	-	-	1.5 × 10^5^	1.4 × 10^6^	-	3.0 × 10^6^
*k*_3_ (M^−1^s^−1^)	-	3.6 × 10^−2^	2.6 × 10^−2^	-	-	-
*K*_MnL x H_	-	3.6 × 10^3^	1.15 × 10^3^	-	-	-
*K*_ML x Cu_	7 ± 4	26	16	-	-	-
*t*_1/2_ (h) at pH = 7.4	21.9 ^f^ 1625	21.0	63.2	48	556	56.8

^a^ Ref. [16]; ^b^ Ref. [12]; ^c^ Ref. [13]; ^d^ Ref. [20]; ^e^ fixed to 0 during the data fitting; and ^f^ calculated with the use of *k*_0_ value.

**Table 7:** Bonding within the complexes [Mn(**3,9-PC2A**)] and [Mn(**3,9-OPC2A**)], characterized through selected bond lengths (R, in Å) and Mayer bond orders (MBO, in a.u.).

	[Mn(**3,9-PC2A**)]		[Mn(**3,9-OPC2A**)]	
Bond	R	MBO	R	MBO
Mn–N1	2.041	0.56	2.041	0.56
Mn–N3	2.610	0.24	2.692	0.22
Mn–N6/O6	2.077	0.48	2.190	0.26
Mn–N9	2.101	0.48	2.067	0.50
Mn–O3	2.252	0.19	2.099	0.26
Mn–O9	2.051	0.45	2.016	0.44
Mn–O1w	2.114	0.35	2.151	0.34

**Table 8:** Mulliken partial atomic charges (q, in a.u.), Mayer bonding valence (MBV, in a.u.), and Mayer free valence (MFV, in a.u.) of relevant atoms within the complexes [Mn(**3,9-PC2A**)] and [Mn(**3,9-OPC2A**)].

	[Mn(**3,9-PC2A**)]			[Mn(**3,9-OPC2A**)]		
atom	q	MBV	MFV	q	MBV	MFV
Mn	0.297	3.46	1.50	0.434	3.19	1.77
N1	−0.118	3.15	0.00	−0.108	3.15	0.00
N3	−0.238	2.97	0.00	−0.254	2.97	0.00
N6/O6	−0.019	3.22	0.00	−0.277	2.07	0.00
N9	−0.086	3.24	0.00	−0.081	3.31	0.00
O3	−0.520	1.85	0.00	−0.516	1.87	0.00
O9	−0.464	1.89	0.00	−0.456	1.92	0.00
O1w	−0.447	2.08	0.00	−0.455	2.05	0.00

**Table 9:** Applied gradient flow of analytical HPLC (column: Luna C18(2) 150 mm × 4.6 mm, 100 Å, 3 μm).

	Solvent A (MeCN)	Solvent B (Aqueous 5 mM TFA Solution)
0.0 min	0%	100%
15.0 min	90%	10%
16.0 min	0%	100%

## Data Availability

All supplementary materials can be found at MDPI. Our data will also be made available to any investigator upon request.

## Supplementary Materials

Analytical (^1^H- and ^13^C-NMR, ESI-MS and HPLC) data of the synthesized compounds (Figures S1–S20), electronic spectra obtained for the [Cu(3,9-OPC2A)] complex as a function of c_H+_ (Figures S21 and S22), analytical HPLC chromatogram of [Mn(**3,9-OPC2A**)] (Figure S23), determination of the *r*_1p_ and *r*_2p_ relaxivity at 0.49 and 1.41 T field strength at 25 and 37 °C (Figure S24 and S25), behavior of the complex in the presence of large Zn(II) excess and in Seronorm (Figures S26 and S27), radio-TLC chromatograms of the purified [[^52^Mn]Mn(**3,9-PC2A**)] and [[^52^Mn]Mn(3,9-OPC2A)] (Figure S28) and XYZ coordinates (in Å) and total electronic energies (in Hartree) of [Mn(**3,9-OPC2A**)] and [Mn(**3,9-PC2A**)] complexes as well as Mulliken spin populations for relevant atoms calculated by DFT methods (Tables S1 and S2) are included in the Supporting Information. Structures of the ligands mentioned in the text (Scheme S1).

## Sample Availability

Limited amounts of the compounds **3,9-OPC2A** and **3,9-OPC2A** are available from the corresponding author.

## List of Abbreviations

MRI	Magnetic Resonance Imaging
PET	Positron Emission Tomography
CAs	Contrast Agents
GBCAs	Gadolinium-Based Contrast Agents
NSF	Nephrogenic Systemic Fibrosis
BFCs	Bifunctional Chelators
EMA	European Medicines Agency
FDA	US Food and Drug Administration
HSA	Human Serum Albumin
DFT	Density Functional Theory
KPhth	Potassium Phthalimide
TsCl	4-Methylbenzene-1-sulfonyl Chloride
DMF	*N*,*N*-Dimethylformamide
DIPEA	*N*,*N*-Diisopropylethylamine
TEA	Triethylamine
TFA	Trifluoroacetic Acid
HEPES	*N*-(2-Hydroxyethyl)piperazine-*N*′-(2-Ethanesulfonic Acid)
mAb	Monoclonal Antibody
MBO	Mayer Bond Orders
MBV	Mayer Bonded Valence
MFV	Mayer Free Valence
