# Supplementary material for: A New Oxygen Containing Pyclen-Type Ligand as a Manganese(II) Binder for MRI and 52Mn PET Applications: Equilibrium, Kinetic, Relaxometric, Structural and Radiochemical Studies
Source: Molecules. 2022 Jan 7;27(2):371. doi: 10.3390/molecules27020371 (PMC8778187; doi:10.3390/molecules27020371)
Supplement: Supplementary file 1 [file molecules-27-00371-s001.zip › molecules-1485105-supplementary.pdf]

Supplementary Material

A New Oxygen Containing Pyclen-Type Ligand as a Manganese(II) Binder for MRI and ^{52}Mn PET Applications: Equilib-rium, Kinetic, Relaxometric, Structural and Radiochemical Studies

Tibor Csupász,[1,2] Dániel Szücs,[1,2,3] Ferenc K. Kálmán,[1] Oldamúr Hollóczki,[1,4] Anikó Fekete,[3] Dezső Szikra,[3] Éva Tóth,[5] Imre Tóth[1] and Gyula Tircsó*[1]

---

[1] *Department of Physical Chemistry, Faculty of Science and Technology, University of Debrecen, H-4032 Debrecen, Egyetem tér 1., Hungary*

[2] *Doctoral School of Chemistry, University of Debrecen, H-4032 Debrecen, Egyetem tér 1., Hungary*

[3]*Department of Medical Imaging, Division of Nuclear Medicine, Faculty of Medicine, University of Debrecen, H-4032 Debrecen, Egyetem tér 1., Hungary*

[4] *Mulliken Center for Theoretical Chemistry, University of Bonn, Beringstr. 4+6, D-53115 Bonn, Germany*

[5] *Centre de Biophysique Moléculaire, CNRS-UPR 4301, Université d'Orléans, Rue Charles Sadron, CS 80054, 45071 Orléans, France*

Corresponding author: gyula.tircso@science.unideb.hu

Table of content

Scheme S1. Structures of the ligands mentioned in the text. 2

S1. Synthesis of starting materials 2

S2. ^{1}H-NMR, ^{13}C-NMR, MS spectra and analytical HPLC chromatograms of produced compounds (Figure S1 – S20) 4

Figure S21. Absorption spectra of the Cu(II)–(**3,9-OPC2A**)–H^+ system 14

Figure S22. Changes observed in the absorption spectra of the [Cu(**OPC2A**)] complex as a function of H^+ ion concentration 14

Figure S23. Analytical HPLC chromatogram of **[**Mn**(3,9-OPC2A)]** 15

Figure S24. Determination of r_{1p} relaxivity for [Mn(**3,9-OPC2A**)] 15

Figure S25. Determination of r_{2p} relaxivity for [Mn(**3,9-OPC2A**)] 15

S3. Measurements of ^{17}O NMR relaxation rates 16

Figure S26. The $1/T_2$ values as a function of time for the dissociation of [Mn(**3,9-OPC2A**)] and [Mn(**3,9-PC2A**)] complexes 19

Figure S27. Stability investigation of the [Mn(**3,9-OPC2A**)] complex in Seronorm solution 19

Table S1. XYZ coordinates (in Å) and total electronic energies (in Hartree) of [Mn(**3,9-OPC2A**)] and [Mn(**3,9-PC2A**)] complexes 19

Table S2. Mulliken spin populations for relevant atoms. 23

Figure S28. Radio-TLC chromatograms of the purified [[^{52}Mn]Mn(**3,9-PC2A**)] and [[^{52}Mn]Mn(**3,9-OPC2A**)] 23

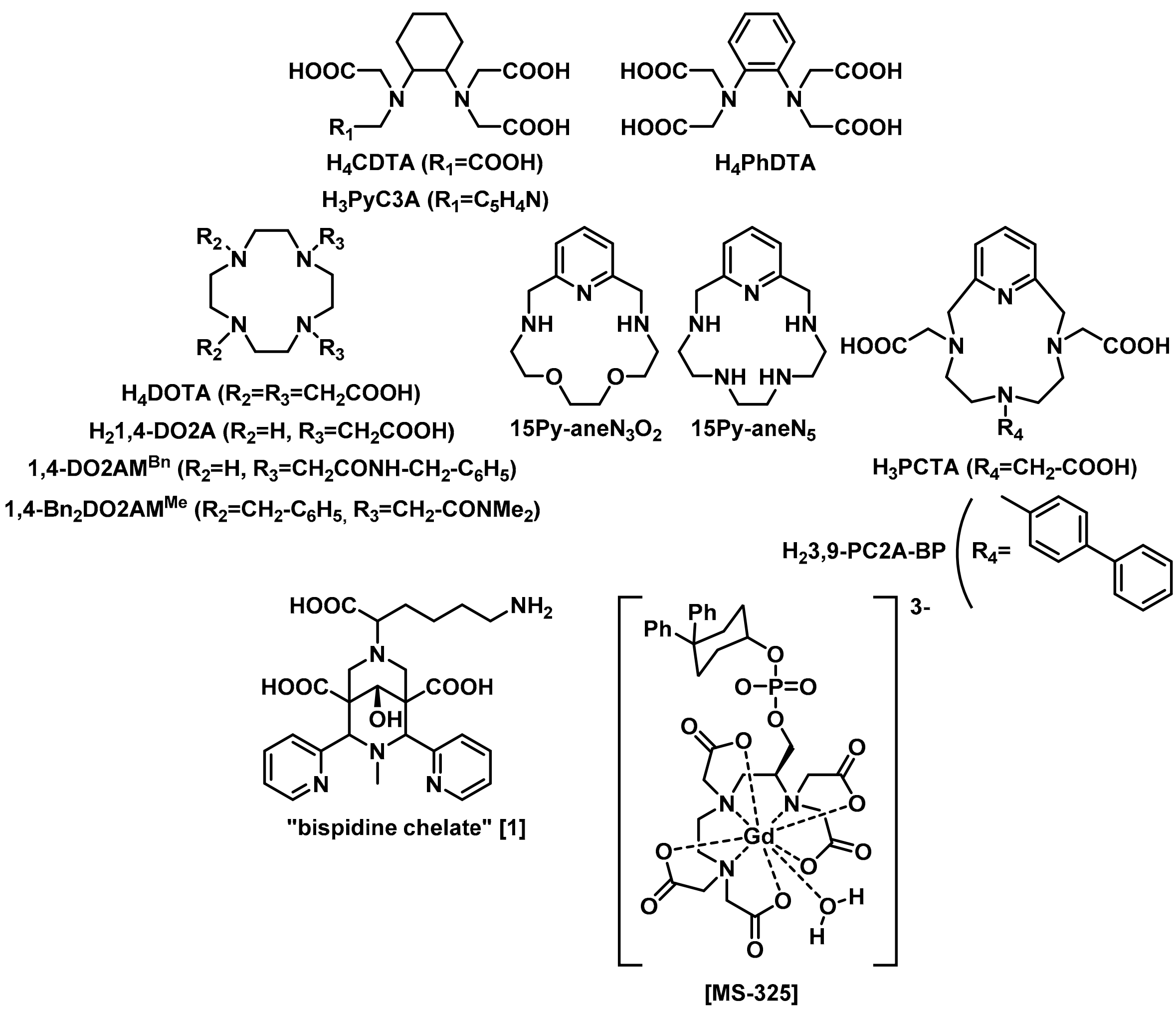

Scheme S1. Structures of the ligands mentioned in the text.

S1. Synthesis of starting materials

2,6-Bis(chloromethyl)pyridine [2]**:** Commercially available 2,6-pyridinedimethanol (10.0 g, 71.9 mmol, 1 eq.) was measured into the flask, which was cooled to 0 °C. Thionyl chloride (60 mL) was added dropwise to the compound over 10 minutes and the mixture stirred vigorously. After the addition of the reagent, the reaction mixture was stirred for an additional 5 hours. The mixture was cooled down to 4 °C in the fridge, and then it was poured into 100 mL cooled diethyl ether. The precipitated white solid was filtered and washed with cold diethyl ether. The dry precipitate was dissolved in 120 ml water and the solution was neutralized with solid NaOH. Near to the neutral pH a white solid appear in the solution. This mixture was cooled in fridge overnight the precipitate formed meanwhile was collected via vacuum filtration and washed with small amount, cold water and was dried under vacuum. Yield: 9.80 g (77 %) as a white solid. ^{1}H-NMR (CD_3CN) δ (ppm): 7.83 (1H, t, *J*=7.8 Hz, aromatic), 7.46 (2H, d, *J*=7.8 Hz, aromatics), 4.68 (4H, s, -C*H*$_2$-); ^{13}C-NMR (CD_3CN) δ (ppm): 157.6 (*C*$_q$, aromatics), 139.6,

123.5 (aromatics), 47.5 (-*C*H$_2$-); ESI-MS (m/z, positive mode): $[M+H]^+_{calc.}$: 176.0028, $[M+H]^+_{found}$: 176.0029.

Bis(2-aminoethyl)ether: The bis(2-aminoethyl)ether was synthesized following the literature procedure.[3] Bis(2-chloroethyl)ether (10.0 ml, 85.3 mmol, 1 eq., 1.22 g/mL) was dissolved in DMF (200 mL) and phthalimide potassium salt (34.8 g, 188 mmol, 2.2 eq.) was added to the solution. The reaction was heated to 60 °C stirred over 24 hours at the given temperature. The hot reaction mixture was filtered off and the precipitate was washed with 100 mL hot DMF. The filtrate was evaporated under reduced pressure. The residue was dissolved in 300 mL chloroform and hydrazine hydrate (33.0 mL, 853 mmol, 10 eq., 80 w/w%, 1.03 g/mL) was added to the mixture, which was stirred at reflux. During the reaction, the white by-product formed precipitated off from the heated reaction mixture. After 5 hours the reaction mixture was cooled down and kept in the fridge and the by-product formed was filtered off and the precipitate was washed with 100 mL cold chloroform. The product was obtained from the filtrate after removing solvent under reduced pressure . Yield: 6.98 g (79 %) as an orange oil. ^{1}H-NMR (D$_2$O) δ (ppm): 3.55 (4H, t, *J*=5.5 Hz, -C*H*$_2$-), 2.78 (4H, t, *J*=5.5 Hz, -C*H*$_2$-); ^{13}C-NMR (D$_2$O) δ (ppm): 72.0, 40.0 (-*C*H$_2$-); ESI-MS (m/z, positive mode): $[M+Na]^+_{calc.}$: 127.0842, $[M+Na]^+_{found}$: 127.0846.

S2. ^{1}H-NMR, ^{13}C-NMR, MS spectra and analytical HPLC chromatograms of produced compounds (Figure S1 – S20)

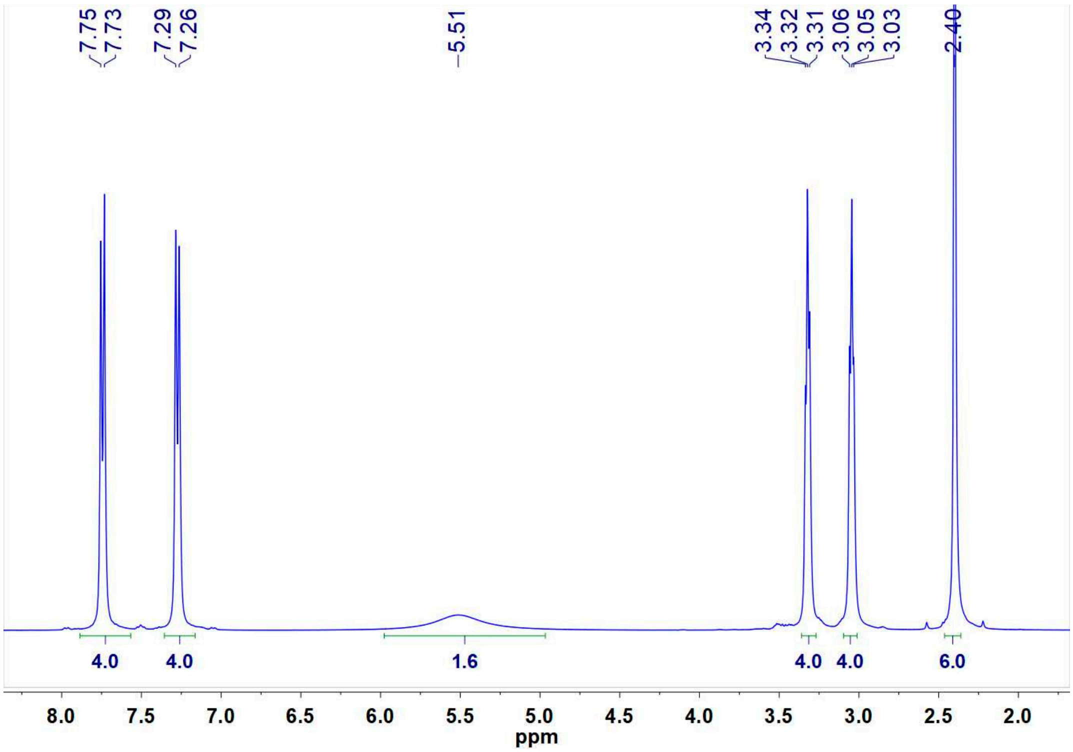

Figure S1. ^{1}H-NMR spectrum of ***N,N*-(oxydiethane-2,1-diyl)bis(4-methylbenzenesulfonamide) (3)** (Reference: $CDCl_3$).

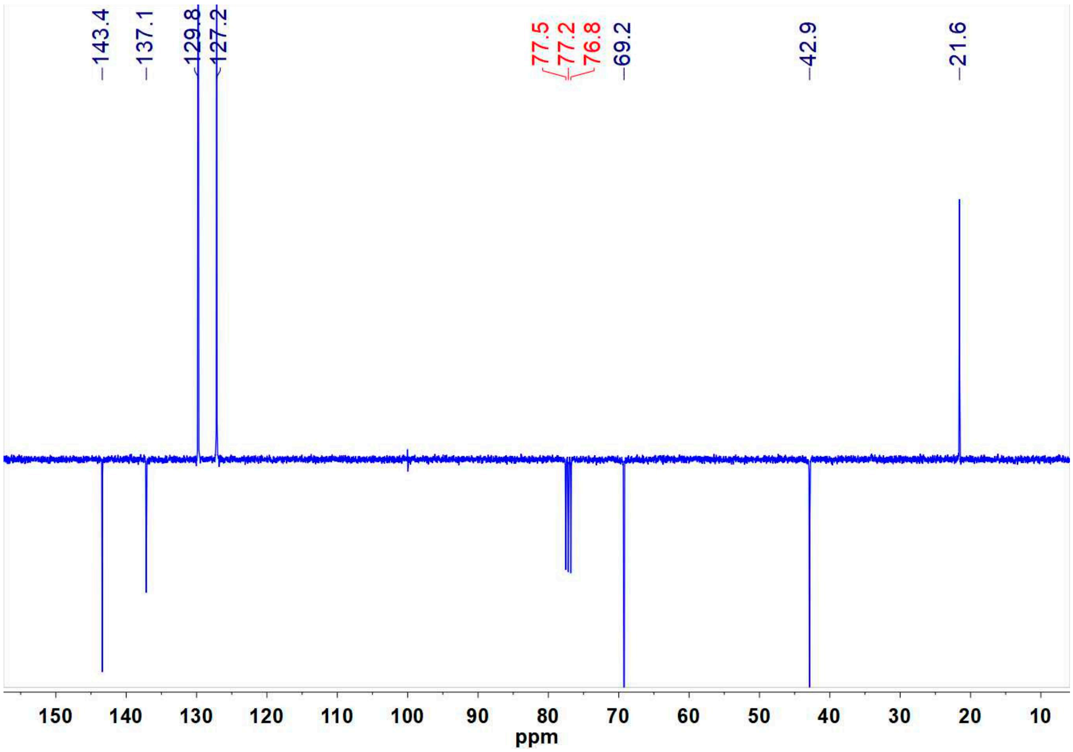

Figure S2. ^{13}C-NMR spectrum of ***N,N*-(oxydiethane-2,1-diyl)bis(4-methylbenzenesulfonamide) (3)** (Reference: $CDCl_3$).

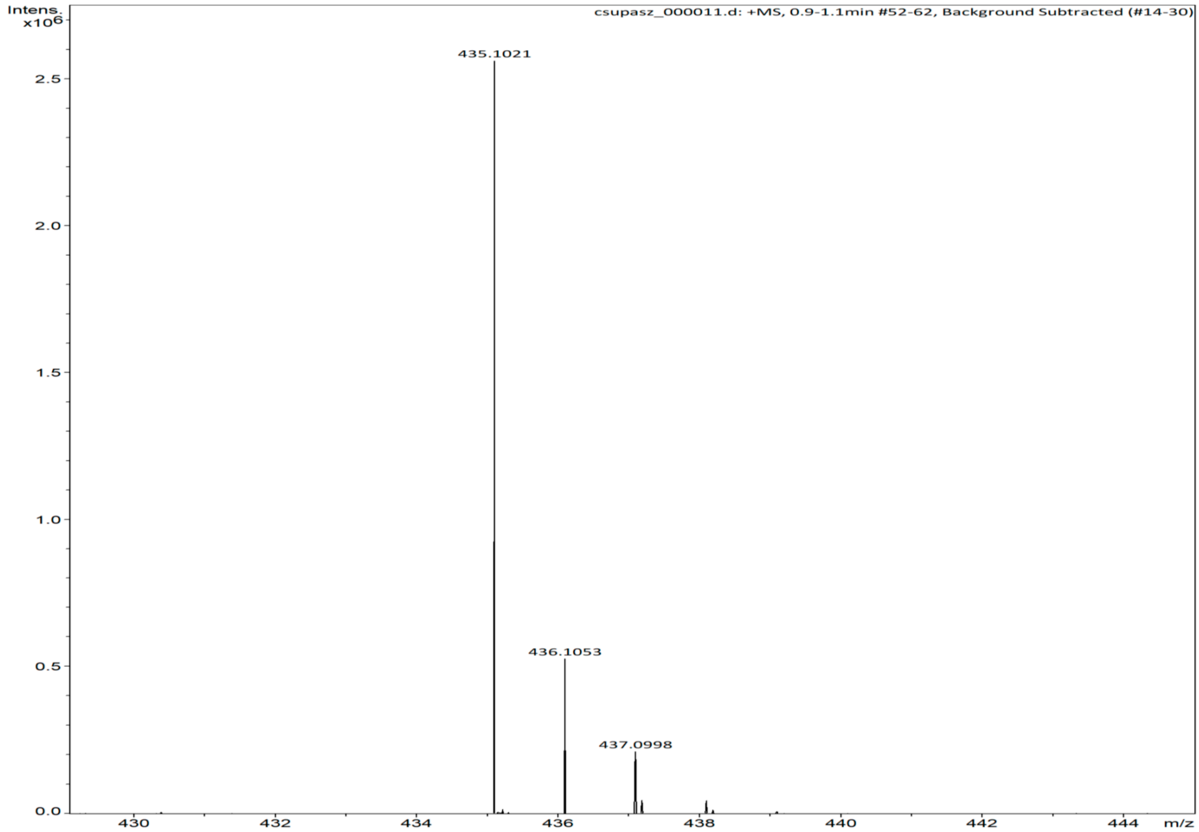

Figure S3. MS spectrum of ***N,N*-(oxydiethane-2,1-diyl)bis(4-methylbenzenesulfonamide) (3)**.

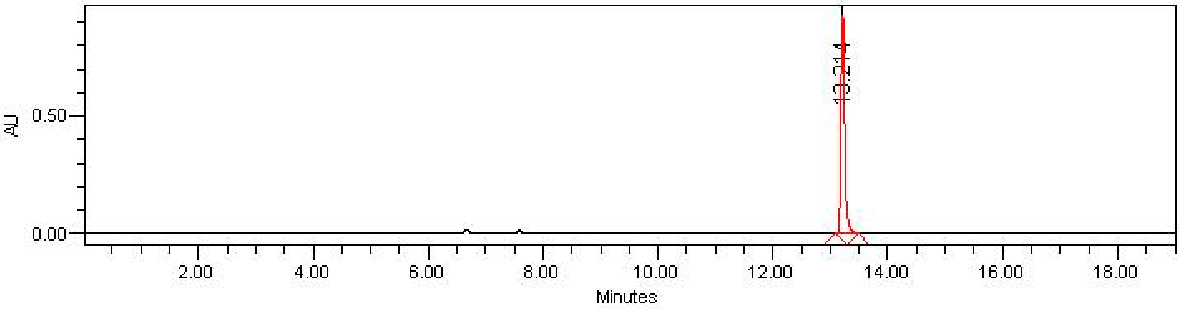

Figure S4. Analytical HPLC chromatogram of ***N,N*-(oxydiethane-2,1-diyl)bis(4-methylbenzenesulfonamide) (3)** (t_R=13.21 min).

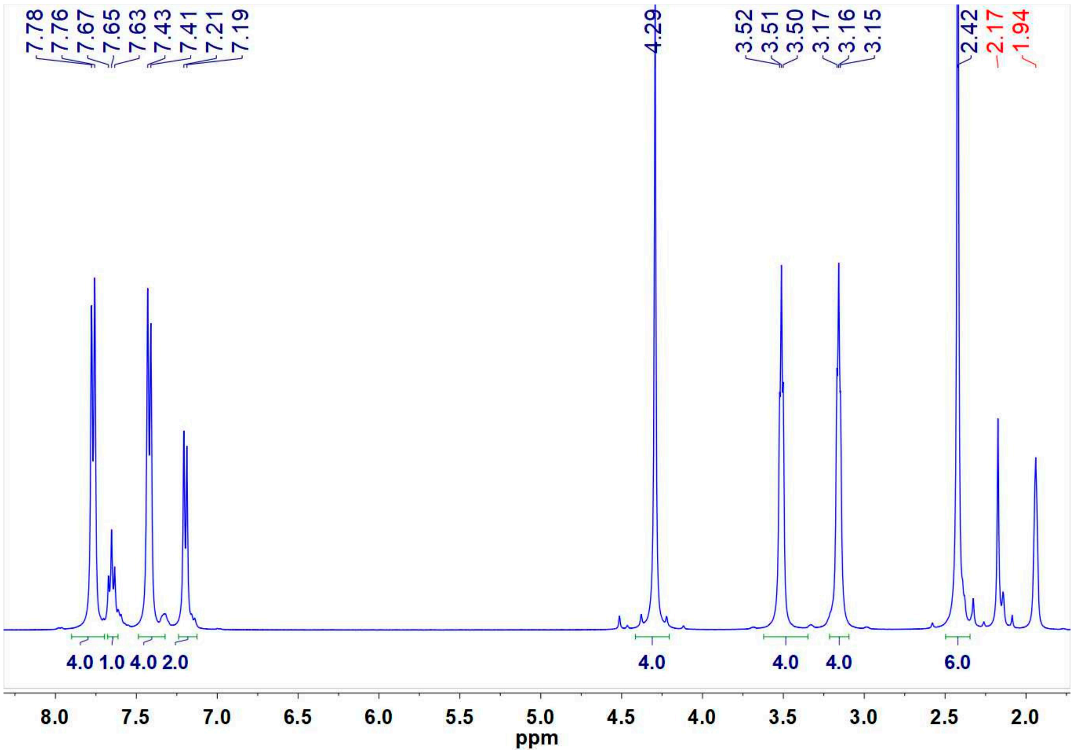

Figure S5. ^{1}H-NMR spectrum of **3,9-bis[(4-methylphenyl)sulfonyl]-6-oxa-3,9,15-triazabicyclo[9.3.1]pentadeca-1(15),11,13-triene (6)** (Reference: CD_3CN).

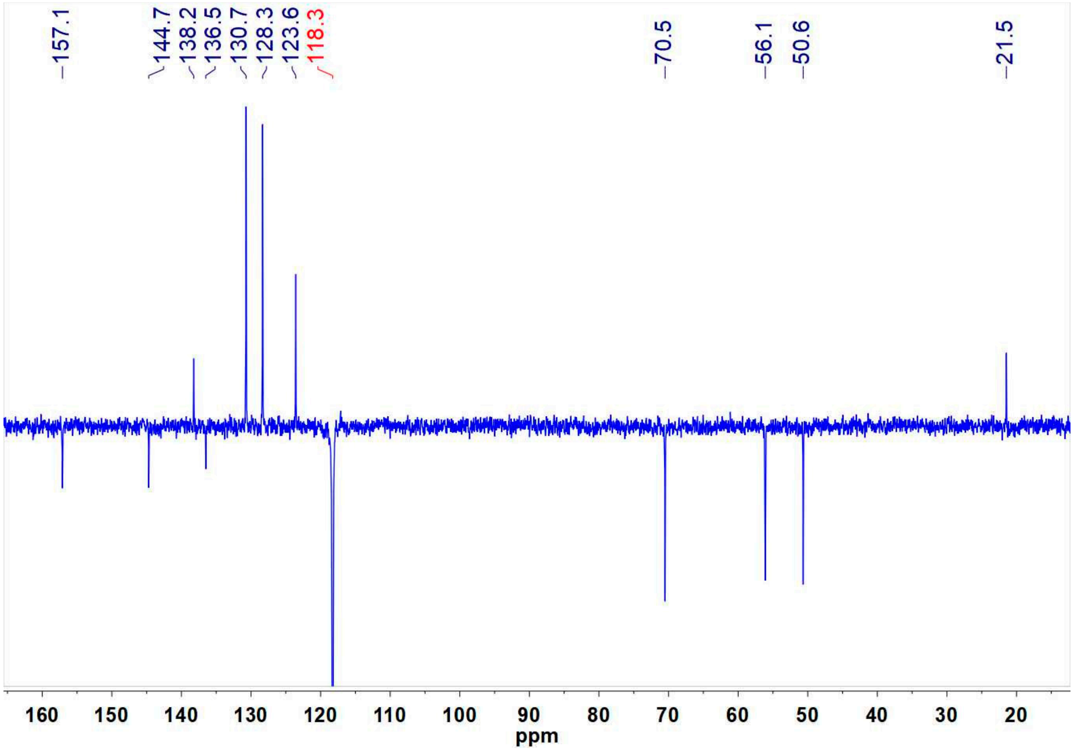

Figure S6. ^{13}C-NMR spectrum of **3,9-bis[(4-methylphenyl)sulfonyl]-6-oxa-3,9,15-triazabicyclo[9.3.1]pentadeca-1(15),11,13-triene (6)** (Reference: CD_3CN).

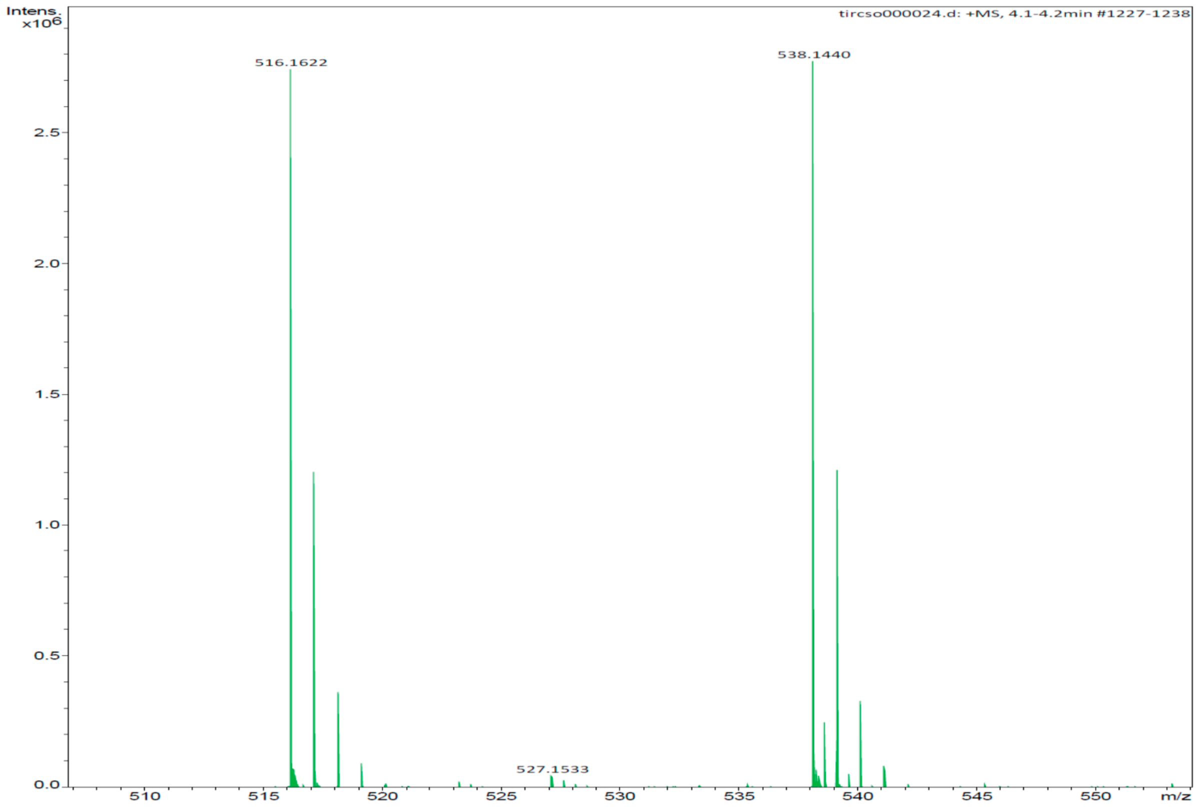

Figure S7. MS spectrum of **3,9-bis[(4-methylphenyl)sulfonyl]-6-oxa-3,9,15-triazabicyclo[9.3.1]pentadeca-1(15),11,13-triene (6)**.

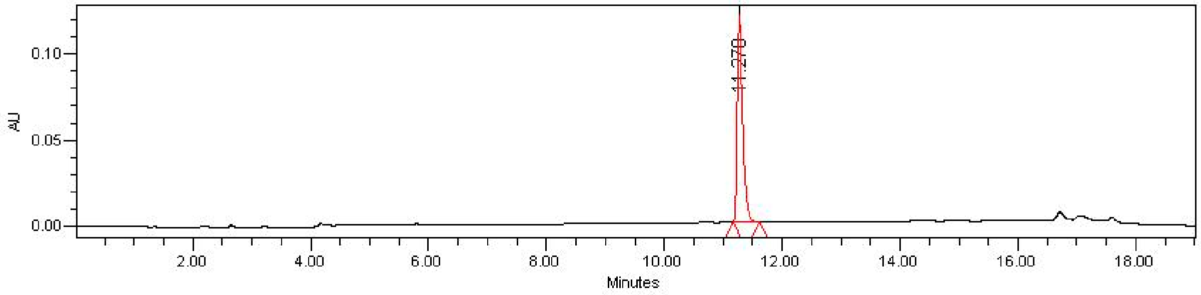

Figure S8. Analytical HPLC chromatogram of **3,9-bis[(4-methylphenyl)sulfonyl]-6-oxa-3,9,15-triazabicyclo[9.3.1]pentadeca-1(15),11,13-triene (6)** (t_R=11.27 min).

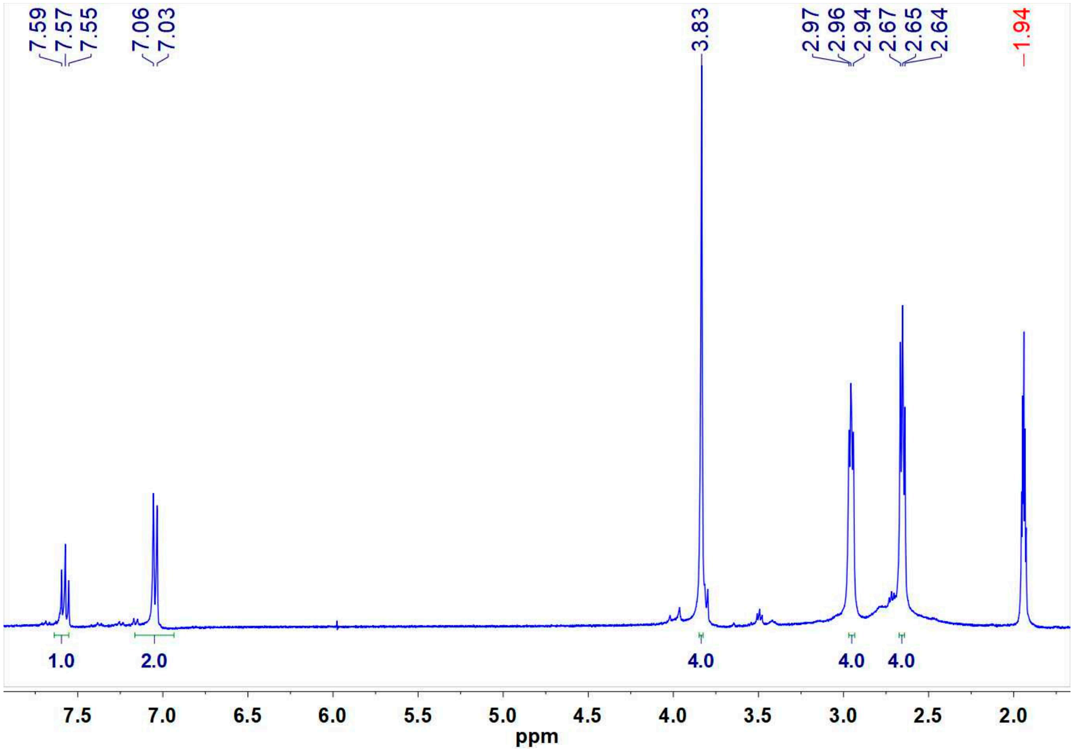

Figure S9. ^{1}H-NMR spectrum of **6-oxa-3,9,15-triazabicyclo[9.3.1]pentadeca-1(15),11,13-triene (7)** (Reference: CD_3CN).

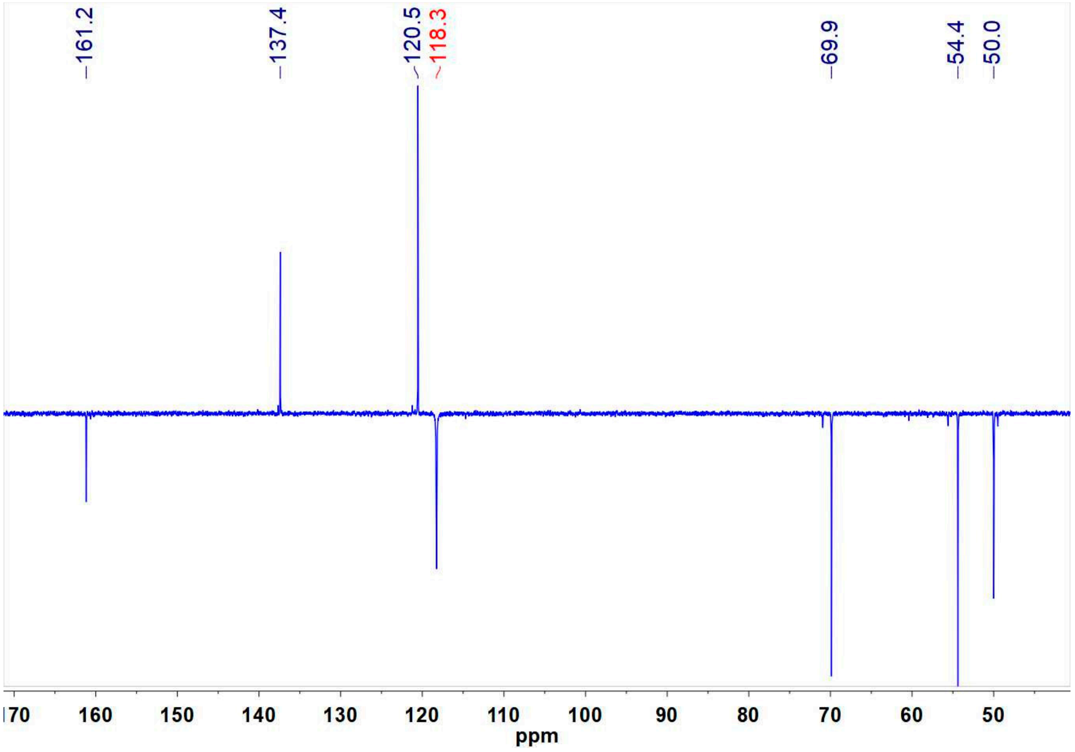

Figure S10. ^{13}C-NMR spectrum of **6-oxa-3,9,15-triazabicyclo[9.3.1]pentadeca-1(15),11,13-triene (7)** (Reference: CD_3CN).

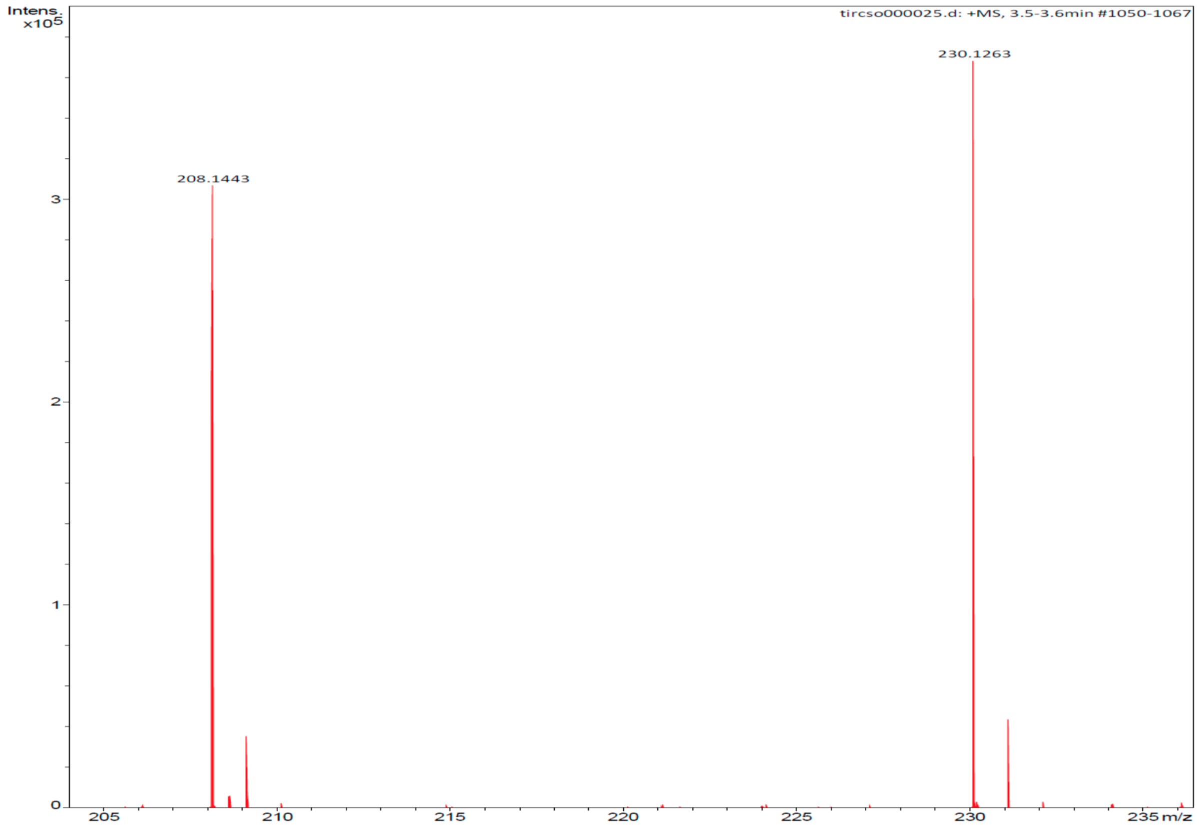

Figure S11. MS spectrum of **6-oxa-3,9,15-triazabicyclo[9.3.1]pentadeca-1(15),11,13-triene (7)**.

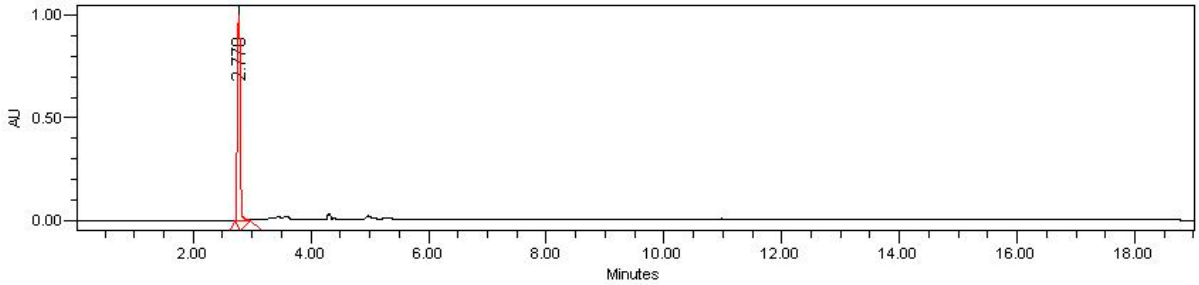

Figure S12. Analytical HPLC chromatogram of **6-oxa-3,9,15-triazabicyclo[9.3.1]pentadeca-1(15),11,13-triene (7)** (t_R=2.77 min).

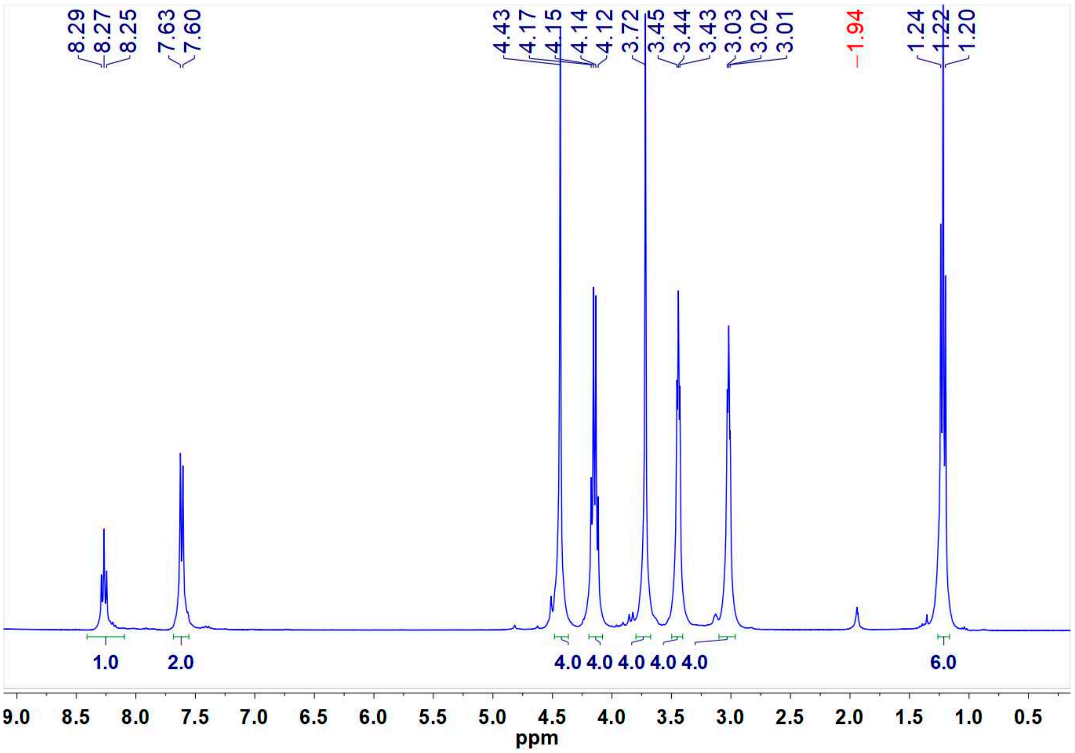

Figure S13. ^{1}H-NMR spectrum of **3,9-diethyl-6-oxa-3,9,15-triazabicyclo[9.3.1]pentadeca-1(15),11,13-triene-3,9-diacetic ester (9)** (Reference: CD_3CN).

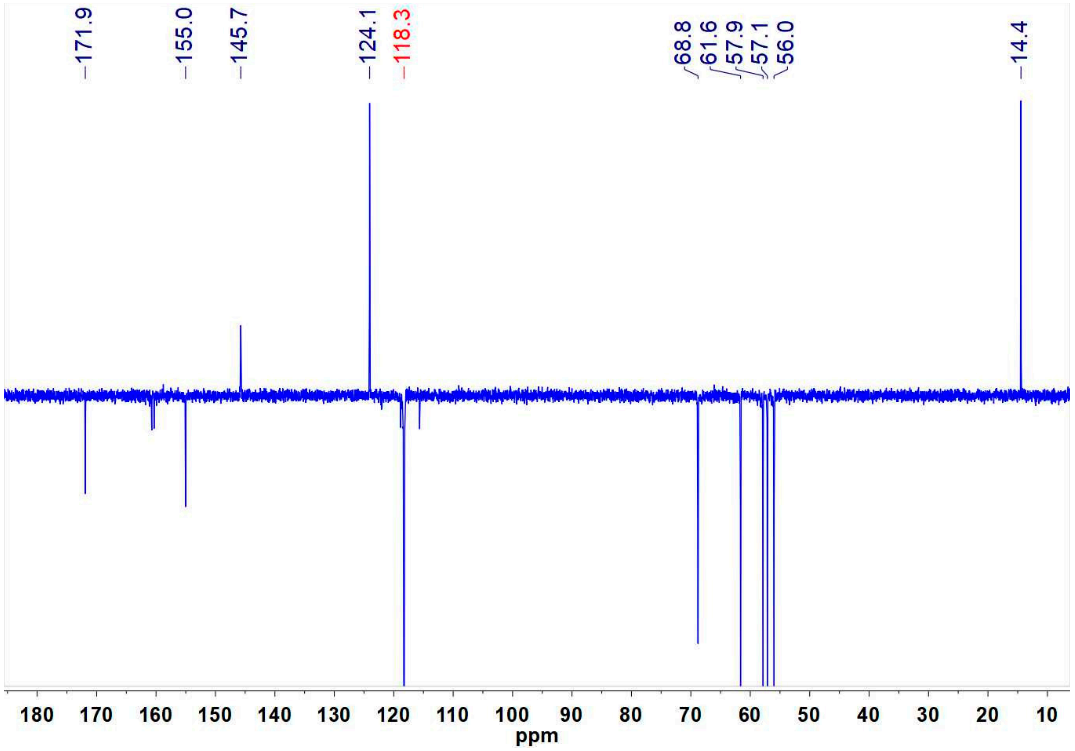

Figure S14. ^{13}C-NMR spectrum of **3,9-diethyl-6-oxa-3,9,15-triazabicyclo[9.3.1]pentadeca-1(15),11,13-triene-3,9-diacetic ester (9)** (Reference: CD_3CN; Contaminated with trifluoroacetic acid, which was used during the preparative HPLC purification).

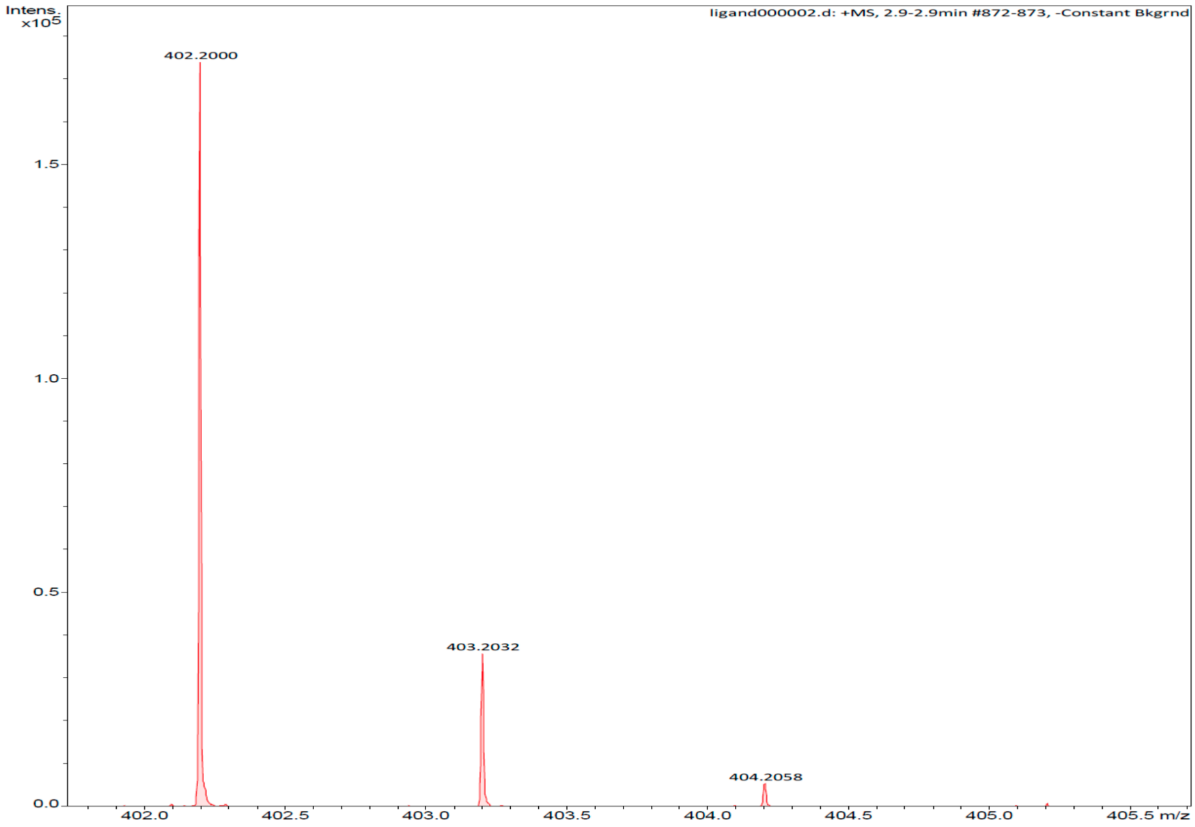

Figure S15. MS spectrum of **3,9-diethyl-6-oxa-3,9,15-triazabicyclo[9.3.1]pentadeca-1(15),11,13-triene-3,9-diacetic ester (9)**.

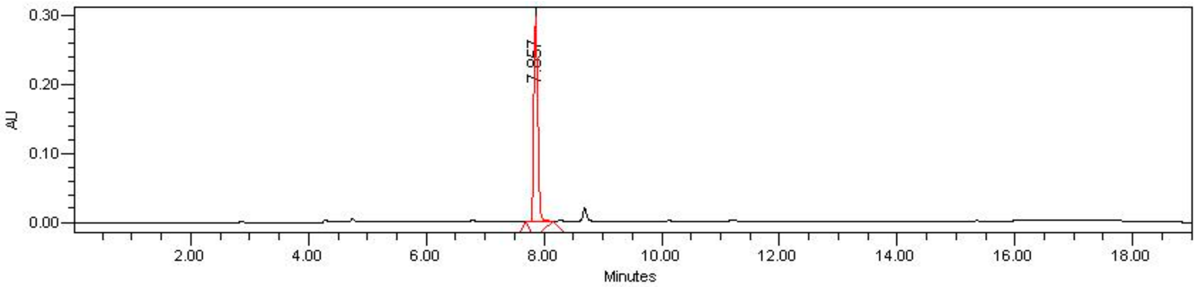

Figure S16. Analytical HPLC chromatogram of **3,9-diethyl-6-oxa-3,9,15-triazabicyclo[9.3.1]pentadeca-1(15),11,13-triene-3,9-diacetic ester (9)** (t_R=7.86 min).

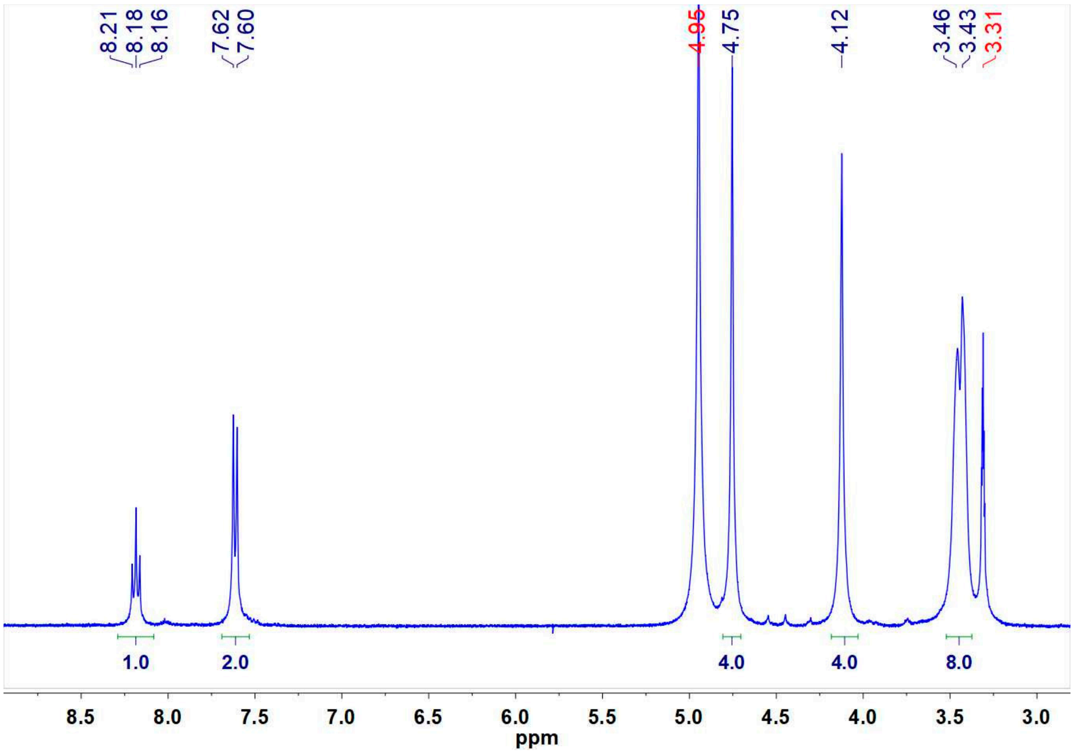

Figure S17. ^{1}H-NMR spectrum of **6-oxa-3,9,15-triazabicyclo[9.3.1]pentadeca-1(15),11,13-triene-3,9-diacetic acid (**H_2**3,9-OPC2A) (10)** (Reference: CD_3OD).

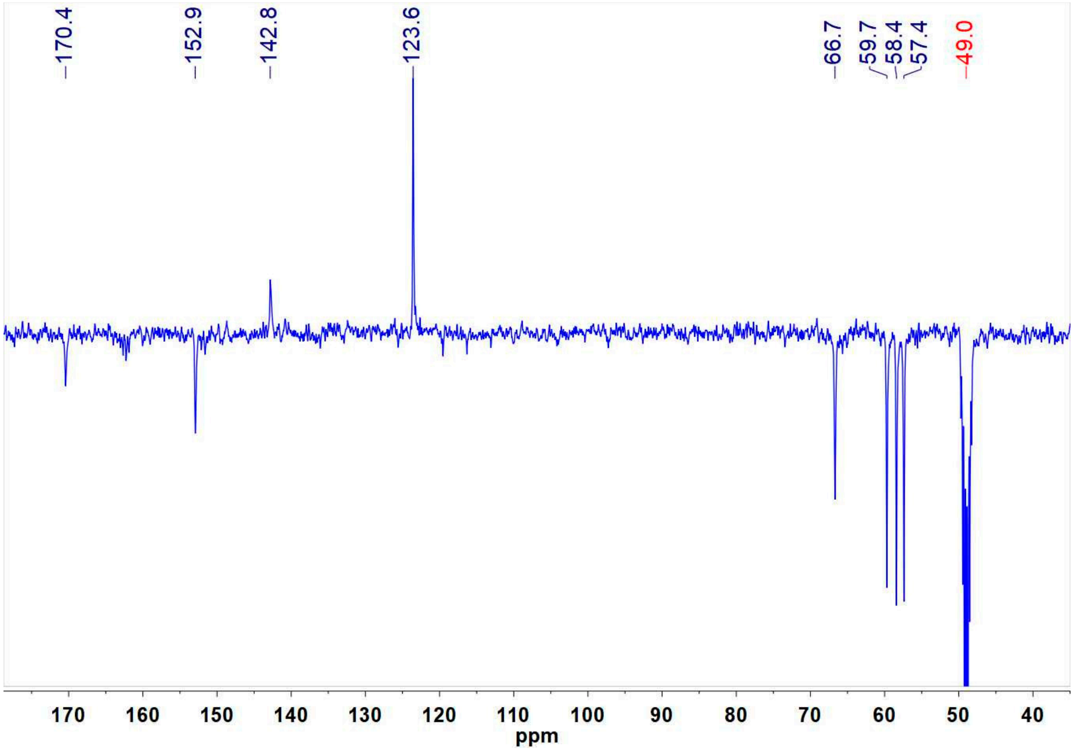

Figure S18. ^{13}C-NMR spectrum of **6-oxa-3,9,15-triazabicyclo[9.3.1]pentadeca-1(15),11,13-triene-3,9-diacetic acid (**H_2**3,9-OPC2A) (10)** (Reference: CD_3OD, Contaminated with trifluoroacetic acid, which was used during the preparative HPLC purification).

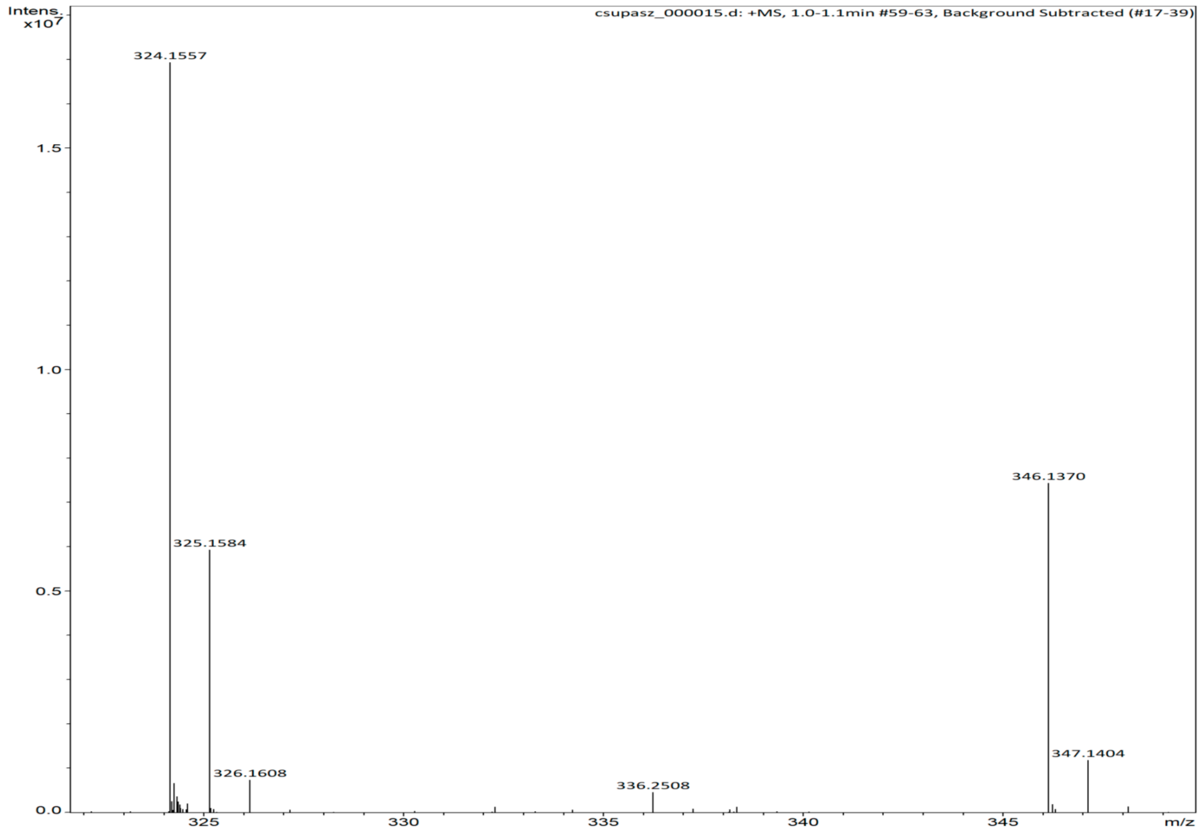

Figure S19. MS spectrum of **6-oxa-3,9,15-triazabicyclo[9.3.1]pentadeca-1(15),11,13-triene-3,9-diacetic acid** (H_2**3,9-OPC2A) (10)**.

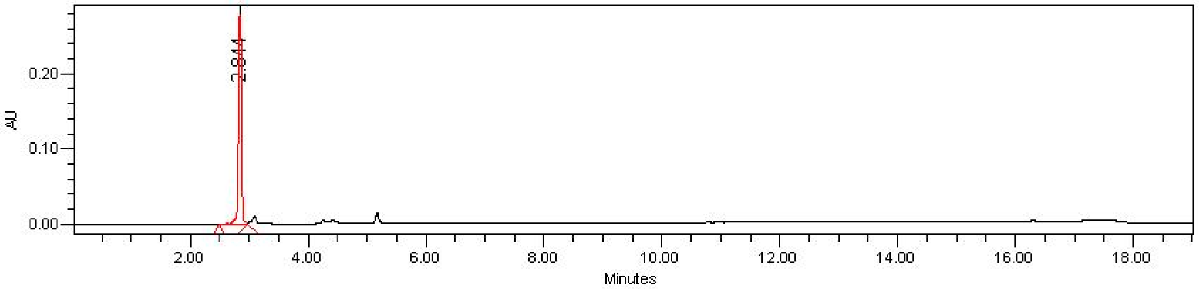

Figure S20. Analytical HPLC chromatogram of **6-oxa-3,9,15-triazabicyclo[9.3.1]pentadeca-1(15),11,13-triene-3,9-diacetic acid** (H_2**3,9-OPC2A) (10)** (t_R=2.84 min).

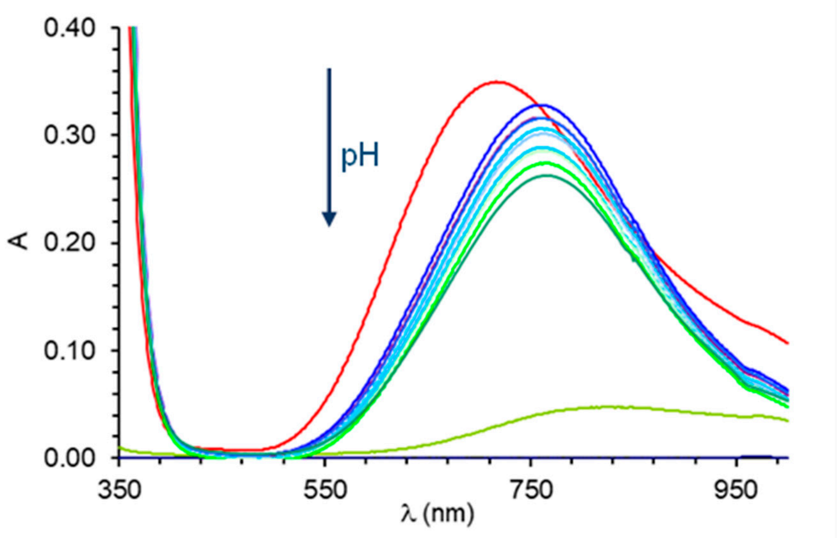

Figure S21. Absorption spectra of the Cu(II)–(**3,9-OPC2A**)–H^+ system ([HCl] + [NaCl] = 1.0 M; [Cu^{2+}] = [**3,9-OPC2A^{2-}**] = 3.06 mM, l = 1 cm, 25 °C). The spectrum in red corresponds to the acid concentration of 13.5 mM, while for the rest of the samples 122, 195, 267, 340, 412, 485, 557, 702, 847 and 993 mM acid was present in the samples, the light green spectrum corresponds to $CuCl_2$ alone.

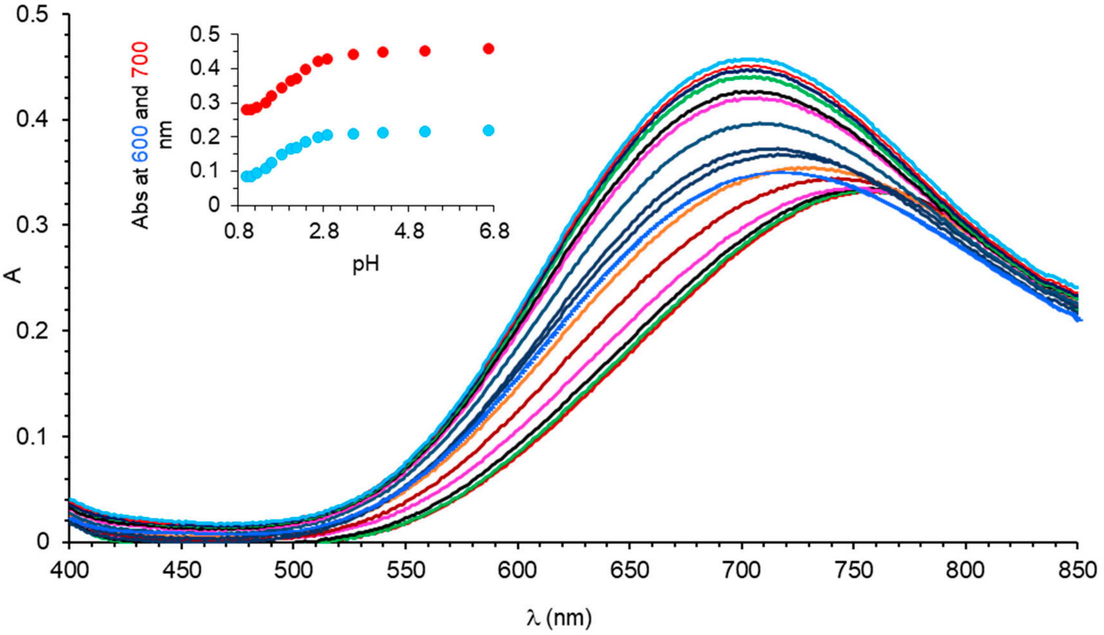

Figure S22. Changes observed in the absorption spectra of the [Cu(**3,9-OPC2A**)] complex as a function of H^+ ion concentration ([HCl] + [NaCl] = 0.15 M; [Cu^{2+}] = [**3,9-OPC2A^{2-}**] = 3.04 mM, l = 1 cm, 25 °C).

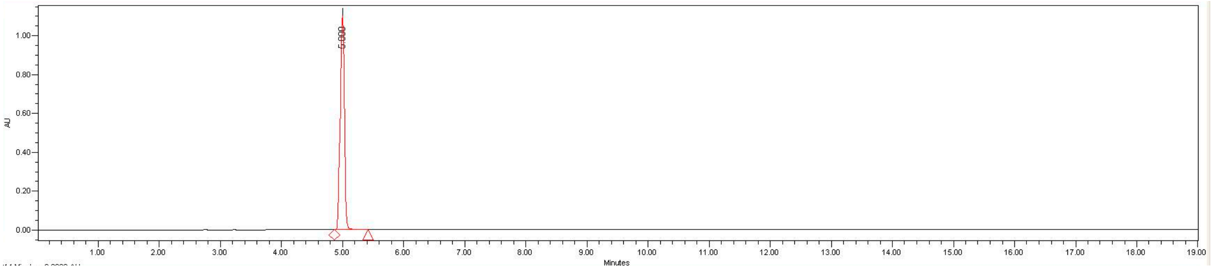

Figure S23. Analytical HPLC chromatogram of **[Mn(3,9-OPC2A)]** (t_R=5.00 min).

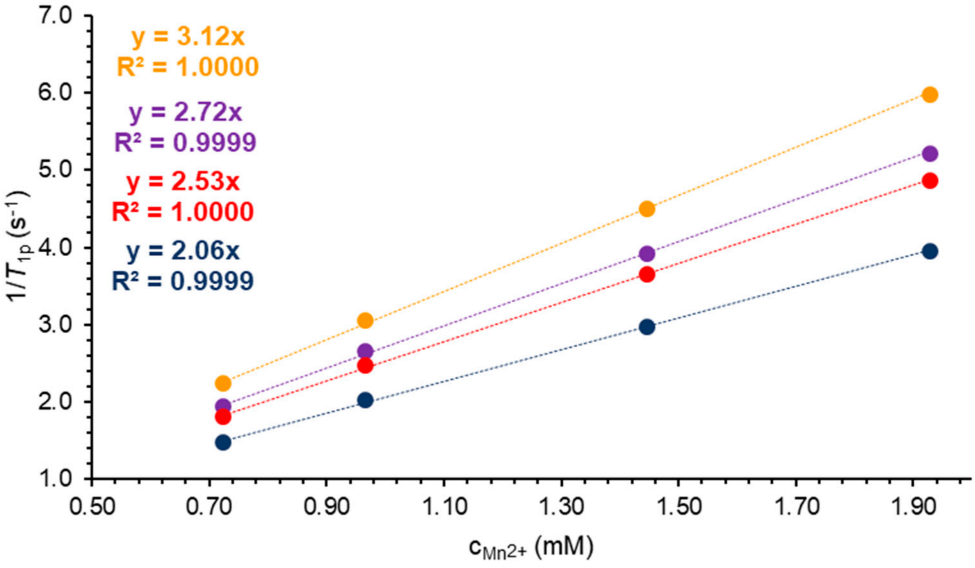

Figure S24. Determination of r_{1p} relaxivity for [Mn(**3,9-OPC2A**)] at 0.49 T (**25 °C**/**37 °C**) and 1.41 T (**25 °C**/**37 °C**) field strength.

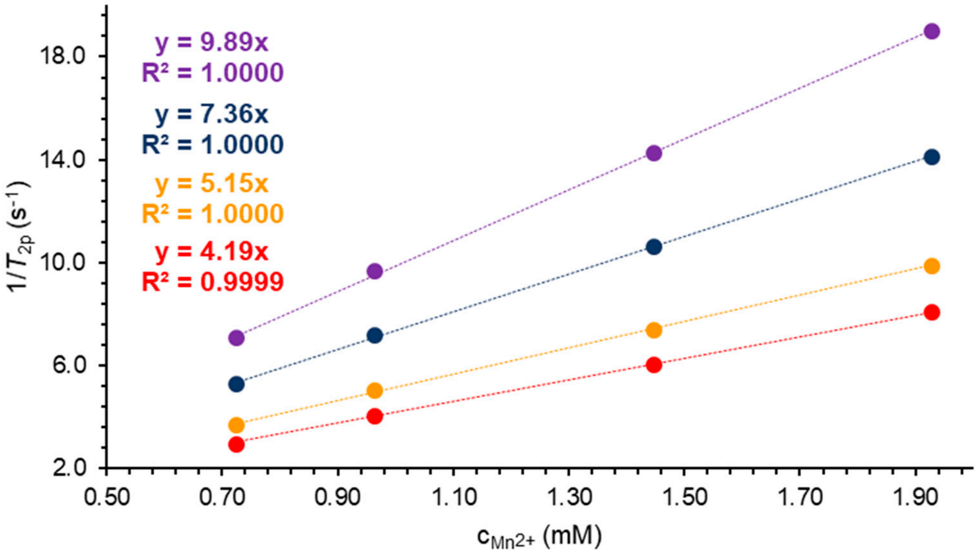

Figure S25. Determination of r_{2p} relaxivity for [Mn(**3,9-OPC2A**)] at 0.49 T (**25 °C**/**37 °C**) and 1.41 T (**25 °C**/**37 °C**) field strength.

S3. Measurements of ^{17}O NMR relaxation rates

The Swift and Connick theory was used to analyze the ^{17}O NMR data to evaluate the reduced transverse ^{17}O relaxation rates calculated from the relaxation rates $1/T_2$ and $1/T_{2A}$ measured for the paramagnetic solutions and the diamagnetic reference [3]:

$$\frac{1}{T_{2r}} = \frac{1}{P_m}\left[\frac{1}{T_2} - \frac{1}{T_{2A}}\right] = \frac{1}{\tau_m}\frac{T_{2m}^{-2} + \tau_m^{-1}T_{2m}^{-1} + \Delta\omega_m^2}{(\tau_m^{-1} + T_{2m}^{-1})^2 + \Delta\omega_m^2} \qquad \text{(S1)}$$

$\Delta\omega_m$ is governed by the hyperfine or scalar coupling constant, $A_O/\hbar$, where B, S and g_L are the magnetic field, the electron spin and the isotropic Landé g factor (Equation (S3)).

$$\Delta\omega_m = \frac{g_L \mu_B S(S+1)B}{3k_B T}\frac{A_O}{\hbar} \qquad \text{(S2)}$$

The ^{17}O transverse relaxation rate is mainly determined by the scalar contribution ($1/T_{2sc}$).

$$\frac{1}{T_{2m}} \cong \frac{1}{T_{2sc}} = \frac{S(S+1)}{3}\left(\frac{A_O}{\hbar}\right)^2 \tau_s \qquad \frac{1}{\tau_s} = \frac{1}{\tau_m} + \frac{1}{T_1} \qquad \text{(S3)}$$

The exchange rate, k_{ex}, (or inverse binding time, τ_m) of the inner sphere water molecule is assumed to obey the Eyring equation (Equation (S5)) where $\Delta S^{\ddagger}$ and $\Delta H^{\ddagger}$ are the entropy and enthalpy of activation for the exchange, and $^{298}k_{ex}$ is the exchange rate at 298.15 K.

$$\frac{1}{\tau_m} = k_{ex} = \frac{k_B T}{h}\exp\left\{\frac{\Delta S^{\ddagger}}{R} - \frac{\Delta H^{\ddagger}}{RT}\right\} = \frac{k_{ex}^{298} T}{298.15}\exp\left\{\frac{\Delta H^{\ddagger}}{R}\left(\frac{1}{298.15} - \frac{1}{T}\right)\right\} \qquad \text{(S4)}$$

For the fit of the ^{17}O T_2 data, an exponential function of the temperature dependency of $1/T_{1e}$ was used:

$$\frac{1}{T_{1e}} = \frac{1}{T_{1e}^{298}}\exp\left\{\frac{E_v}{R}\left(\frac{1}{T} - \frac{1}{298.15}\right)\right\} \qquad \text{(S5)}$$

The ^{1}H relaxivity ($mM^{-1}s^{-1}$) of the Mn(II) complexes is determined by the inner- and outer-sphere contributions (Equation (S7)):

$$r_1 = r_{1is} + r_{1os} \tag{S6}$$

The inner-sphere term is given by Equation (S8), where q is the number of inner-sphere water molecules.

$$r_{1is} = \frac{1}{1000} \times \frac{q}{55.55} \times \frac{1}{T_{1m}^{H} + \tau_m} \tag{S7}$$

In the longitudinal relaxation rate of inner sphere water protons, $1/T_{1m}^{H}$, the dipolar contribution dominates (Equation (S9)):

$$\frac{1}{T_{1m}^{H}} \cong \frac{1}{T_1^{DD}} = \frac{2}{15}\left(\frac{\mu_0}{4\pi}\right)^2 \frac{\hbar^2 \gamma_S^2 \gamma_I^2}{r_{MnH}^6} S(S+1)\left[\frac{3\tau_{d1H}}{1+\omega_I^2\tau_{d1H}^2} + \frac{7\tau_{d2H}}{1+\omega_S^2\tau_{d2H}^2}\right] \tag{S8}$$

Here r_{MnH} is the effective distance between the Mn^{2+} electron spin and the water protons, ω_I is the proton resonance frequency, τ_{diH} is given by Equation S10, where τ_{RH} is the rotational correlation time of the Mn(II)–H_{water} vector:

$$\frac{1}{\tau_{diH}} = \frac{1}{\tau_m} + \frac{1}{\tau_{RH}} + \frac{1}{T_{ie}} \qquad i = 1\ ,2; \tag{S9}$$

$$\tau_{RH} = \tau_{RH}^{298} \exp\left\{\frac{E_R}{R}\left(\frac{1}{T} - \frac{1}{298.15}\right)\right\} \tag{S10}$$

The electronic relaxation is mainly governed by modulation of the transient zero-field splitting, and for the electron spin relaxation rates, $1/T_{1e}$ and $1/T_{2e}$, McMachlan has developed Equations (S12)–(S14), which were used in the fit of the NMRD data [4]:

$$\left(\frac{1}{T_{1e}}\right) = \frac{32}{25}\Delta^2\left(\frac{\tau_v}{1+\omega_S^2\tau_v^2} + \frac{4\tau_v}{1+4\omega_S^2\tau_v^2}\right) \tag{S11}$$

$$\left(\frac{1}{T_{2e}}\right)=\frac{32}{50}\Delta^2\left[3\tau_v+\frac{5\tau_v}{1+\omega_S^2\tau_v^2}+\frac{2\tau_v}{1+4\omega_S^2\tau_v^2}\right] \tag{S12}$$

$$\tau_v=\tau_v^{298}\exp\left\{\frac{E_v}{R}\left(\frac{1}{T}-\frac{1}{298.15}\right)\right\} \tag{S13}$$

where Δ^2 is the trace of the square of the transient zero-field-splitting (ZFS) tensor, τ_v is the correlation time for the modulation of the ZFS with the activation energy E_v, and ω_S is the Larmor frequency of the electron spin.

The outer-sphere contribution to the overall relaxivity is described by Equation (S15), where N_A is the Avogadro constant, and J_{os} is a spectral density function (Equation (S16)).

$$r_{1os}=\frac{32N_A\pi}{405}\left(\frac{\mu_0}{4\pi}\right)^2\frac{\hbar^2\gamma_S^2\gamma_I^2}{a_{MnH}D_{MnH}}S(S+1)[3J_{os}(\omega_I,T_{1e})+7J_{os}(\omega_S,T_{2e})] \tag{S14}$$

$$J_{os}(\omega,T_{je})=\mathrm{Re}\left[\frac{1+\frac{1}{4}\left(i\omega\tau_{MnH}+\frac{\tau_{MnH}}{T_{je}}\right)^{1/2}}{1+\left(i\omega\tau_{MnH}+\frac{\tau_{MnH}}{T_{je}}\right)^{1/2}+\frac{4}{9}\left(i\omega\tau_{MnH}+\frac{\tau_{MnH}}{T_{je}}\right)+\frac{1}{9}\left(i\omega\tau_{MnH}+\frac{\tau_{MnH}}{T_{je}}\right)^{3/2}}\right]$$

$$j=1,2 \tag{S15}$$

The diffusion coefficient for the diffusion of a water proton away from a Mn(II) complex, D_{MnH}, obeys the exponential temperature dependence described by Equation (S17), with activation energy E_{MnH}:

$$D_{MnH}=D_{MnH}^{298}\exp\left\{\frac{E_{MnH}}{R}\left(\frac{1}{298.15}-\frac{1}{T}\right)\right\} \tag{S16}$$

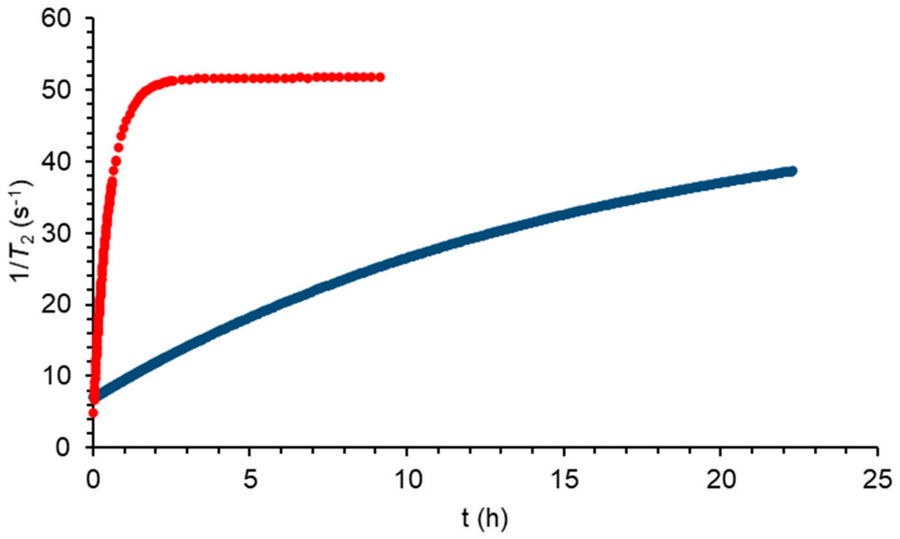

Figure S26. The 1/T_2 values as a function of time for the dissociation of [Mn(**3,9-OPC2A**)] (●) and [Mn(**3,9-PC2A**)] (●) complexes by using 25-fold Zn(II) exchanging metal ion at pH=6.0.

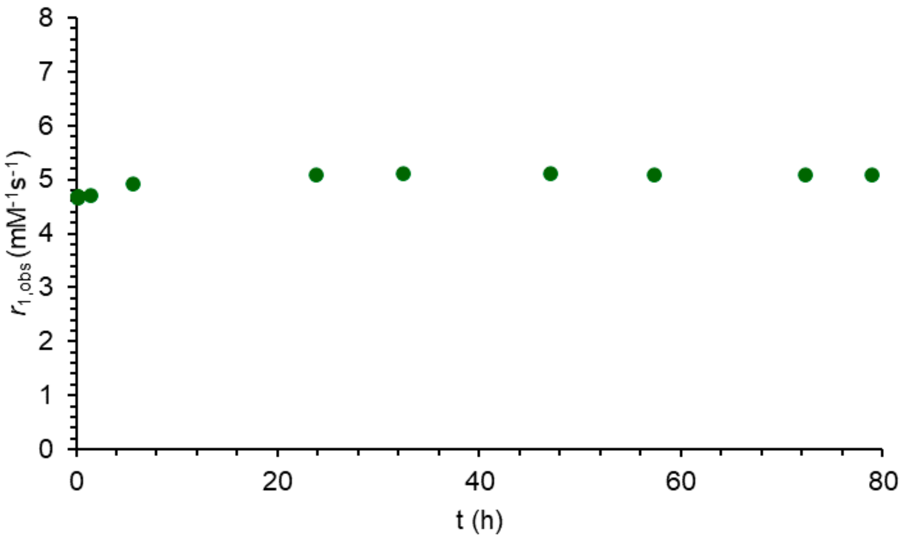

Figure S27. Stability investigation of the [Mn(**3,9-OPC2A**)] complex in Seronorm solution.

Table S1. XYZ coordinates (in Å) and total electronic energies (in Hartree) of [Mn(**3,9-OPC2A**)] and [Mn(**3,9-PC2A**)] complexes.

[Mn(**3,9-OPC2A**)]

E(TPSSh-D3BJ/def2-TZVPP)= -2484.727652367632

Mn	0.01881860674387	0.43506762516311	0.14322789724173
N	-1.02980601221044	-1.19996428010990	-0.48436317342908
C	-0.68114822103737	-2.49875718519593	-0.34077988997429
C	-1.52677353024222	-3.53905408879236	-0.69584477609115

C	-2.77906118096270	-3.25527294606633	-1.22697535393403
C	-3.14045403997197	-1.92160382906423	-1.38414963559524
C	-2.24876098022462	-0.92867021284496	-1.01335178575937
C	-2.54572288951325	0.52567994687167	-1.21508095636416
N	-1.85013514488709	1.33392300413630	-0.19080162421719
C	-2.58978950328385	1.38558428626709	1.10591589699344
C	-2.09891067537805	0.31530630840598	2.06014762881982
N	-0.62573251867340	0.42425703562273	2.11769011273005
C	-0.00280680133262	-0.51361871818679	3.07970194722690
C	0.18213280835214	-1.87847849871465	2.44581178850190
N	0.98055137726080	-1.73455987133680	1.22982787964360
C	0.68889371045975	-2.73575190249359	0.21420006473045
C	-1.57074287276361	2.70664339375173	-0.66679116424468
C	-0.33953144109569	3.27378325233602	0.04162994799834
O	-0.18209996472986	4.50260517900161	0.09756669960847
O	0.48892611652169	2.40138476572794	0.48881372973613
O	2.36299405593589	-1.16779880651248	-2.63959610561570
O	0.49784543200101	0.71839978962270	-1.89581788501795
O	2.67655849070359	2.40725369203586	-1.53569440054435
H	-4.10724820925231	-1.64382100630888	-1.78398407239536
H	-3.46050374804213	-4.05073034039143	-1.49972754284986
H	-1.19871096230720	-4.55942406136868	-0.54369964050399
H	0.77955314053688	-3.76516986696282	0.58567990142013
H	1.41848217567848	-2.61356476183993	-0.59274236861632
H	-2.14187679200606	0.82450629705129	-2.18363191256359
H	-3.62264266874999	0.71420224795858	-1.21927023618396
H	-2.35951332701914	-0.68369474853087	1.71332593256428
H	-3.66531008815603	1.30509593258144	0.93252485547245
H	-2.43285380860212	3.36401286466969	-0.53518724609971
H	-1.33435356995744	2.65975040609413	-1.73076738408346
H	-2.54909332898238	0.46492916136997	3.04654401698847
H	-0.40215905399272	1.36736178039158	2.43093426862076
H	-0.60717822547783	-0.59097556360416	3.98860698392353
H	0.96468530939341	-0.09262552871934	3.35520754214787

H 1.13363005636670 1.47035747968417 -1.90302818170238
H 1.07083214757572 -0.03652506291143 -2.17741620845002
H 2.75651239849463 -0.87150042823894 -3.46991516616201
H 3.06539422800325 -1.04621473757270 -1.96653137870263
H 2.70203222287050 1.65876487290909 -0.88241799794945
H 2.30910350277140 3.13470195778887 -1.01627592936020
C 2.41520989345937 -1.59041540896410 1.44089744405862
H 2.93873758547383 -2.55266123120060 1.44682884792492
H 2.59049665805356 -1.09864001781655 2.39961356020091
C 2.95925683934794 -0.67715935770431 0.34503815350574
O 3.98716024917299 -0.99027229076691 -0.29637661972879
O 2.27029245139876 0.37389772003811 0.14790666250906
H 0.63325410207808 -2.57341251066022 3.16538480987900
H -0.78814517212016 -2.29080047025722 2.16170007238102
H -2.39478582768195 2.36346273365750 1.54984399131126

[Mn(**3,9-PC2A**)]

E(TPSSh-D3BJ/def2-TZVPP)= -2504.58162243

C -3.33264478804513 -2.17242072783591 -1.17929046963665
C -2.37113445473046 -1.17468483369930 -1.06312188600864
C -1.04882174742261 -1.52908128876808 -0.85568990344745
N -0.64127289742204 -2.81972023002551 -0.75973935912666
C -1.58438569550684 -3.78494391401504 -0.85238314816182
C -2.92488144586367 -3.49484653647306 -1.06092578684554
C 0.04311881180815 -0.51263185447717 -0.73232174875059
N 1.10251983482588 -1.03113819430362 0.16507292531221
C 2.42002731778984 -0.41146633225854 -0.12239116734374
C 3.54640334866255 -1.37269410060883 0.25876409825396
O 3.23872650605880 -2.61985716766431 0.21567887351853
Mn 1.32867362161245 -3.03830185261645 -0.27356559138549
O 1.77280277484100 -2.51628482604635 -2.31233183074638
C -1.08901455374262 -5.19158687831713 -0.71803701095005
N -0.06905179615599 -5.26108461125931 0.31792450900668
C 0.82623116940717 -6.38599113079925 0.11178811740155

C	1.78785837853526	-6.01756047846038	-1.01224559790128
O	2.11566445803101	-6.84798436231838	-1.88104452649889
C	0.78178497757067	-0.85090757244781	1.61315491361771
C	0.08789831769109	-2.06204258013296	2.18592916337508
O	0.94676566965091	-3.17452961917396	1.87836278856467
C	0.53843744959339	-4.41166190142071	2.49257276929275
C	-0.55498401139104	-5.08042233362749	1.68004837453884
O	4.67135701748146	-0.92469692568826	0.52044358688207
O	2.20121923732084	-4.80863083811457	-0.98897498286044
O	0.99464860668696	-4.75122555595408	-3.74849671368716
O	4.28613482174057	-3.73715817304023	-2.31892280955446
H	-2.63725692130148	-0.12706044471733	-1.11769105226517
H	-3.63614339626793	-4.30893658378613	-1.11647761243200
H	-4.37456932428938	-1.92386694037725	-1.33421584051499
H	-0.34477892635560	0.44942598614898	-0.38786252482619
H	0.50028232894439	-0.37006702655016	-1.71212345918541
H	-0.61979442892852	-5.48522040558750	-1.66251508710582
H	-1.92951374876351	-5.87617398053648	-0.54482291711566
H	0.17881136750571	0.04858526560867	1.75817330017484
H	1.72671506017526	-0.71969849731653	2.13969415701841
H	-0.85501787890064	-6.02210100896516	2.15899153072830
H	-1.43647618678893	-4.43734243057701	1.64592811504503
H	2.49207180900978	-0.24413742902172	-1.19782378734301
H	2.53120081212029	0.54861416339391	0.38466757501444
H	1.42021263835112	-6.54881211774984	1.01393269089764
H	0.29931910210055	-7.31828179773526	-0.12156871706020
H	2.73534876731906	-2.69739278372567	-2.40217944524275
H	1.37914937510574	-3.24679667627560	-2.84901494163042
H	1.51035681775561	-5.44938176678600	-3.30369798596611
H	1.33148640759094	-4.73287401985557	-4.65320156227258
H	-0.00092656847813	-1.96370370711616	3.27040546506024
H	-0.90462709075992	-2.21860055195481	1.75951522286636
H	1.43736180565451	-5.02673860453871	2.52218548462012
H	0.21309250313881	-4.21056855197615	3.51541234119967

H	3.60856888228842	-4.29751706038406	-1.86327730966368
H	4.71956486474617	-3.28950721007142	-1.57975822685977

Table S2. Mulliken spin populations for relevant atoms.

atom	1	2
Mn	1.05	1.05
N1	-0.01	-0.01
N3	0.02	0.03
N6/O6	-0.02	0.00
N9	-0.02	-0.02
O3	0.00	0.00
O9	-0.01	-0.01
O1w	-0.01	-0.01

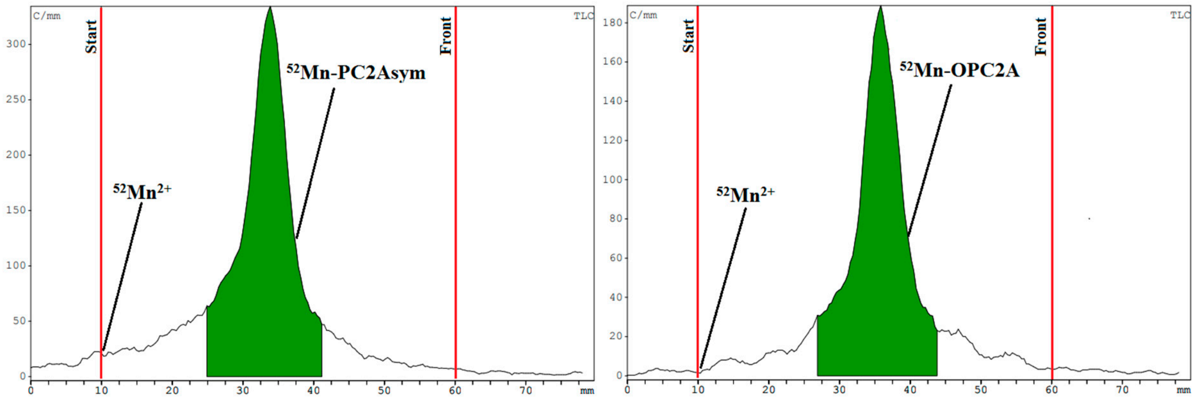

Figure S28. Radio-TLC chromatograms of the purified [[^{52}Mn]Mn(**3,9-PC2A**)] and [[^{52}Mn]Mn(**3,9-OPC2A**)] complexes using TLC Silica gel 60 plate with a 2:1 mixture of water and acetonitrile as the mobile phase.
